# Comparative Analysis of Morphological and Acoustic Correlates of Bush-Cricket Tympanic Membranes

**DOI:** 10.34133/csbj.0035

**Published:** 2026-04-09

**Authors:** Md Niamul Islam, Lewis B. Holmes, Dominic Rooke, Fabio A. Sarria-S, Fernando Montealegre-Z

**Affiliations:** School of Natural Sciences, University of Lincoln, Lincoln, Lincolnshire, UK.

## Abstract

Bush-crickets exhibit remarkable auditory diversity, having a pair of tympanic membranes (tympana) on each foreleg. These tympana could be exposed or covered by cuticular flaps known as auditory pinnae. These membranes receive sound directly and via an acoustic tracheal tube. Despite extensive research on auditory tuning, the relationships between the tympanal morphology, body size, and acoustic traits of bush-crickets remain unclear. In this study, micro-computed tomography reconstructions, artificial-intelligence-assisted segmentation, and phylogenetically informed comparative regressions in 18 selected bush-cricket species were used to investigate how tympanal surface area and mean cross-sectional thickness relate to pronotum length (a proxy for body size), carrier frequency, and the presence of auditory pinnae. Tympanal surface area scaled positively with pronotum length, whereas thickness was independent of size. Carrier frequency showed a weak negative relationship with tympanal thickness, although this association was not statistically significant. Among 14 species with well-developed auditory pinnae, tympanal dimensions showed no correlation with peak resonance frequency, suggesting that pinnal cavity resonance and tympanal morphology are not tightly correlated and may evolve independently. Across species, the presence of pinnae was not associated with differences in tympanal surface area, although unilateral pinnae were linked to thicker tympana. Within the 4 unilateral species, the pinna-covered tympanum remained consistently larger in area but thinner than the exposed side. Overall, these findings indicate that tympanal evolution reflects a balance of scaling constraints and configuration-specific associations with auditory pinnae.

## Highlights

• Micro-computed tomography with artificial-intelligence-assisted segmentation enabled precise 3-dimensional measurements of tympana.
• Tympanal surface area scales positively with body size.
• Pinna resonance shows no trend with tympanal surface area or thickness.
• Species with unilateral pinnae tend to have thicker tympana.
• Pinna-covered tympana are larger yet thinner than exposed sides within species.

## Graphical Abstract

**Figure:**
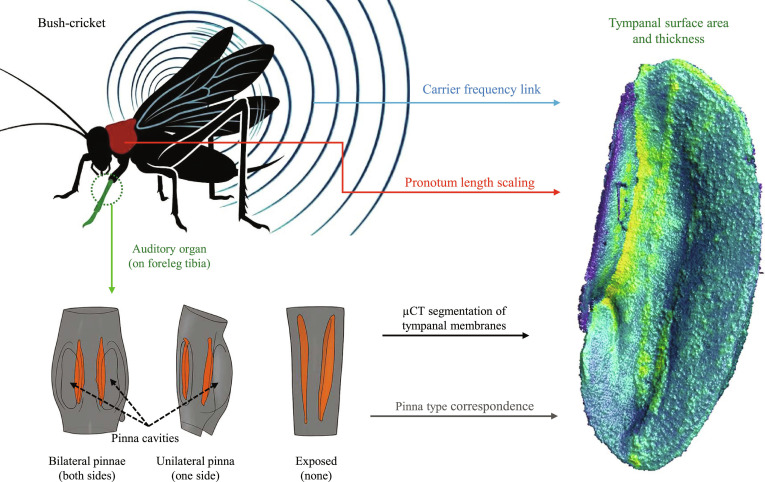

## Introduction

Bush-crickets (Orthoptera: Tettigoniidae) possess hearing organs on their forelegs, located on the proximal tibia just distal to the femur–tibia joint. Each ear includes a pair of tympanic membranes (tympana) located on the anterior and posterior surfaces of the proximal tibia, forming an auditory system that shows parallels to certain processing features found in mammalian hearing [1–3]. However, unlike mammals, sound reaches these membranes through 2 routes: directly from the surrounding air and indirectly via the acoustic trachea, an internal air-filled tube that connects the thoracic spiracle to the ear through the leg [4,5]. For both pathways, the tympana convert sound pressure stimuli into mechanical vibrations [6,7], which are transmitted to the crista acustica for frequency decomposition and further directional interpretation by the central nervous system [2,8,9].

**Fig. 1.**
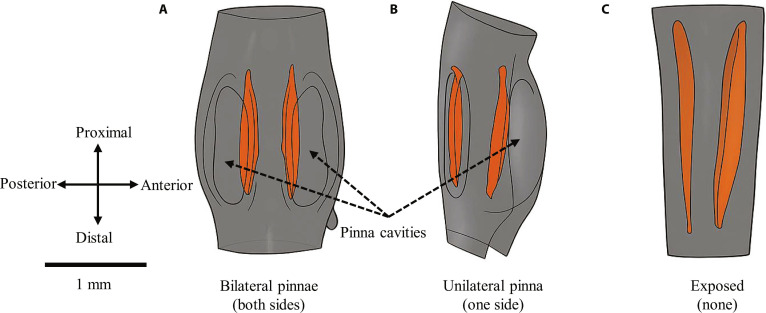
Variations in pinna configuration among bush-crickets. (A) Bilateral pinnae of *Copiphora gorgonensis*, with both tympana enclosed within paired pinna cavities. (B) Unilateral pinna of *Stictophaula* sp., where only one tympanum is covered while the opposite remains exposed. (C) Exposed tympana of *Mecopoda elongata*, where both tympanic membranes are uncovered and lack any pinna structures. Orange regions indicate the tympanic membranes, and dashed arrows highlight the positions of the pinna cavities.

According to the Orthoptera Species File database, the family Tettigoniidae currently comprises more than 8,500 valid extant species worldwide, exhibiting a broad range of morphological diversity [10]. Their use of ultrasonic frequencies for long-range acoustic communication makes them an interesting subject for bioacoustics research [11,12]. However, acoustic signaling also increases exposure to eavesdropping predators, particularly gleaning bats, which can locate singing bush-crickets by listening to their calling songs [13–17]. To keep up in the prey–predator arms race, many bush-cricket species have evolved cuticular flaps known as auditory pinnae (Fig. 1A), which act as natural “bat detectors”. The prevailing view is that the ancestral function of auditory pinnae was protective, shielding the tympanal membranes [18–21]. Recent studies suggest that pinnal cavities can generate frequency-specific sound pressure gain corresponding to early bat echolocation calls [22,23]. Interestingly, within the Phaneropterinae, some taxa possess a unilateral pinna (Fig. 1B), producing an asymmetry with one pinna-covered and one exposed tympanum [24,25]. In contrast, several groups such as other Phaneropterinae and Phlugidini taxa entirely lack pinnae (Fig. 1C), leaving both tympana exposed [26–30]. This diversity in pinna configuration may reflect differences in ecological or selective pressures across lineages. Possible explanations include variation in bat predation intensity, habitat acoustics, or trade-offs between mechanical protection and acoustic gain.

The key morphological traits of tympana are surface area and thickness, as larger surface areas can capture more sound energy, whereas membrane thickness influences frequency response and sensitivity, while local variation in thickness may affect vibration damping and fine-scale mechanical behavior [9,31]. Across tettigoniids, there is great variation in body size, ear morphology, acoustic function, and tympanal membrane morphology [32–37]. Comparative and experimental studies across Orthoptera have demonstrated substantial diversity in auditory tuning and mechanical specialization across multiple lineages [38–44]. This raises the question of whether exposed tympana are thicker than those protected by pinnae. Despite extensive research into bush-cricket auditory tuning, auditory mechanics, and sensory organization of the ear, a research gap remains in understanding how tympanal morphology relates to other structural and acoustic characteristics. This limitation likely stems from the extremely thin and delicate nature of the tympanal membranes, which makes precise morphological characterization challenging. However, recent advances in high-resolution micro-computed tomography (μCT) and artificial intelligence (AI)-assisted segmentation allow accurate quantification of tympanal geometry and thickness across species [45].

This study investigated the association of body size, acoustic traits, and external ear morphology (pinna configuration) with tympanal structural features through comparative analysis of multiple species. Specifically, the relationships between pronotum length (as a proxy for body size), carrier (dominant) frequency of the call, pinna resonance frequency, pinna presence (exposed, unilateral, and bilateral), and tympanal surface area and thickness were assessed. Additionally, asymmetries between pinna-covered and exposed tympana in unilateral species were examined as understanding these structural relationships may also inform future bioinspired acoustic designs.

## Methods

### Specimen selection

Eighteen different species were investigated in this study, representing a diverse range of pinna configurations and subfamilies (Table 1; full morphological and acoustic data are provided in Supplementary Material S1, Table S1). Specimens were obtained from laboratory colonies or collected from the field. Only adult males were analyzed to avoid the potential effects of sexual dimorphism reported in some taxa. Specimens were selected based on the availability of well-preserved specimens suitable for high-resolution μCT imaging and with documented acoustic data. The sampling was designed to capture diversity in pinna configuration (bilateral, unilateral, and absent) across multiple subfamilies rather than to provide exhaustive phylogenetic coverage of Tettigoniidae. In the unilateral taxa examined, the pinna was typically located over the anterior tympanum, leaving the posterior tympanum exposed. While many tettigoniids exhibit broadband signals, the species in this dataset primarily produce narrowband, pure-tone calls. Consequently, carrier frequency was utilized as the primary acoustic metric, defined as the peak or dominant frequency component of the calling song. Carrier frequency values were obtained either from recordings conducted in our lab or from published sources, as detailed in Supplementary Material S1, Table S1.

**Table 1.** Summary of the 18 bush-cricket species investigated, including their location, subfamily classification, and auditory pinna configuration

Species	Location	Subfamily	Pinna type
*Arachnoscelis* sp.	South America	Meconematinae	Bilateral
*Arnobia pilipes*	Asia	Phaneropterinae	Unilateral
*Balboana tibialis*	South America	Pseudophyllinae	Bilateral
*Chibchella nigrospecula*	South America	Pseudophyllinae	Bilateral
*Copiphora gorgonensis*	South America	Conocephalinae	Bilateral
*Elimaea signata*	Asia	Phaneropterinae	Bilateral
*Haenschiella* sp.	South America	Pseudophyllinae	Bilateral
*Leptoderes ornatipennis*	Asia	Phaneropterinae	Exposed
*Mecopoda elongata*	Asia	Mecopodinae	Exposed
*Moncheca elegans*	South America	Conocephalinae	Bilateral
*Phaulula galeata*	Asia	Phaneropterinae	Unilateral
*Phlugis poecilla*	South America	Meconematinae	Exposed
*Phygela marginata*	Asia	Phaneropterinae	Unilateral
*Phyllomimus detersus*	Asia	Pseudophyllinae	Bilateral
*Ragoniella pulchella*	South America	Conocephalinae	Bilateral
*Satizabalus jorgevargasi*	South America	Pseudophyllinae	Bilateral
*Stictophaula* sp.	Asia	Phaneropterinae	Unilateral
*Stilpnochlora* sp.	South America	Phaneropterinae	Unilateral

### Specimen preparation

The insects were frozen at −20 °C for approximately 15 min before dissection or used after dying naturally following experimental or behavioral observations. Forelegs were then removed for preparation before μCT imaging. Each leg was transferred through ethanol in 20% hourly increments from 20% to 80%. Specimens were then submerged for 24 h in 1% Lugol’s iodine solution to enhance tissue contrast for μCT imaging. After staining, samples were washed 3 times in 70% ethanol to remove excess iodine and subsequently submerged in hexamethyldisilazane overnight. The stepwise ethanol dehydration followed by hexamethyldisilazane drying was used to minimize tissue distortion and shrinkage during μCT preparation. Finally, the legs were desiccated for 24 h under a fume hood to remove residual liquid and maintain structural integrity for μCT scanning.

### μCT imaging and volumetric reconstruction

Specimens were scanned using a SkyScan 1172 μCT scanner (Bruker Corporation, Billerica, MA, USA). Groups of 3 to 4 legs were placed inside a tube and mounted in the scanner, and the entire tube was imaged as a batch. Scans extended from the tip of the tarsal claws to the top of the foretibiae. The scan resolution was set to 4,032 × 2,688 pixels, with an isotropic voxel size of 2 to 3 μm and a rotation step of 0.1°. Current and voltage were held constant across all scans at 150 mA and 40 kV, respectively, with an exposure time of 800 ms. Raw projection data were reconstructed into orthogonal slices using nRecon (v.1.6.9.18, Bruker Corporation, Billerica, MA, USA), with identical reconstruction parameters applied to each scan. Volumetric reconstructions were generated in the Dragonfly 3D software (Object Research Systems, Montreal, Canada).

### Three-dimensional printing and resonance acoustic testing

Stereolithography was used to fabricate physical models of the bush-cricket outer ear with pinna cavities, scaled 10× to 15× from the original anatomy. Printing was performed on an Elegoo Saturn printer (Elegoo, Shenzhen, China) using the ChituBox slicer software. Models were produced in acrylonitrile butadiene styrene resin (ABS-Like 2.0 gray resin) with a layer height of 0.06 mm. The bottom layers were exposed for 30 s (first 5 layers), followed by 7.5 s for subsequent layers. The printed parts were rinsed in 100% isopropyl alcohol and cured in an ultraviolet curing station (GeeeTech, Shenzhen, China) for 1 h.

The pinna cavities of the printed specimen were drilled with a 2-mm-radius drill bit to make the cavity accessible for the probe microphone. A 25-mm-tipped B&K Type 4182 probe microphone (Brüel & Kjær, Nærum, Denmark), with a 1 × 25 mm (0.99″) probe tube length and a 1.24-mm (0.05″) internal diameter, was used to record cavity pressure. According to the manufacturer, this probe microphone has a smooth frequency response between 1 Hz and 20 kHz. The microphone was calibrated using a B&K Type 4237 sound pressure calibrator (94 dB at 1 kHz). The microphone was pushed through the drilled hole to the inner wall edge of the cavity, without touching the samples, using an electronic micromanipulator (TR10/MP-245, Sutter Instrument, Novato, CA, USA) to prevent any acoustic distortions inside the cavity. The received signals were amplified using a B&K 1708 conditioning amplifier (Brüel & Kjær, Nærum, Denmark), acquired using a PSV-500 internal data acquisition board at a sampling frequency of 256 kHz. The sampling rate was determined by the PSV acquisition settings and provided sufficient oversampling to ensure accurate spectral analysis and avoid aliasing artifacts.

An Avisoft ultrasonic speaker Vifa with a SPEAKON connector (Avisoft-UltraSoundGate, Glienicke/Nordbahn, Germany), positioned 30 cm from the samples and secured on a magnetic stand, was used to deliver acoustic stimuli. The loudspeaker was connected to a portable ultrasonic power amplifier (Avisoft-UltraSoundGate, Glienicke/Nordbahn, Germany) to ensure controlled sound delivery. The loudspeaker and probe microphone were connected to a PSV-500 internal data acquisition board (Polytec, Waldbronn, Germany), which interfaced with a computer running the Polytec 10.1.1 software for system control and data acquisition. To match the scale of the printed models, a broadband stimulus consisting of periodic chirps with a simulated frequency range of 2 to 20 kHz was delivered by the loudspeaker, representing the natural range of 20 to 200 kHz for 10× scaled models and 30 to 300 kHz for 15× scaled models. To ensure an initial flat stimulus intensity at all frequencies, the amplitude of the broadband signal was mathematically corrected within the software, providing a uniform sound pressure level of 40 dB SPL across all frequencies. This moderate stimulus level was selected to maintain linear loudspeaker performance and avoid distortion, as the analyses focused on relative frequency-dependent gain rather than absolute sound pressure sensitivity. The data were postprocessed and analyzed using the Polytec 10.1.1 software, and the peak pinna resonance frequency for each species is listed in Table S2 of Supplementary Material S1.

### Morphometric measurement and analyses

Tympanal membranes were segmented in the Dragonfly 3D software using deep segmentation (AI-assisted) based on a U-net architecture, followed by manual refinement to ensure accuracy [45]. Segmentation performance for the tympanal region achieved Dice similarity coefficients ranging from 0.62 to 0.75 across species, calculated against manually annotated ground-truth masks generated by slice-by-slice delineation of cuticular boundaries. AI-assisted segmentation was used to accelerate the annotation process, and all segmentations were visually inspected and corrected before thickness extraction to minimize potential segmentation artifacts. To standardize measurements and reduce computational processing time, a single foreleg per species (typically the right leg) was used for analysis, as each leg contains 2 tympana, allowing for the extraction of mean surface area and mean thickness distributions. Tympanal surface area was calculated directly from the segmented 3-dimensional (3D) models, and membrane thickness was quantified as the mean thickness distribution of each tympanum. Summarized mean values are presented in Table S1 of Supplementary Material 1, while full thickness distribution data and segmentation outputs for all 18 species are provided in Supplementary Material 2. Mean thickness values were used as a standardized species-level summary metric for comparative analyses, as regressions were performed on interspecific values rather than voxel-level distributions. Although spatial heterogeneity in membrane thickness is evident (see Supplementary Material 2), the mean provided a consistent and biologically interpretable descriptor for cross-species comparisons. In unilateral-pinna species, exposed and pinna-covered tympana were analyzed separately to assess within-species asymmetry (Table S3, Supplementary Material 1). For interspecific ordinary least squares (OLS) and phylogenetic generalized least squares (PGLS) analyses, a single species-level value was retained. In unilateral taxa, this was calculated as the average of the exposed and pinna-covered tympana to maintain comparability with symmetric species.

All morphometric and acoustic variables were natural log-transformed before analysis to linearize potential allometric (power-law) relationships and to allow regression slopes to be interpreted as scaling exponents. This transformation also improves comparability among traits measured on different scales. Pronotum length was used as a proxy for body size, as it is a standard and repeatable metric in Orthoptera comparative studies and is less susceptible to preservation-related distortion than pronotum width. Carrier frequency was defined as the peak frequency of the male calling song. The reported values correspond to the dominant frequency component of each species’ call. OLS and PGLS regression were used to model tympanal surface area, tympanal thickness, and carrier frequency as response variables, with pronotum length, carrier frequency, pinna resonance frequency, and pinna presence included as predictors where appropriate. Standardized residuals were used to visualize partial relationships after accounting for covariates in multiple regression models (e.g., controlling for pronotum length or carrier frequency). This approach allows trait associations to be examined independently of shared variation with other predictors. Residuals centered on zero indicate values close to those predicted by the model; positive residuals indicate higher-than-expected values, whereas negative residuals indicate lower-than-expected values. For each response variable, both bivariate and multiple-predictor (analysis-of-covariance-type) models were fitted. In OLS, continuous predictors were tested using *t* tests on regression slopes, and categorical factors (e.g., pinna presence) were evaluated using partial *F* tests comparing nested models. Corresponding PGLS models incorporated Pagel’s *λ*, estimated by maximum likelihood (ML) to account for phylogenetic covariance; factor effects were assessed using likelihood-ratio tests, and continuous predictors, using *t* tests on PGLS partial regression coefficients. Regression coefficients were estimated using restricted ML, with Pagel’s *λ* estimated by ML. Pagel’s *λ* quantifies the strength of phylogenetic signal in the model, with values close to zero indicating little phylogenetic structure in trait covariance rather than the absence of biological variation. Species relationships were reconstructed using available molecular phylogenies [46], with remaining taxa placed according to subfamily-level taxonomy, and branch lengths were standardized using Grafen’s method to generate a phylogenetic tree suitable for PGLS (Supplementary Material 1, Fig. S1). The low *λ* estimates should be interpreted cautiously because the phylogeny was assembled from published sources and branch lengths were standardized using Grafen’s method. Consequently, *λ* values primarily reflect relative phylogenetic structure within this dataset rather than precise evolutionary signal. The present dataset includes representatives from multiple major subfamilies but does not aim to encompass all recognized subfamilies of Tettigoniidae. Sampling was designed to capture variation in pinna configuration and morphology rather than to provide exhaustive phylogenetic coverage. For all regression models, *λ* indicates the phylogenetic signal; *R*^2^, the coefficient of determination; AICc, the corrected Akaike information criterion; *B* ± SE, regression slope ± standard error; *t*, the test statistic; and *P*, the probability value. The intercept represents the expected value of the response variable when all continuous predictors are zero (on the ln scale), and the categorical predictor is at its reference level; this does not correspond to a biologically meaningful scenario and is therefore not interpreted further. For interspecific comparative analyses (OLS and PGLS models), a single species-level value was used. In unilateral species, this corresponds to the mean of the pinna-covered and exposed tympana to maintain one independent datapoint per species. Side-specific differences were analyzed separately (Supplementary Material 1, Table S3). All analyses were conducted in R (v. 2025.05.1+513) using the packages ape, nlme, and MuMIn. Statistical results were interpreted cautiously, given the number of models evaluated across overlapping predictors. The *P* values are reported without formal correction for multiple comparisons and should therefore be considered in the context of effect sizes and overall model consistency.

## Results

### Body size, carrier frequency, and tympanal morphology

The relationships between body size, carrier frequency, and tympanal membrane morphology are presented in Fig. 1, while the corresponding statistical results are summarized in Table 2. Together, these analyses illustrate how variation in pronotum length, used here as a proxy for body size, relates to acoustic traits and tympanal features across the 18 bush-cricket species examined.

**Table 2.** Phylogenetically controlled and nonphylogenetically controlled regression analyses of bush-cricket acoustic and morphological traits. Results of phylogenetic generalized least squares (PGLS) and ordinary least squares (OLS) models examining relationships between carrier frequency, pronotum length (proxy for body size), and tympanal membrane properties (surface area and thickness) across 18 bush-cricket species.

Parameter	Phylogenetically controlled						Not phylogenetically controlled		
	λ	$R^{2}$	AICc	B±SE	t	P	B±SE	t	P
Carrier frequency ~ pronotum length	0.203	0.212	41.49	−0.995 ± 0.454	−2.191	0.044	−0.994 ± 0.479	−2.074	0.055
Tympanal properties ~ pronotum length | carrier frequency									
Tympanal surface area	0.242	0.495	25.72	1.179 ± 0.308	3.831	0.002	1.145 ± 0.325	3.519	0.003
Tympanal thickness	0.126	0.199	20.43	0.031 ± 0.270	0.114	0.911	−0.017 ± 0.276	−0.061	0.952
Tympanal properties ~ carrier frequency | pronotum length									
Tympanal surface area	0.242	0.495	25.72	0.086 ± 0.149	0.576	0.573	0.026 ± 0.151	0.173	0.865
Tympanal thickness	0.126	0.199	20.43	−0.184 ± 0.129	−1.431	0.173	−0.224 ± 0.128	−1.753	0.100

AICc, corrected Akaike information criterion; SE, standard error

**Fig. 2.**
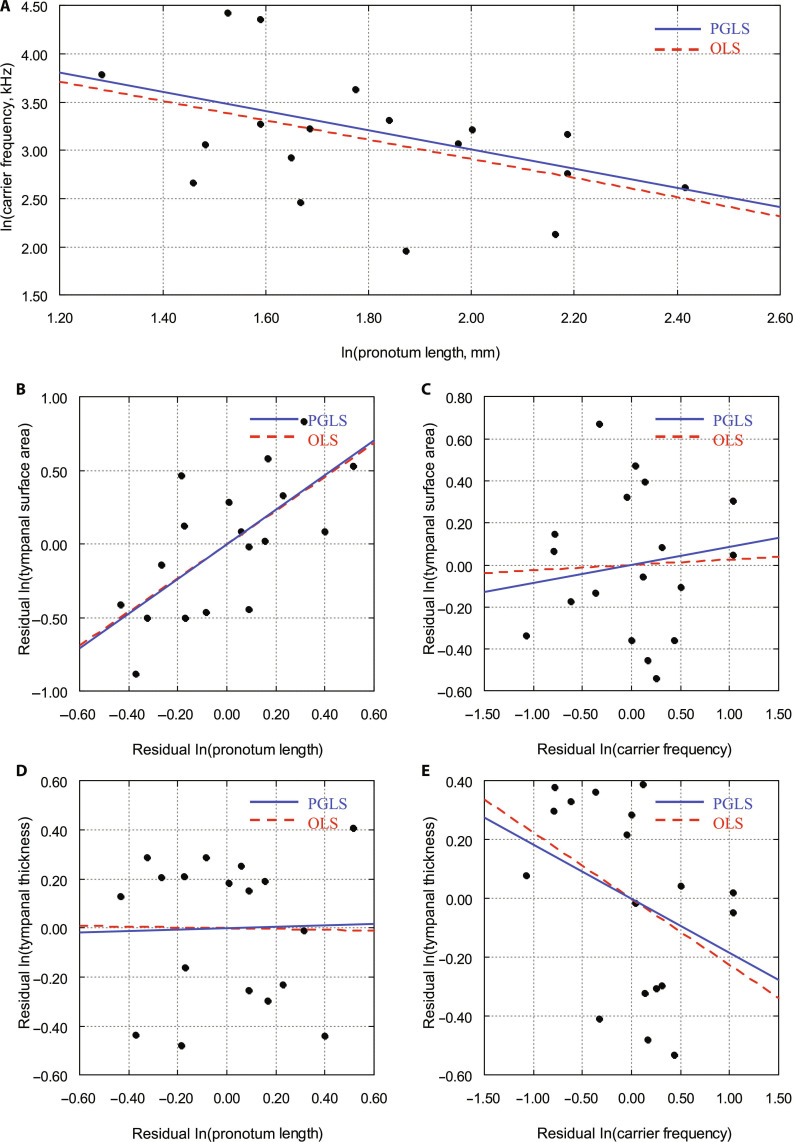
Comparative relationships between pronotum length, carrier frequency, and tympanal morphology in 18 species of bush-crickets. (A) Phylogenetic generalized least squares (PGLS, solid blue line) and ordinary least squares (OLS, dashed red line) regressions of ln-transformed carrier frequency (kHz) against ln-transformed pronotum length (mm; used here as a proxy for body size). (B and C) Standardized residuals of ln-transformed pronotum length and carrier frequency plotted against residual ln-transformed tympanal membrane surface area. (D and E) Standardized residuals of pronotum length and carrier frequency plotted against residual ln-transformed tympanal membrane thickness.

Carrier frequency decreased with increasing pronotum length (Fig. 2A), and this relationship was significant in the phylogenetically informed model (PGLS: *B* = −0.995 ± 0.454, 95% confidence interval [CI] [−1.88, −0.11], *P* = 0.044; *λ* = 0.20). In a multiple regression model including carrier frequency as a covariate, tympanal membrane surface area was positively related to pronotum length (PGLS: *B* = 1.18 ± 0.31, 95% CI [0.58, 1.78], *P* = 0.002; Fig. 2B). In a model including pronotum length as a covariate, tympanal surface area was not significantly related to carrier frequency (PGLS: *P* = 0.573; Fig. 2C). Tympanal membrane thickness showed no significant association with pronotum length when carrier frequency was included as a covariate (PGLS: *P* = 0.911; Fig. 2D). Tympanal membrane thickness was negatively correlated with carrier frequency (Fig. 2E), although this trend was not statistically significant (PGLS: *P* = 0.173). Phylogenetic signal was low for carrier frequency (*λ* = 0.20), moderate for tympanal membrane surface area (*λ* = 0.24), and low for tympanal membrane thickness (*λ* = 0.13). OLS analyses yielded comparable patterns of association (Table 2). To provide additional taxonomic context and a traditional view of the data distribution, subfamily-labeled residual plots and raw ln-transformed bivariate plots are provided in Supplementary Material 1, Figs. S2 to S10.

### Tympanal morphology and pinna resonance frequency

**Fig. 3.**
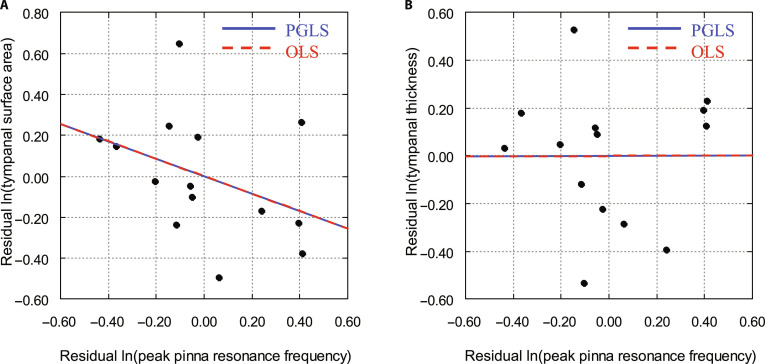
Relationships between tympanal morphology and peak pinna resonance frequency in bush-crickets with pinna cavities, controlling for pronotum length and carrier frequency. Standardized residuals of ln-transformed peak pinnal resonance frequency plotted against residual ln-transformed tympanal membrane surface area (A) and thickness (B) across 14 species with pinnal cavities (unilateral and bilateral). Phylogenetic generalized least squares (PGLS, solid blue line) and ordinary least squares (OLS, dashed red line) regressions are shown.

Tympanal membrane morphology was examined in relation to peak pinna resonance frequency while controlling for pronotum length and carrier frequency in the 14 species with well-developed pinna cavities (Fig. 3 and Table 3). Neither tympanal membrane surface area (PGLS: *P* = 0.219; Fig. 3A) nor thickness (PGLS: *P* = 0.990; Fig. 3B) showed significant associations with resonance frequency. Phylogenetic signal was absent in both models (*λ* = 0.00).

**Table 3.** Regression analyses (both PGLS and OLS) of tympanal properties with peak pinna resonance frequency in bush-crickets with pinna cavities, controlling for pronotum length and carrier frequency

Tympanal properties ~ peak pinna resonance frequency | pronotum length and carrier frequency	Phylogenetically controlled						Not phylogenetically controlled		
	λ	$R^{2}$	AICc	B±SE	t	*P*	B±SE	t	*P*
Tympanal surface area	0.0	0.391	13.28	−0.423 ± 0.322	−1.313	0.219	−0.423 ± 0.322	−1.313	0.219
Tympanal thickness	0.0	0.138	13.15	0.004 ± 0.325	0.013	0.990	0.004 ± 0.325	0.013	0.990

PGLS, phylogenetic generalized least squares; OLS, ordinary least squares; AICc, corrected Akaike information criterion; SE, standard error

### Association of pinna type with tympanal morphology

**Fig. 4.**
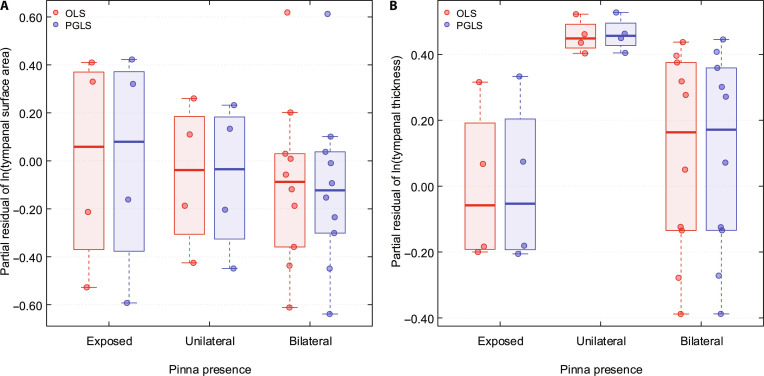
Association between pinna presence and tympanal morphology in bush-crickets, controlling for pronotum length and carrier frequency. Box-and-whisker plots showing variation in tympanal membrane surface area (A) and thickness (B) across species with exposed, unilateral, and bilateral pinnae configurations. Phylogenetic generalized least squares (PGLS; blue) and ordinary least squares (OLS; red) estimates are shown.

Tympanal membrane morphology was compared across the 18 species differing in pinna condition (exposed, unilateral, and bilateral) while controlling for pronotum length and carrier frequency (Fig. 4 and Table 4). Tympanal membrane surface area did not differ significantly based on pinna presence (PGLS: *P* = 0.617 for unilateral and *P* = 0.685 for bilateral; Fig. 4A). Comparable results were obtained under OLS.

**Table 4.** Regression analyses (both PGLS and OLS) of tympanal membrane surface area and thickness in relation to pinna presence, controlling for pronotum length and carrier frequency

Parameter	Phylogenetically controlled						Not phylogenetically controlled		
	λ	$R^{2}$	AICc	B±SE	t	P	B±SE	t	P
Tympanal surface area ~ pronotum length + carrier frequency + pinna presence									
Intercept	0.300	0.495	29.98	11.721 ± 1.025	11.440	<0.001	11.859 ± 1.099	10.790	<0.001
Pronotum length				1.135 ± 0.350	3.240	0.006	1.146 ± 0.386	2.970	0.011
Carrier frequency				0.092 ± 0.161	0.570	0.579	0.037 ± 0.171	0.220	0.832
Pinna presence: unilateral				−0.139 ± 0.272	−0.510	0.617	−0.061 ± 0.296	−0.200	0.841
Pinna presence: bilateral				−0.094 ± 0.228	−0.410	0.685	−0.091 ± 0.234	−0.390	0.703
Tympanal thickness ~ pronotum length + carrier frequency + pinna presence									
Intercept	0.000	0.427	19.23	3.042 ± 0.795	3.830	0.002	3.042 ± 0.795	3.830	0.002
Pronotum length				0.250 ± 0.279	0.900	0.386	0.250 ± 0.279	0.900	0.386
Carrier frequency				−0.154 ± 0.123	−1.250	0.233	−0.154 ± 0.123	−1.250	0.233
Pinna presence: unilateral				0.456 ± 0.2142	2.130	0.053	0.456 ± 0.2142	2.130	0.053
Pinna presence: bilateral				0.093 ± 0.169	0.550	0.592	0.093 ± 0.169	0.550	0.592

PGLS, phylogenetic generalized least squares; OLS, ordinary least squares; AICc, corrected Akaike information criterion; SE, standard error

However, tympanal membrane thickness varied with pinna condition, with unilateral pinnae showing a tendency toward thicker tympana compared with species lacking pinnae (PGLS: *B* = 0.46 ± 0.21, 95% CI [0.04, 0.88], *P* = 0.053; Fig. 4B and Table 4). OLS yielded the same pattern. No significant relationship was detected for bilateral pinnae (PGLS: *P* = 0.592; Fig. 4B). Phylogenetic signal was moderate for surface area (*λ* = 0.300) and absent for thickness (*λ* = 0.000). When *λ* is estimated as zero or close to zero, there is no detectable phylogenetic structure in the data. In this case, the PGLS model effectively reduces to an OLS model, resulting in identical parameter estimates.

### Asymmetries in the tympanal morphology of unilateral-pinna species

**Fig. 5.**
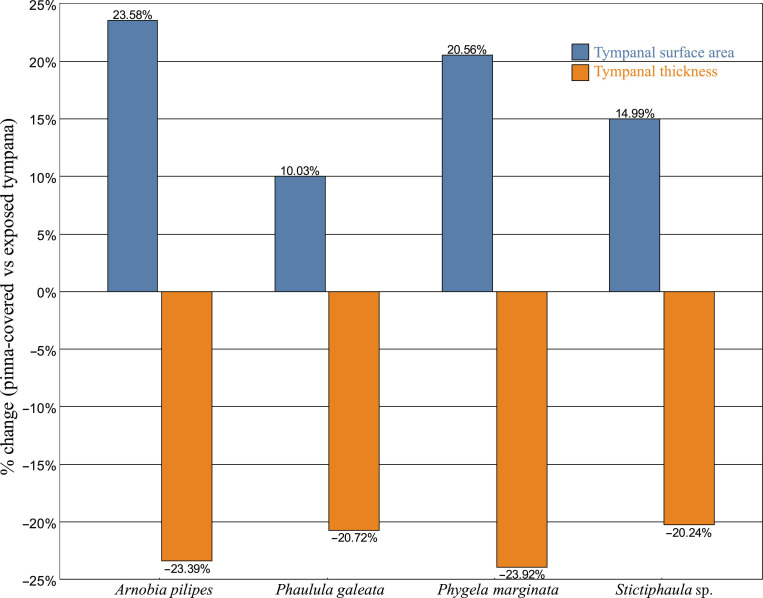
Within-species comparisons of tympanal properties between pinna-covered and exposed ears in bush-crickets with unilateral pinna. Percentage difference in tympanal membrane surface area (blue bars) and thickness (orange bars) between pinna-covered and exposed tympanum for 4 species with unilateral pinna (*Arnobia pilipes*, *Phaulula galeata*, *Phygela marginata*, and *Stictophaula* sp.). Positive values indicate a larger or thicker tympanum under the pinna relative to the exposed side, and negative values indicate a smaller or thinner tympanum under the pinna.

To address potential asymmetry in unilateral Phaneropterinae taxa, the pinna-covered and exposed tympana were analyzed separately. These within-species comparisons revealed consistent morphological differences between the 2 sides (Fig. 5; Supplementary Material S1, Table S3). Tympanal membrane surface area was generally larger on the pinna-covered side, ranging from +10.03% (*Phaulula galeata*) up to +23.58% (*Arnobia pilipes*). In contrast, tympanal membrane thickness was generally reduced on the pinna-covered side, ranging from −20.24% (*Phygela marginata*) down to −23.92% (*Stictophaula* sp.). These differences were supported by paired tests across unilateral taxa (surface area: paired *t* test, *t* = 3.15, *P* = 0.035; thickness: paired *t* test, *t* = −3.97, *P* = 0.017).

## Discussion

### Relationship between body size, carrier frequency, and tympanal morphology

This comparative study provides new insights into the relationships between pronotum length (as a proxy for body size), carrier frequency, and tympanal morphology in bush-crickets while also testing the potential associations between auditory pinnae and tympanal structure. Across the 18 species examined, pronotum length was significantly associated with carrier frequency, with larger species producing lower-frequency calls. This relationship persisted after controlling for phylogeny and matches previously reported body size–frequency scaling in bush-crickets [47–50].

Pronotum length also predicted tympanal membrane surface area but not thickness. Larger species possessed proportionally larger tympanal membranes, suggesting that ear morphology shows scaling relationships with body size across species. In contrast, tympanal thickness was unrelated to size, implying that this trait may vary independently of body size, perhaps linked more directly to tuning or mechanical protection of the membrane. When controlling for size, tympanal surface area showed no significant association with carrier frequency, whereas tympanal thickness exhibited a weak, nonsignificant negative relationship with carrier frequency. This nonsignificant trend likely arises because some of the most ultrasonic species in our dataset, such as *Arachnoscelis* sp. and *Haenschiella* sp. (Supplementary Material 1, Table S1), do not exhibit the thinnest tympana. So, higher carrier (dominant) frequencies in these predominantly narrowband calls are not necessarily associated with reduced membrane thickness. Instead, tympanic membranes typically operate as broadly tuned structures that provide sensitivity across a wide acoustic range. The present data do not indicate a specific enhancement of responsiveness near the carrier frequency. Consequently, other components of the auditory system, including the acoustic trachea, bulla, and pinnal cavity geometry, may contribute to shaping auditory sensitivity beyond tympanal morphology alone. As the present study did not include direct measurements of hearing sensitivity, functional interpretations remain inferential.

### Pinnal resonance and tympanal morphology

In species with pinnal cavities, neither tympanal surface area nor thickness correlated with peak pinna resonance frequency. These results indicate that tympanal morphology and cavity resonance are not tightly coupled, with resonance properties appearing more closely associated with cavity geometry than with membrane dimensions. Phylogenetic signal was absent in these models, suggesting that these traits may be evolutionarily variable and shaped by ecological rather than phylogenetic constraints. Notably, peak pinna resonance frequencies were consistently higher than species-specific carrier frequencies and therefore do not align with conspecific calling signals. Instead, resonance peaks fall within the ultrasonic range commonly associated with bat echolocation. As this study did not include direct measurements of auditory sensitivity (e.g., neurophysiology or in vivo vibrometry), functional interpretations regarding hearing range remain inferential; however, pinnal resonance is consistent with a potential role in ultrasonic predator detection rather than in conspecific communication. Scaled 3D-printed resonance measurements provided an independent estimate of cavity tuning properties, enabling tests of whether pinnal acoustic gain covaries with tympanal membrane morphology across species. The lack of a significant association supports the interpretation that cavity resonance primarily reflects cavity geometry rather than tympanal structural scaling.

### Pinna configuration and asymmetry in unilateral taxa

The presence of pinnae did not show significant association tympanal surface area across the broader dataset, but tympanal thickness did vary with pinna configuration. Species with unilateral pinnae had, on average, thicker tympanal membranes than species lacking pinnae, whereas bilateral pinnae showed no detectable relationship with tympanal dimensions. All unilateral taxa in this study belonged to the Phaneropterinae subfamily, so this pattern could, in principle, reflect clade-specific morphology. However, the association between unilateral pinnae and thickness remained when phylogeny was incorporated via PGLS (*λ* = 0), suggesting that this pattern is not solely attributable to shared ancestry within this group. One interpretation is that tympanal dimensions are constrained within a functional range: membranes must remain sufficiently thin and compliant to detect sound effectively, yet robust enough to resist mechanical stress. In species with unilateral pinnae, one possible explanation is that the presence of a cavity on only one side may modify local mechanical constraints, potentially contributing to differences in membrane thickness. Consequently, this finding does not strongly support the hypothesis that auditory pinnae primarily evolved for the mechanical protection of tympanal membranes [19–21], instead supporting recent work emphasizing their role in acoustic gain and predator detection [22,23].

Within-species comparisons in Phaneropterinae taxa with unilateral pinnae revealed consistent morphological asymmetries between pinna-covered and exposed tympana. Despite unilateral species having thicker tympana overall at the interspecific level, pinna-covered tympana exhibited larger surface areas but thinner membranes than their exposed counterparts. These asymmetries suggest that the presence of a pinna is linked to differences in tympanal morphology or mechanical properties in a localized manner. A larger surface area may enhance sound capture under the pinna, whereas a reduced thickness could increase sensitivity to ultrasonic frequencies, potentially complementing the cavity’s acoustic gain [51,52].

Taken together, these findings demonstrate that tympanal morphology in bush-crickets arises from multiple interacting factors. Tympanal surface area scales predictably with body size, reflecting biomechanical constraints on acoustic sensitivity, whereas thickness remains decoupled from size and shows greater variability across species. The absence of broad associations with pinna presence, together with consistent asymmetries in unilateral species, suggests that links between pinnae and tympanal morphology occur only in specific configurations rather than across all taxa. Thus, tympanal morphology reflects a complex interplay of scaling, ecological divergence, and structural innovation rather than simple covariation with carrier frequency.

### Limitations

An important limitation of this study is that tympanal morphology was quantified from a single adult male individual per species and from one foreleg per specimen. This approach was necessary due to the substantial computational demands of μCT segmentation for tympanal morphology extraction. Although each analyzed leg provided 2 tympana for surface area and thickness measurements, allowing basic internal consistency checks, the dataset does not capture the full extent of potential within-species variability. The comparative regressions nevertheless revealed consistent interspecific patterns within this dataset, although within-species variance could not be estimated, and residual variation in the models therefore reflects interspecific differences only. However, the absence of multiple individuals per species restricts our ability to quantify population-level variation explicitly. In addition, the phylogenetic tree was assembled from previously published molecular phylogenies, with some taxa placed at the subfamily level and branch lengths standardized using Grafen’s method to produce a fully resolved working tree rather than a time-calibrated phylogeny. Consequently, certain relationships remain partially unresolved (polytomies), which may influence estimates of phylogenetic signal. Although PGLS results were broadly consistent with OLS models, analyses incorporating fully resolved phylogenies would further refine these comparative inferences. Future studies incorporating larger sample sizes per species and broader phylogenetic sampling across additional subfamilies would enable a more precise assessment of intraspecific variability and developmental variation in tympanal morphology.

## Conclusion

In summary, this study provides the first comparative analysis of tympanal morphology across bush-cricket species, integrating morphological, acoustic, and phylogenetic variables. This comparative analysis suggests that bush-cricket tympanal morphology varies with multiple structural and acoustic traits. Scaling relationships were evident, with tympanal surface area increasing predictably with body size, which may help maintain acoustic sensitivity across species of different sizes, while thickness remained decoupled from size and acoustic variables. The role of the auditory pinnae was more nuanced. While pinnae were not associated with tympanal surface area across species, unilateral pinnae were associated with thicker tympana, whereas bilateral pinnae showed no difference. This suggests that pinna presence alone does not consistently predict tympanal morphology and does not clearly support a purely protective origin of pinnae. However, within-species comparisons in taxa with unilateral pinnae revealed consistent asymmetries: pinna-covered tympana were larger in surface area but thinner than exposed tympana. These localized patterns suggest that pinnae are associated with differences in tympanal morphology and mechanics that may complement their role in generating sound pressure gain.

The comparative patterns identified here provide a foundation for future work investigating how tympanal morphology interacts with other elements of the bush-cricket auditory system, such as tracheal pathways, cuticular mechanics, and neural processing. Incorporating wider taxonomic sampling, behavioral assays, and in vivo vibrational measurements will help clarify how morphological variation translates into functional differences in sensitivity and tuning. Beyond biological relevance, these findings may also inform future engineering design. The diversity of tympanal structures across species offers natural templates for bioinspired acoustic sensors, particularly in the ultrasonic range. The consistent scaling of tympanal surface area with body size and the localized asymmetries associated with pinnae may provide conceptual design principles for creating lightweight, frequency-tuned devices. Such approaches could potentially inform the development of miniature microphones for robotics, surveillance, or medical diagnostics, where sensitivity to specific frequency bands is critical.

## Data Availability

Data supporting the findings of this study are provided in the Supplementary Materials and are available upon request from the corresponding authors. A preprint version of this article is published in bioRxiv: https://doi.org/10.1101/2025.11.11.687687.
