# Supplementary material for: Comparative Analysis of Morphological and Acoustic Correlates of Bush-Cricket Tympanic Membranes
Source: Comput Struct Biotechnol J. 2026 Apr 9;35(1):0035. doi: 10.34133/csbj.0035 (PMC13082575; doi:10.34133/csbj.0035)
Supplement: Supplementary 1 — Supplementary Materials 1 and 2 Tables S1 to S3 Figs. S1 to S10 [file csbj.0035.f1.zip › Supplementary Material 1.pdf]

Table S1: Morphological and acoustic traits of bush-cricket species examined in this study. For each species, the table lists geographic location, subfamily, presence or absence of cuticular pinnae, carrier frequency of the call, pronotum length, mean tympanal membrane surface area, and mean tympanal membrane thickness. For own lab recordings, male calling songs were recorded inside an acoustic chamber. Individuals were suspended in a mesh cage from the chamber ceiling, and an ultrasound-sensitive 1/8″ microphone (6.5–140 kHz; Brüel & Kjær, Nærum, Denmark) was positioned approximately 30 cm from the insect and oriented directly towards it. The microphone was coupled to a Brüel & Kjær 2633 preamplifier and connected to a G.R.A.S. 12AA two-channel power module (GRAS Sound & Vibration, Denmark). Signals were acquired using Polytec PSV acquisition software via a PSV-500 internal data acquisition board (Polytec, Waldbronn, Germany). A high-pass filter was set at 1 kHz, and recordings were sampled at 100 kHz [1].

Species	Location	Subfamily	Pinnae type	Carrier frequency (kHz)	Pronotum length (mm)	Mean tympana surface area (μm^2)	Mean tympana thickness (μm)
Arachnoscelis sp.	South America	Meconematinae	Bilateral	83.3	4.6	1,169,955.93	21.32
Arnobia pilipes	Asia	Phaneropterinae	Unilateral	26.4 [2]	4.9	1,278,630.35	28.16
Balboana tibialis	South America	Pseudophyllinae	Bilateral	13.7 [3]	11.2	2,204,844.61	39.66
Chibchella nigrospecula	South America	Pseudophyllinae	Bilateral	25 [4]	7.4	1,592,153.93	18.39
Copiphora gorgonensis	South America	Conocephalinae	Bilateral	23.7 [5]	8.9	1,257,312.79	15.07
Elimaea signata	Asia	Phaneropterinae	Bilateral	14.3 [2]	4.3	854,592.34	29.75
Haenschiella sp.	South America	Pseudophyllinae	Bilateral	78.2	4.9	968,515.83	23.16
Leptoderes ornatipennis	Asia	Phaneropterinae	Exposed	8.4	8.7	1,474,803.50	35.56
Mecopoda elongata	Asia	Mecopodinae	Exposed	15.9	8.9	2,889,944.44	25.28
Moncheca elegans	South America	Conocephalinae	Bilateral	38	5.9	1,024,815.33	24.58
Phaulula galeata	Asia	Phaneropterinae	Unilateral	21.3 [2]	4.4	716,804.25	31.91
Phlugis poecilla	South America	Meconematinae	Exposed	44.2	3.6	416,243.96	13.17
Phygela marginata	Asia	Phaneropterinae	Unilateral	11.7 [2]	5.3	1,168,524.38	33.63
Phyllomimus detersus	Asia	Pseudophyllinae	Bilateral	7.1 [2]	6.5	906,920.30	26.01
Ragoniella pulchella	South America	Conocephalinae	Bilateral	27.6	6.3	714,463.38	17.58
Satizabalus jorgevargasi	South America	Pseudophyllinae	Bilateral	18.7 [1]	5.2	1,934,015.49	15.25
Stictophaula sp.	Asia	Phaneropterinae	Unilateral	25.1 [2]	5.4	718,740.64	30.82
Stilpnochlora sp.	South America	Phaneropterinae	Unilateral	21.5	7.2	2,115,788.91	17.8

[1] Holmes, L. B., Woodrow, C., Sarria, F. A., Celiker, E. and Montealegre-Z., F. (2024). Wing mechanics and acoustic communication of a new genus of sylvan katydid (Orthoptera: Tettigoniidae: Pseudophyllinae) from the Central Cordillera cloud forest of Colombia. *PeerJ* 12, e17501. doi:10.7717/peerj.17501.

[2] Holmes, L. B., Kai, M. T., Japri, R., Chung, A. Y. C., Salvador, J. A. G., Nuñez, O. M., Muhammad, A. A., Abdullah, N. A., Vu, T. T., Pham, T. H. and Montealegre-Z., F. (2025). Revisiting the calling songs of katydids (Orthoptera: Tettigonioidea) from the tropical forests of Southeast Asia. *Raffles Bulletin of Zoology* 73, 385–418. doi:10.26107/RBZ-2025-0026.

[3] ter Hofstede, H. M., Kalko, E. K. V. and Fullard, J. H. (2010). Auditory-based defence against gleaning bats in neotropical katydids (Orthoptera: Tettigoniidae). *Journal of Comparative Physiology A: Neuroethology, Sensory, Neural, and Behavioral Physiology* 196, 349–358. doi:10.1007/s00359-010-0518-4.

[4] Montealegre-Z., F. and Morris, G. K. (1999). Songs and systematics of some Tettigoniidae from Colombia and Ecuador I. Pseudophyllinae (Orthoptera). *Journal of Orthoptera Research* 8, 163–236.

[5] Montealegre-Z., F. and Postles, M. (2010). Resonant sound production in *Copiphora gorgonensis* (Tettigoniidae: Copiphorini), an endemic species from *Parque Nacional Natural Gorgona*, Colombia. *Journal of Orthoptera Research* 19, 347–355.

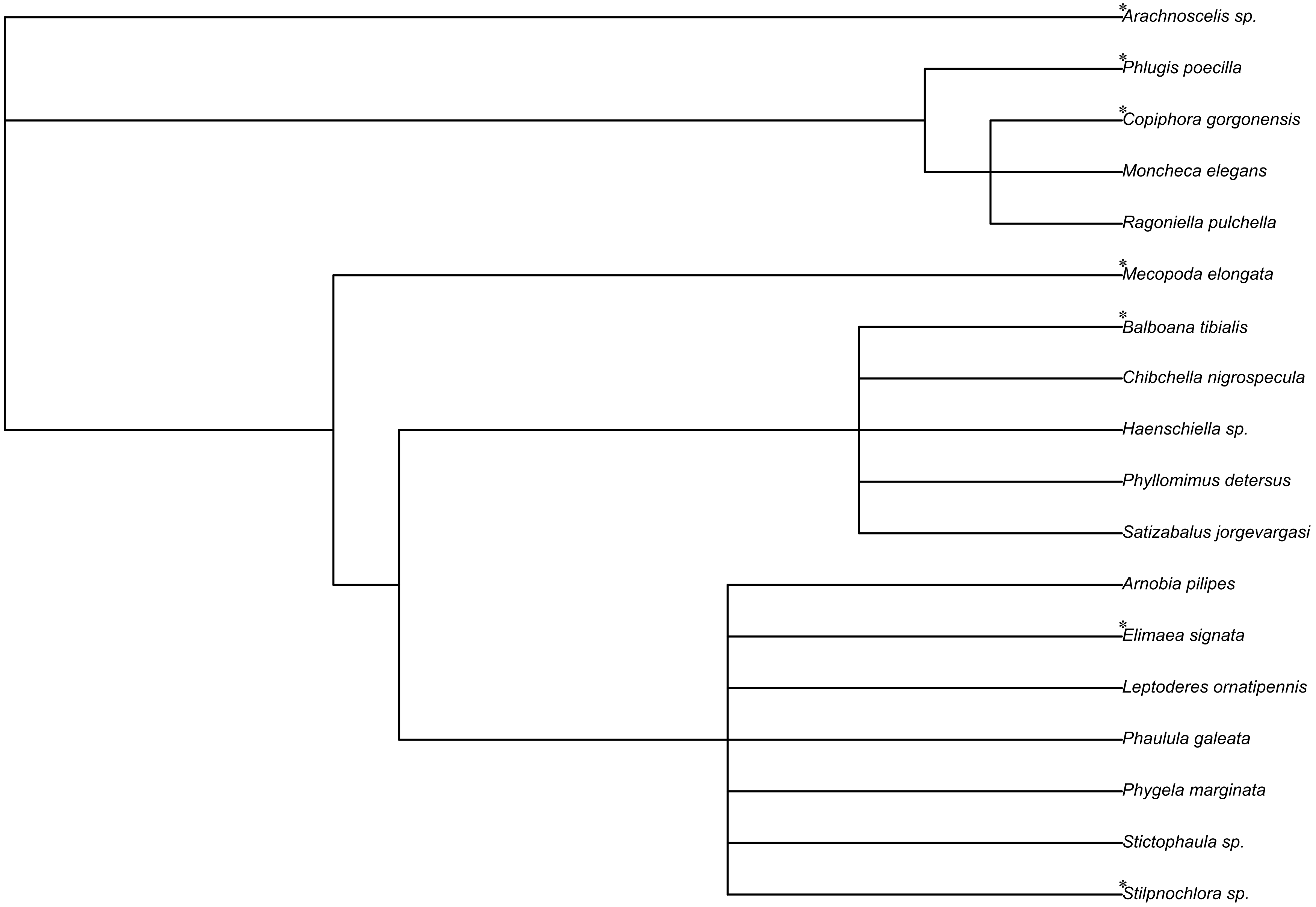

Fig. S1: Phylogenetic relationships of the bush-cricket species examined in this study. The tree illustrates the evolutionary placement of species for which morphological and acoustic traits were measured. Species marked with an asterisk (*) were placed in the tree based on available published phylogenetic hypotheses (see Methods). Species without an asterisk were not included in those source phylogenies and were therefore assigned according to current subfamily-level classification, being grouped within the respective reconstructed lineage. The phylogeny is presented to provide context for phylogeny-controlled comparative analyses of tympanal morphology with pronotum length and carrier frequency.

Table S2: Frequency of maximum gain at pinna resonance for bush-cricket species with pinna cavity.

Species	Frequency of max gain at pinna resonance (kHz)
Arachnoscelis sp.	50.63
Arnobia pilipes	140.63
Balboana tibialis	47.50
Chibchella nigrospecula	66.33
Copiphora gorgonensis	75.50
Elimaea signata	95.63
Haenschiella sp.	71.25
Moncheca elegans	71.48
Phaulula galeata	157.73
Phygela marginata	71.48
Phyllomimus detersus	86.25
Ragoniella pulchella	80.63
Satizabalus jorgevargasi	86.20
Stictophaula sp.	131.95
Stilpnochlora sp.	94.84

Table S3: Comparison of tympanal membrane morphology in Phaneropterinae bush-cricket species with exposed tympana on one side and pinna-covered tympana on the other side.

Species	Exposed tympana surface area (μm^2)	Pinnae-covered tympana surface area (μm^2)	Exposed tympana thickness (μm)	Pinna-covered tympana thickness (μm)
Arnobia pilipes	1,143,780.67	1,413,480.02	32.36	24.79
Phaulula galeata	682,585.70	751,022.79	35.71	28.31
Phygela marginata	1,059,591.20	1,277,457.55	38.67	29.42
Stictophaula sp.	668,614.36	768,866.92	34.43	27.46
Stilpnochlora sp.	2,094,999.82	2,136,578.00	17.88	17.74

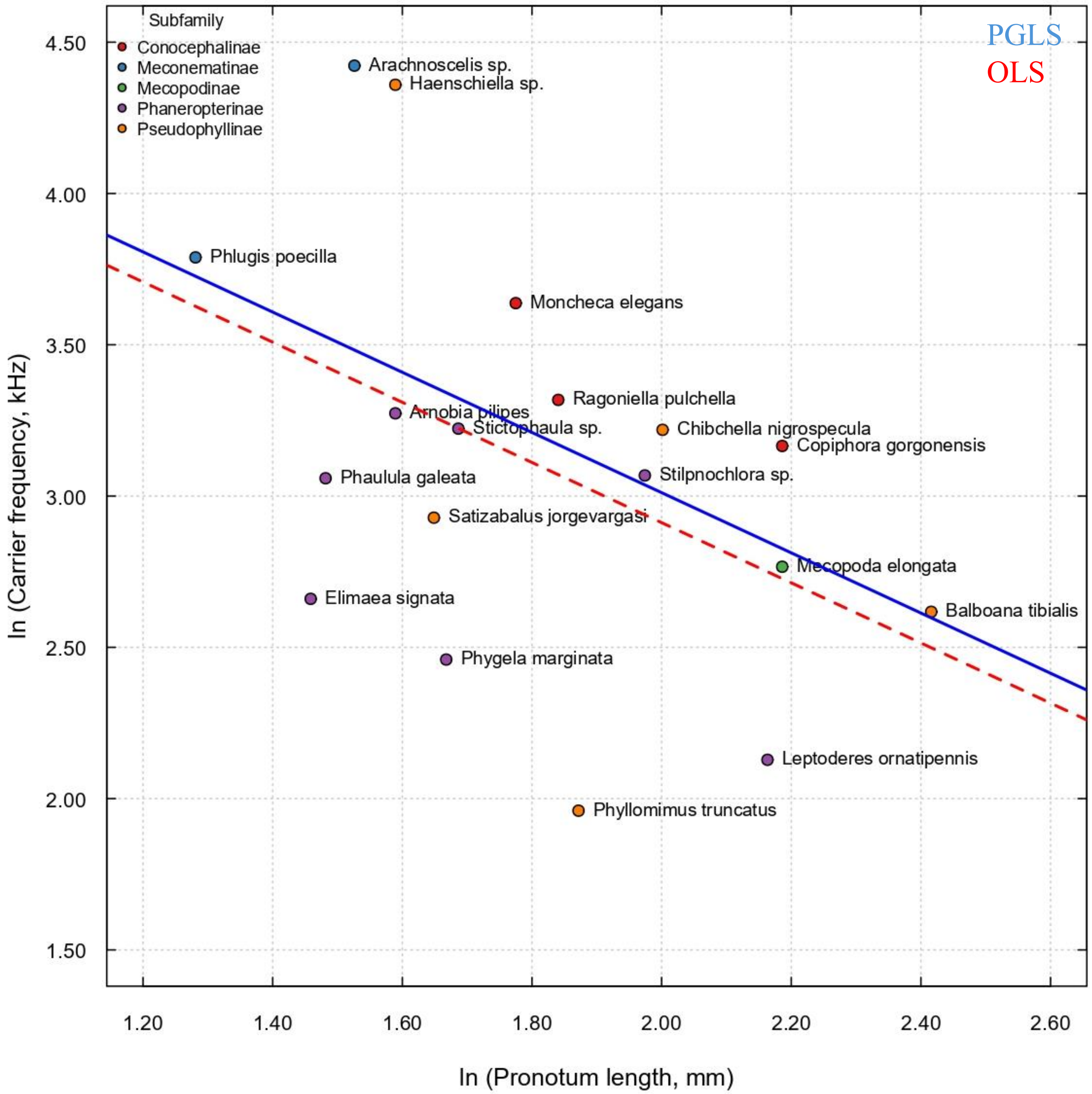

Fig. S2: Relationship between carrier frequency and pronotum length across 18 bush-cricket species. Points are color-coded by subfamily to illustrate taxonomic distribution. Solid blue and dashed red lines represent PGLS and OLS regression models, respectively.

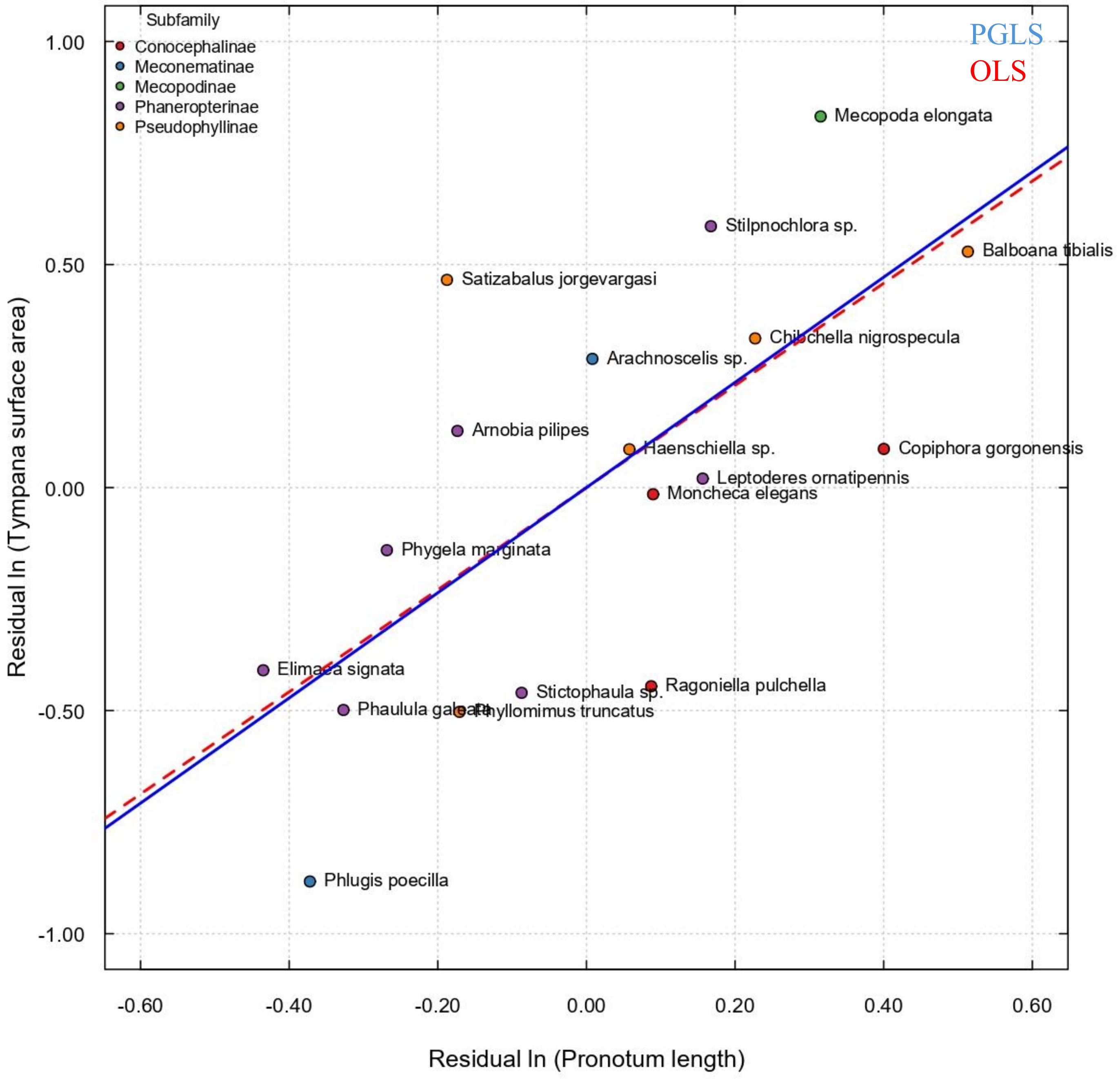

Fig. S3: Partial relationship between residual tympanal surface area and residual pronotum length, controlling for carrier frequency. Species are color-coded by subfamily. Residuals illustrate the association between body size and surface area independent of signal frequency.

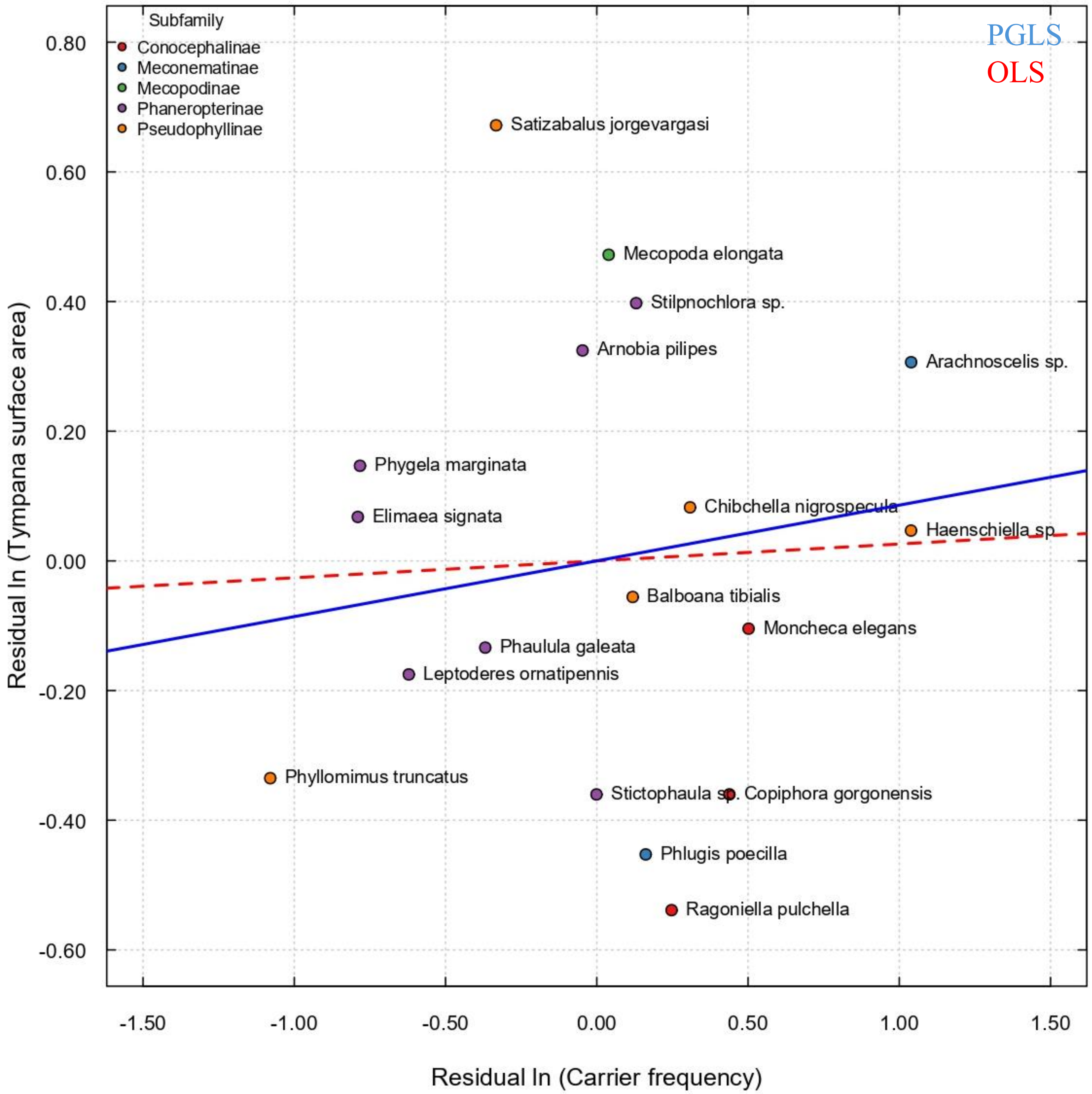

Fig. S4: Partial relationship between residual tympanal surface area and residual carrier frequency, controlling for pronotum length. Points are color-coded by subfamily. Residuals show the lack of a significant association between signal frequency and membrane surface area after accounting for body size.

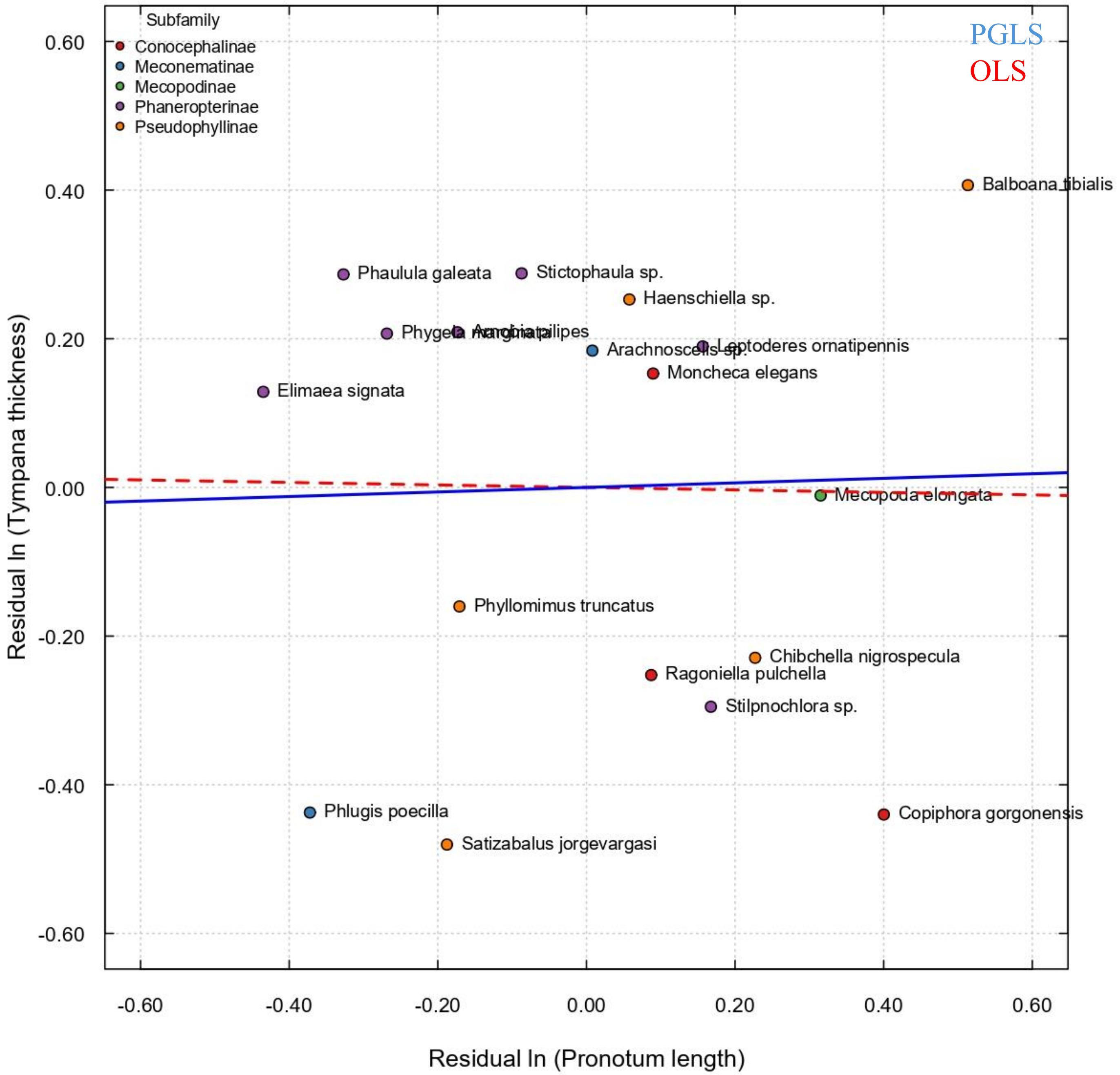

Fig. S5: Partial relationship between residual tympanal thickness and residual pronotum length, controlling for carrier frequency. Species are color-coded by subfamily. The plot illustrates that membrane thickness is largely independent of body size across the sampled taxa.

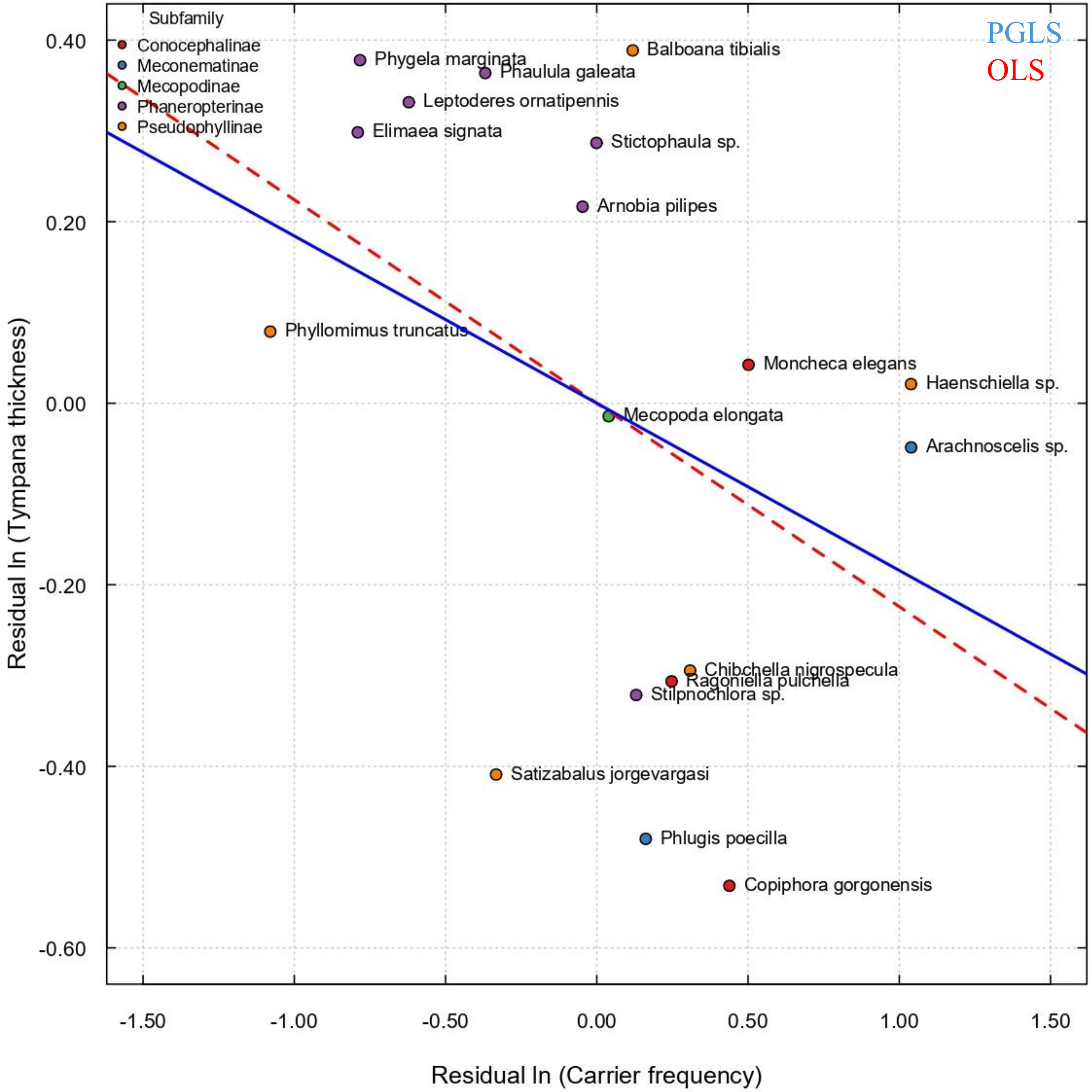

Fig. S6: Partial relationship between residual tympanal thickness and residual carrier frequency, controlling for pronotum length. Points are color-coded by subfamily. Residuals illustrate the non-significant negative trend between peak calling frequency and membrane thickness.

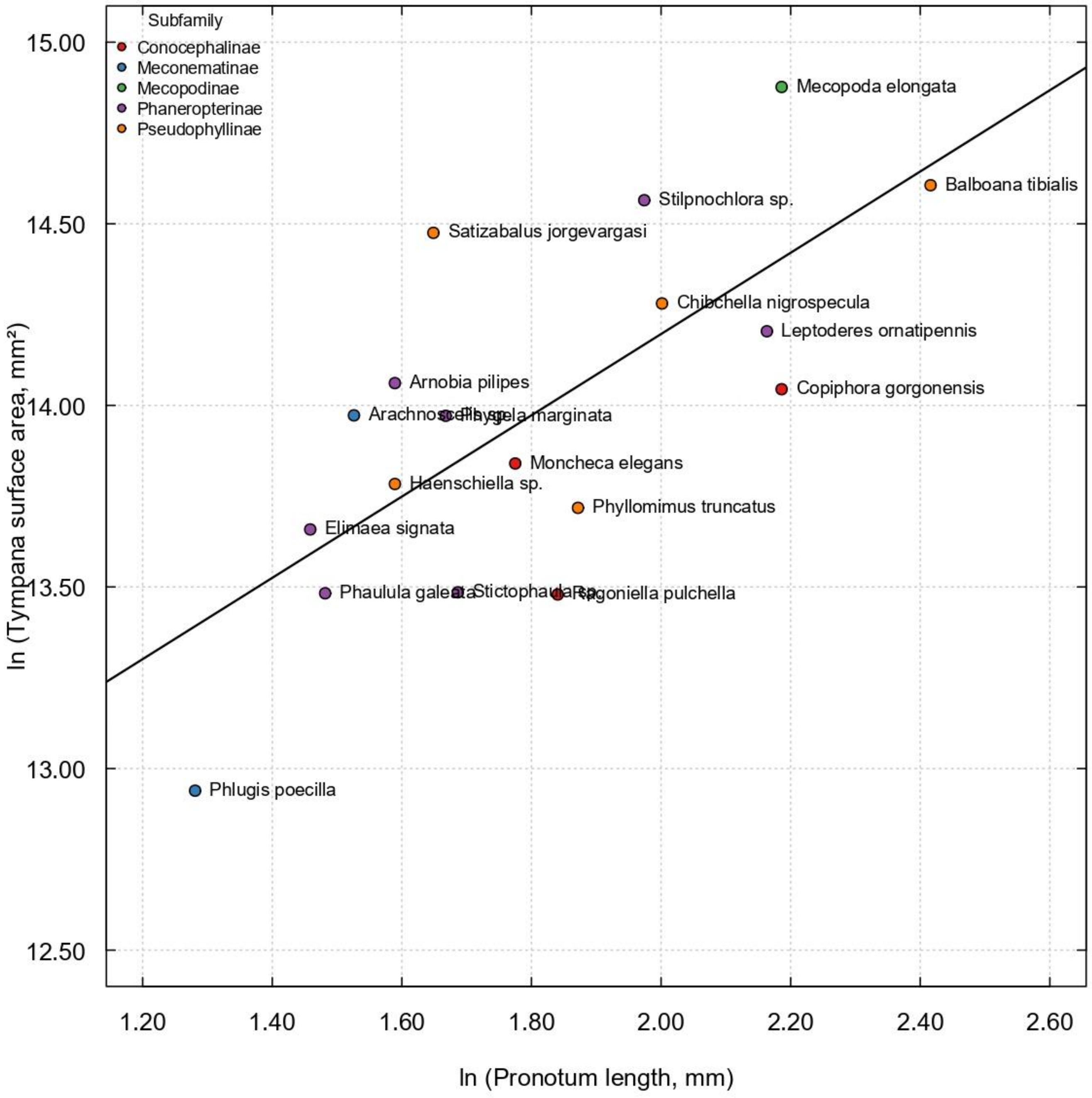

Fig. S7: Bivariate relationship between ln-transformed tympanal surface area and ln-transformed pronotum length. Raw data points are color-coded by subfamily to provide a traditional view of the allometric scaling between body size and ear morphology.

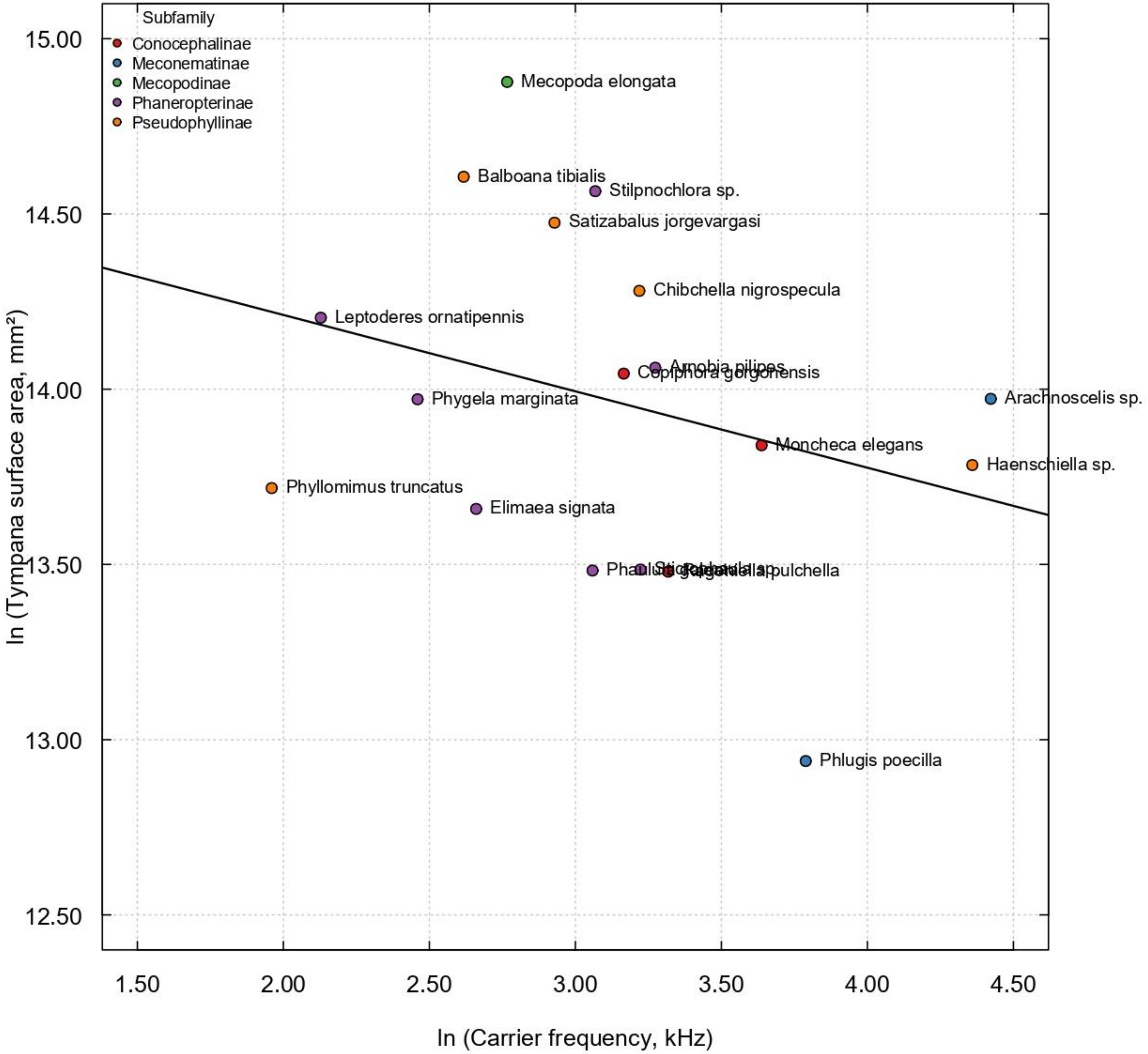

Fig. S8: Bivariate relationship between ln-transformed tympanal surface area and ln-transformed carrier frequency. The plot shows the distribution of raw species-level values, illustrating the overall relationship between signal frequency and membrane surface area.

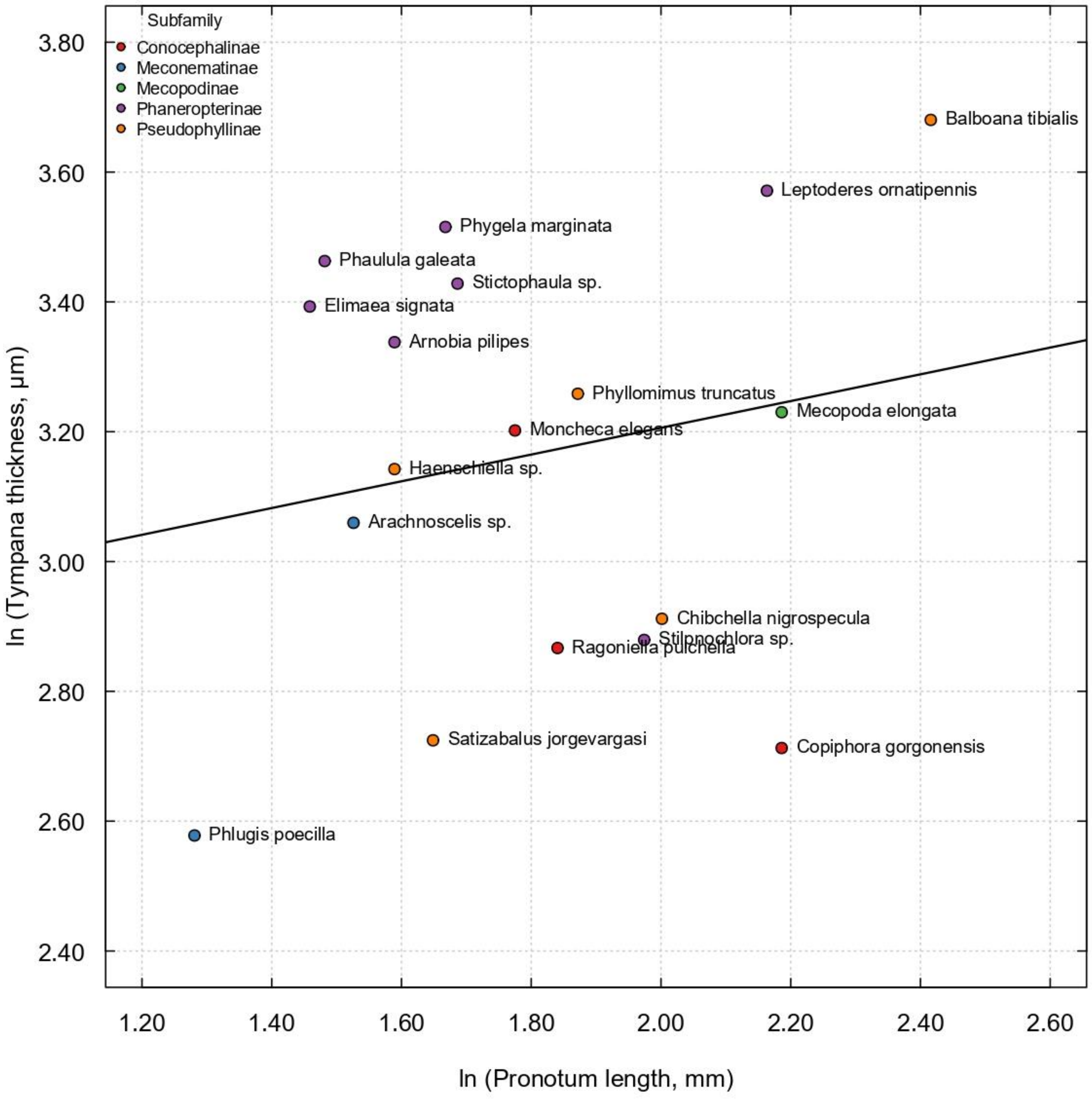

Fig. S9: Bivariate relationship between ln-transformed tympanal thickness and ln-transformed pronotum length. Points are color-coded by subfamily, showing the distribution of mean thickness values relative to body size.

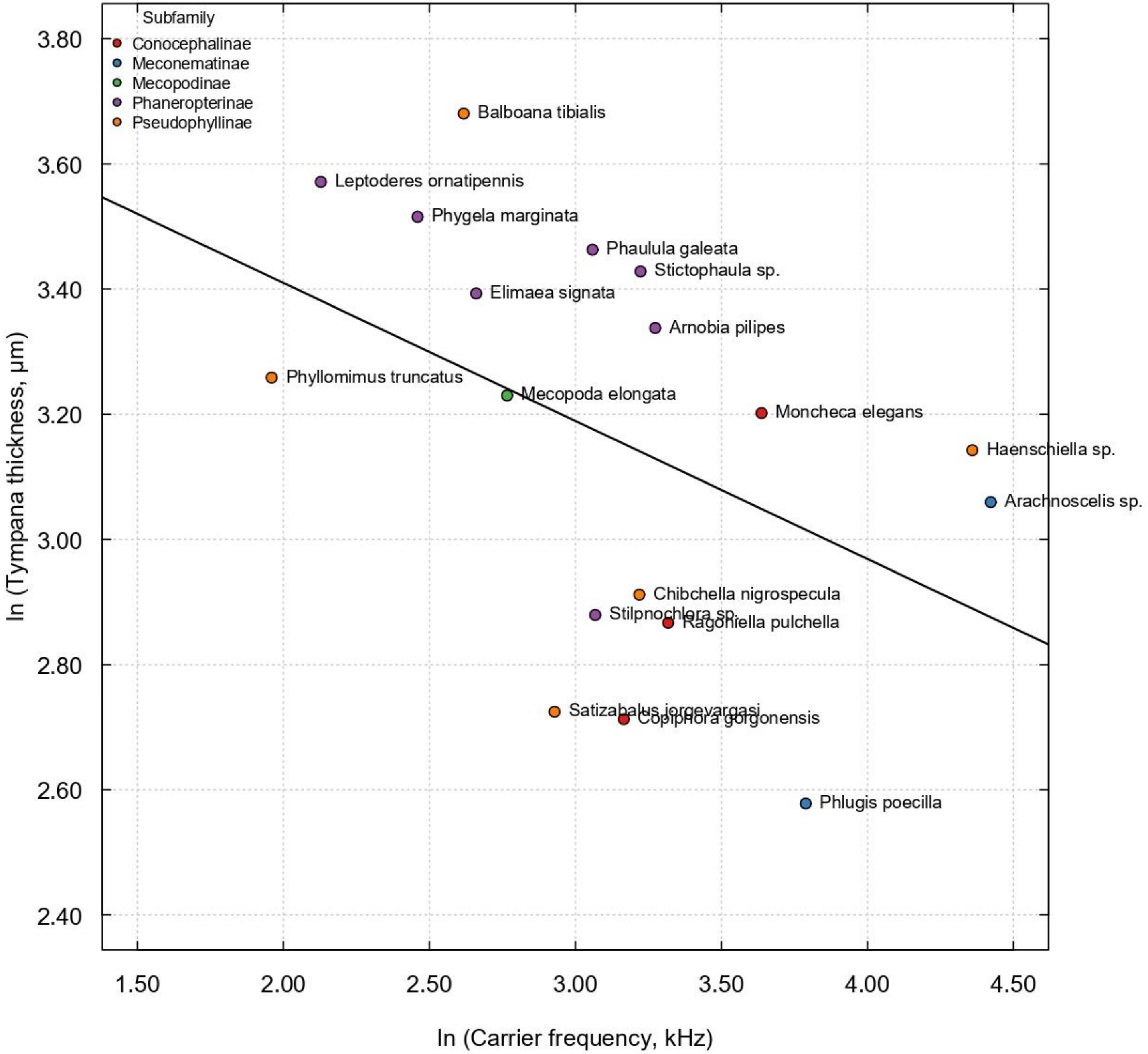

Fig. S10: Bivariate relationship between ln-transformed tympanal thickness and ln-transformed carrier frequency. Raw species-level values illustrate the trend between calling song frequency and membrane thickness across the 18 sampled species.
