# Supplementary material for: Comparative Analysis of Morphological and Acoustic Correlates of Bush-Cricket Tympanic Membranes
Source: Comput Struct Biotechnol J. 2026 Apr 9;35(1):0035. doi: 10.34133/csbj.0035 (PMC13082575; doi:10.34133/csbj.0035)

Arachnoscelis sp.

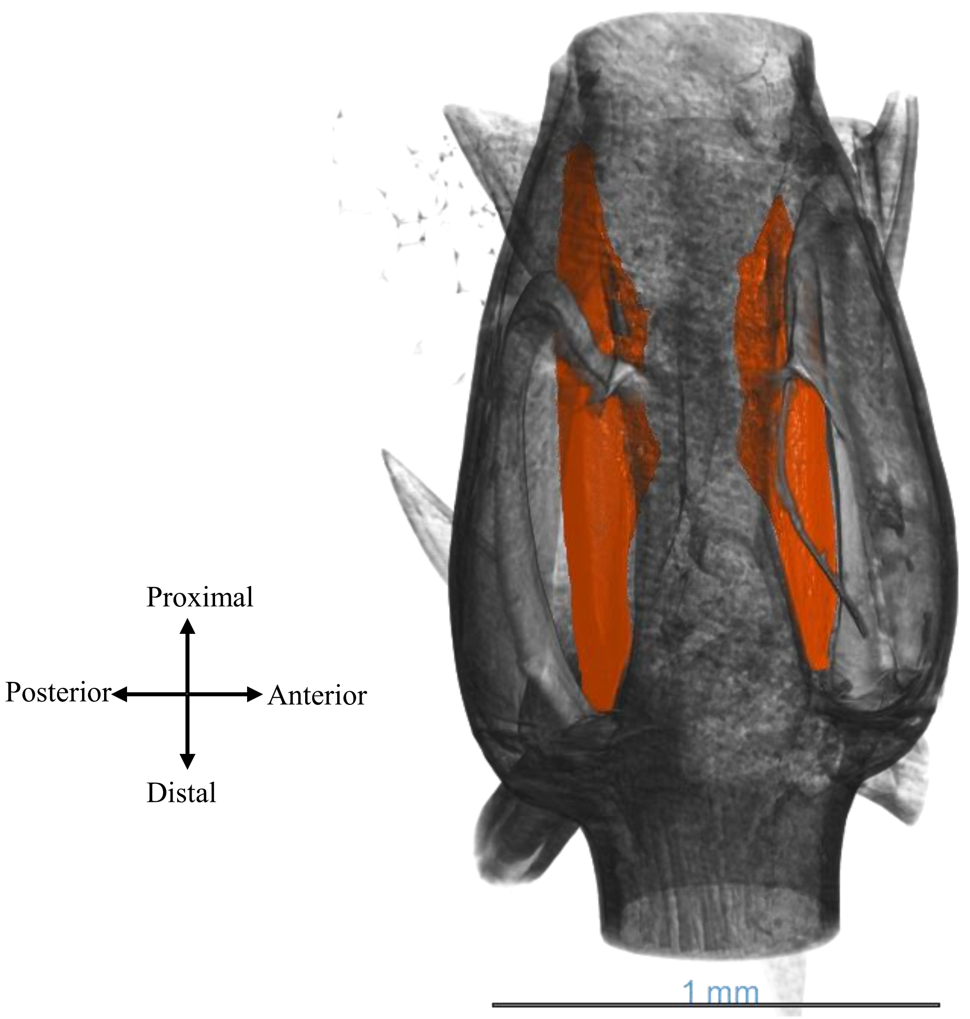

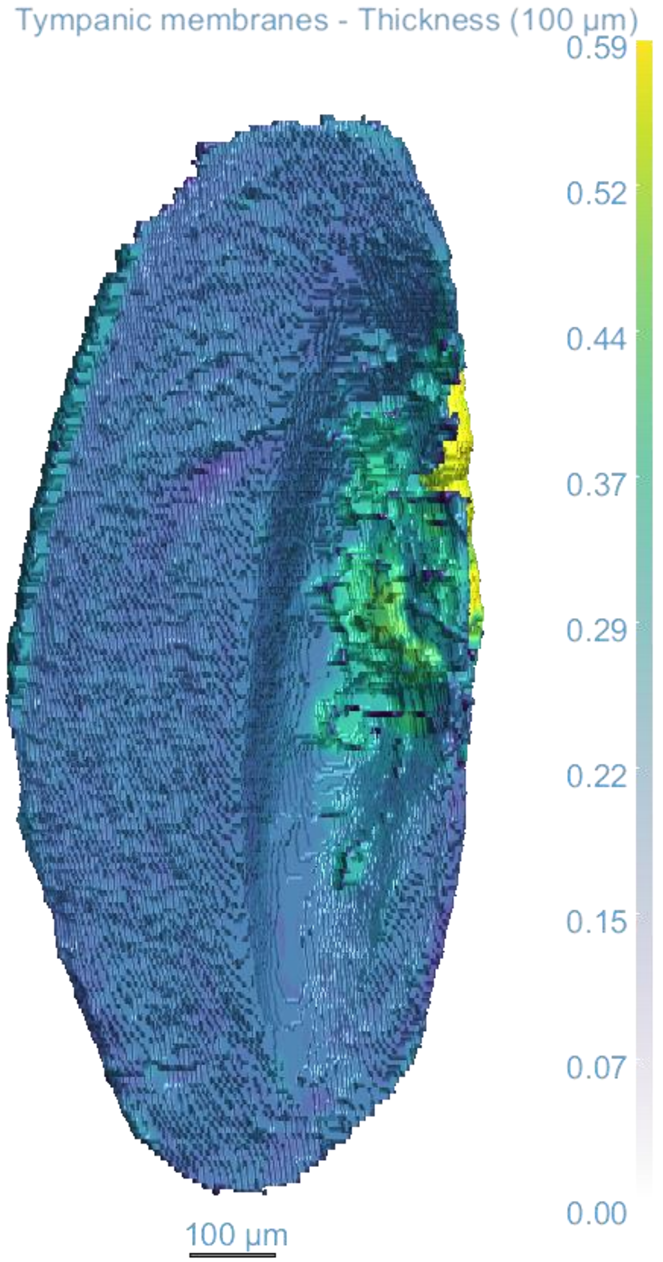

Arachnoscelis sp.

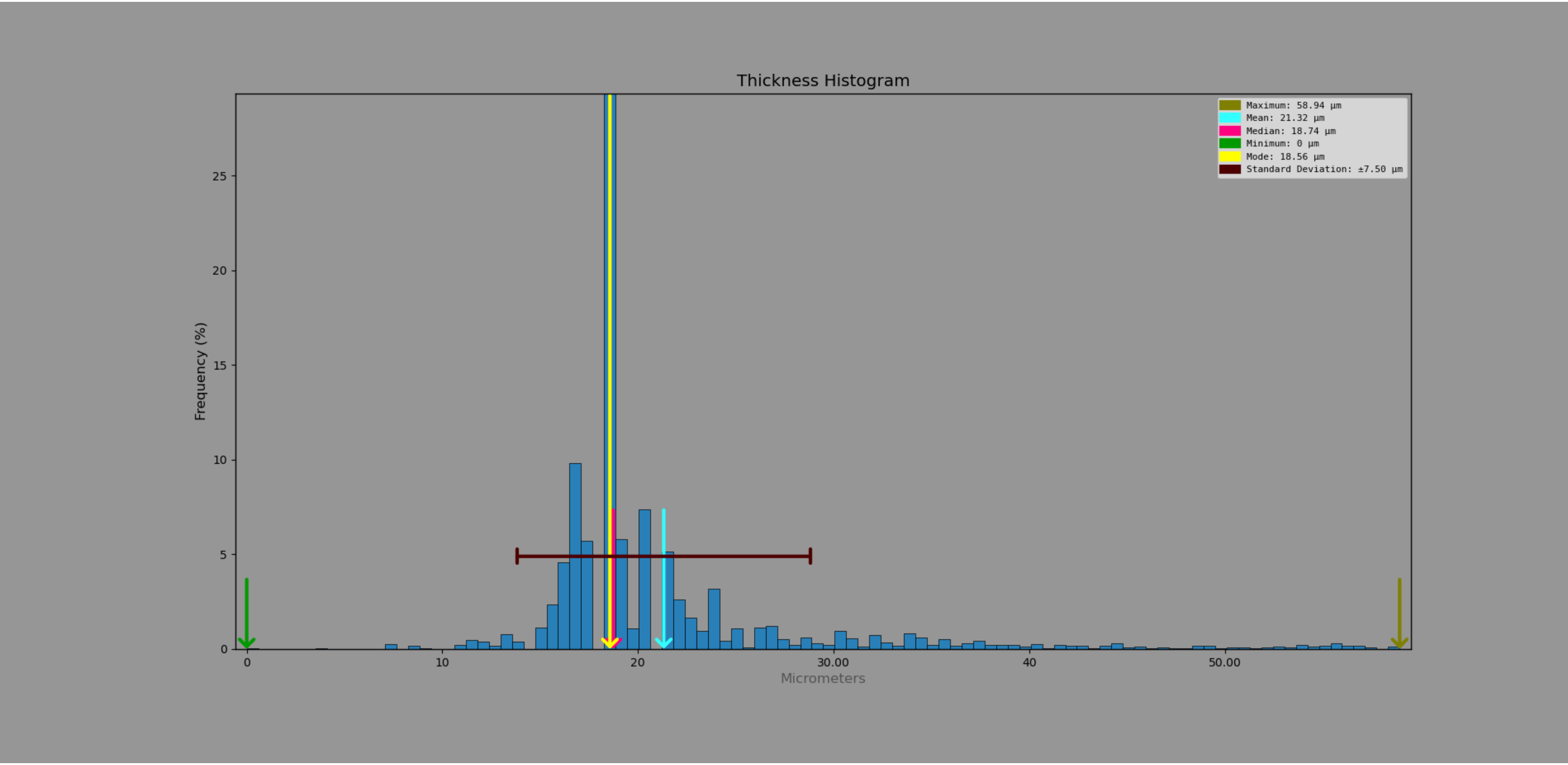

Arnobia pilipes

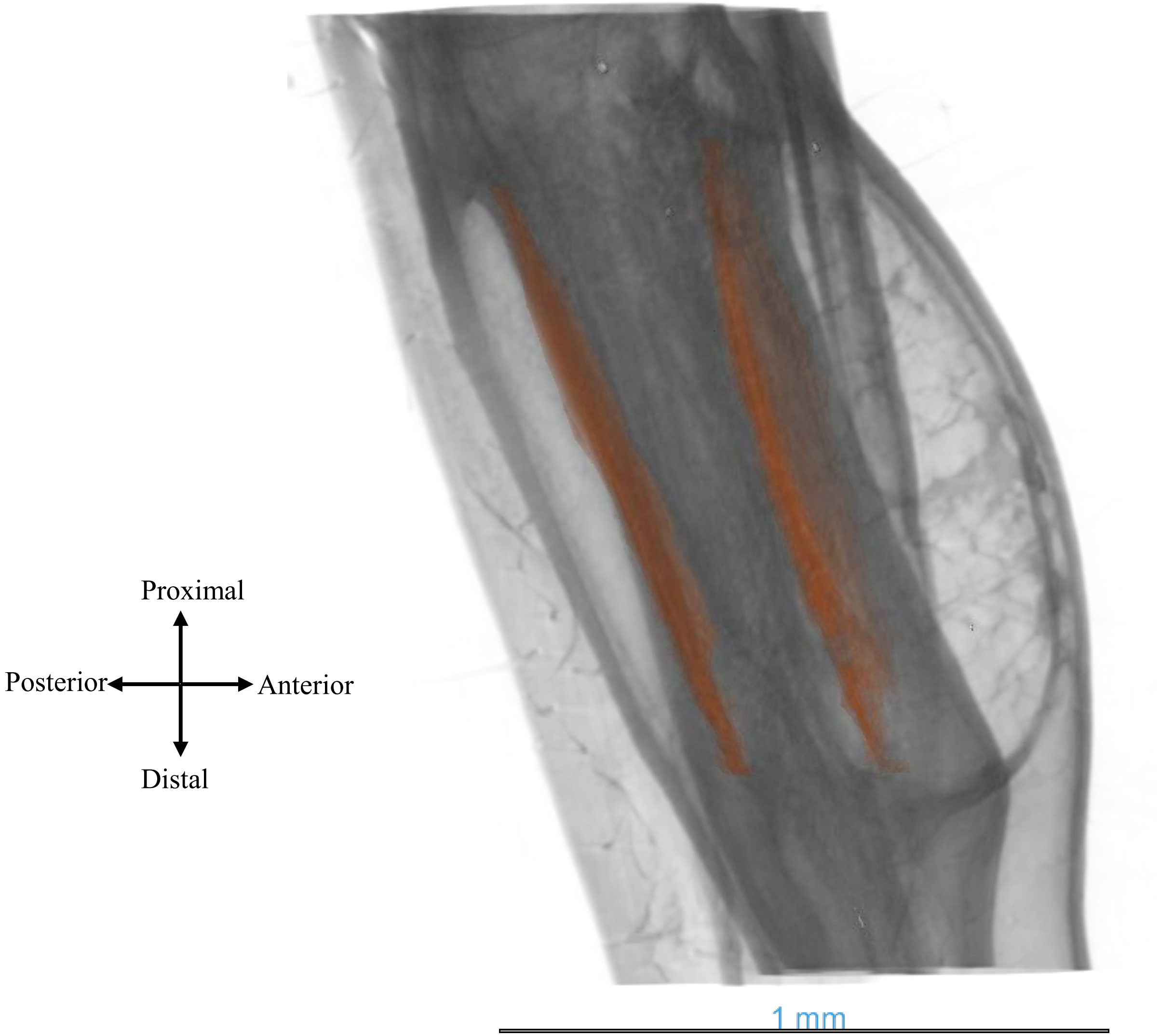

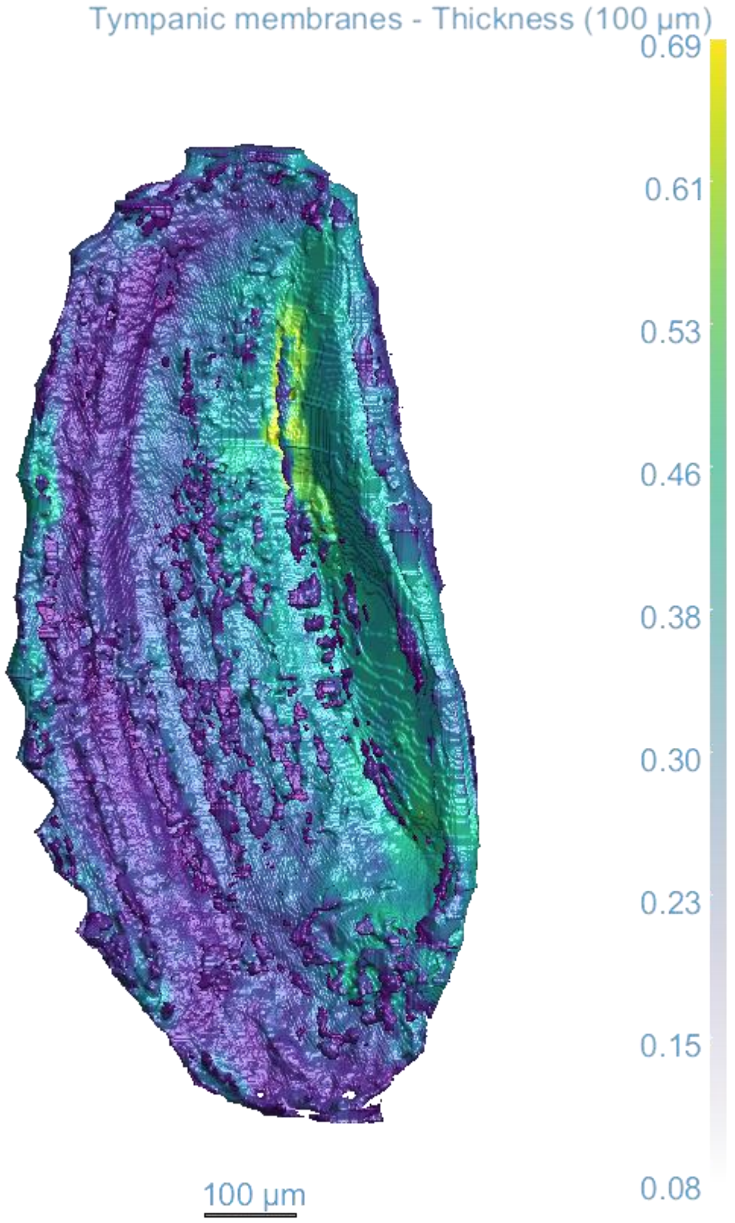

Arnobia pilipes

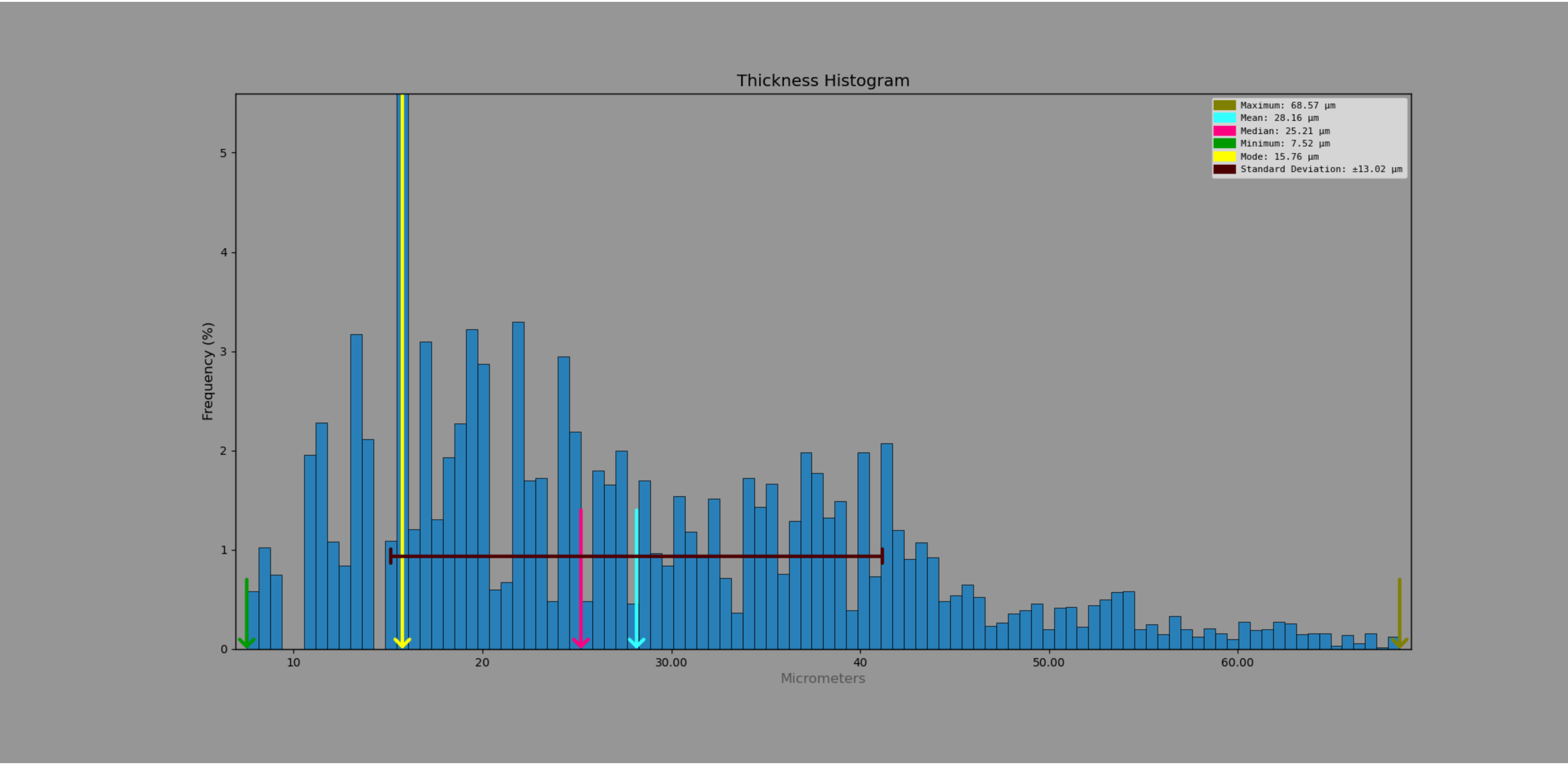

Arnobia pilipes – exposed tympana

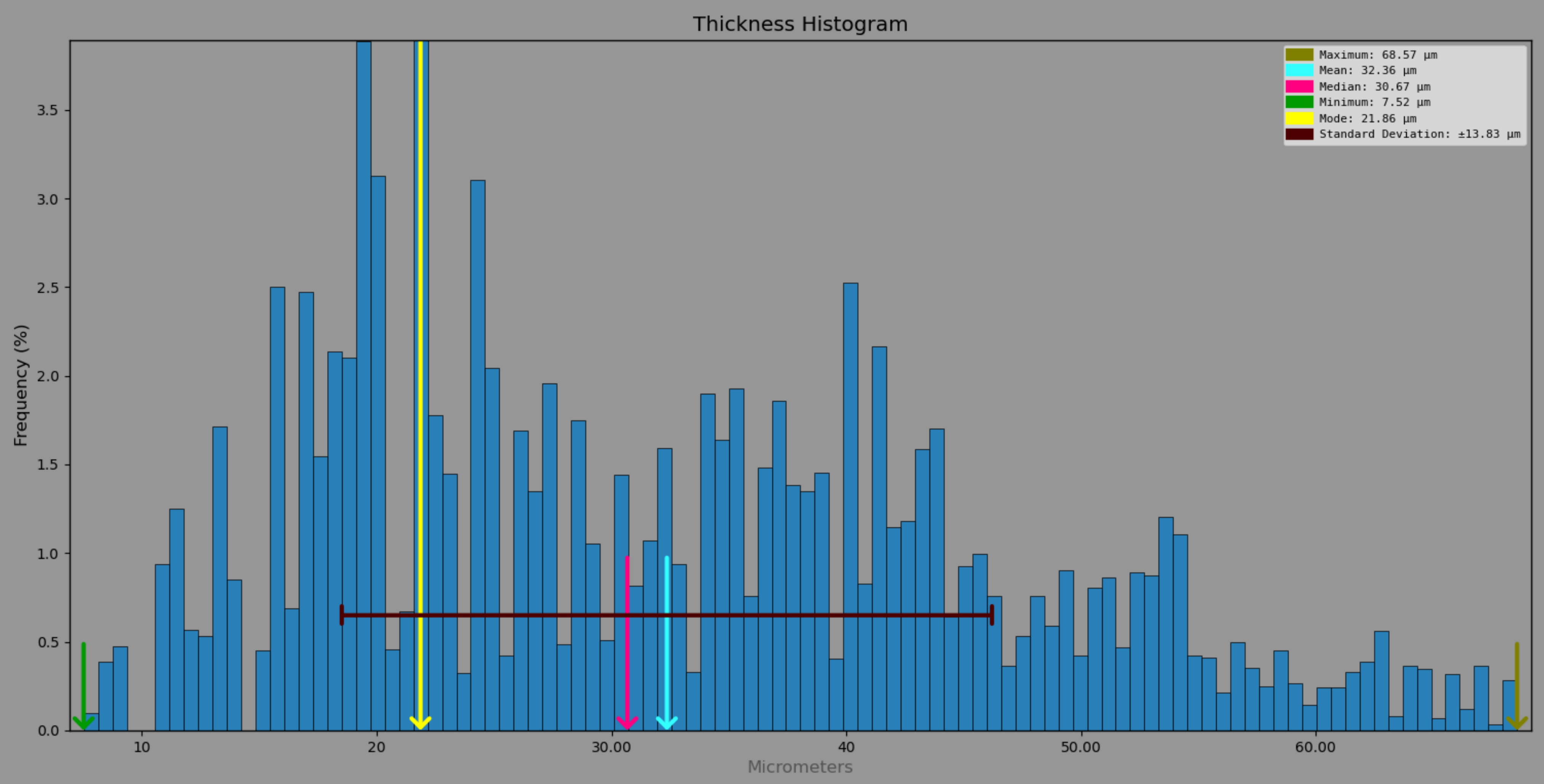

Arnobia pilipes – pinna covered tympana

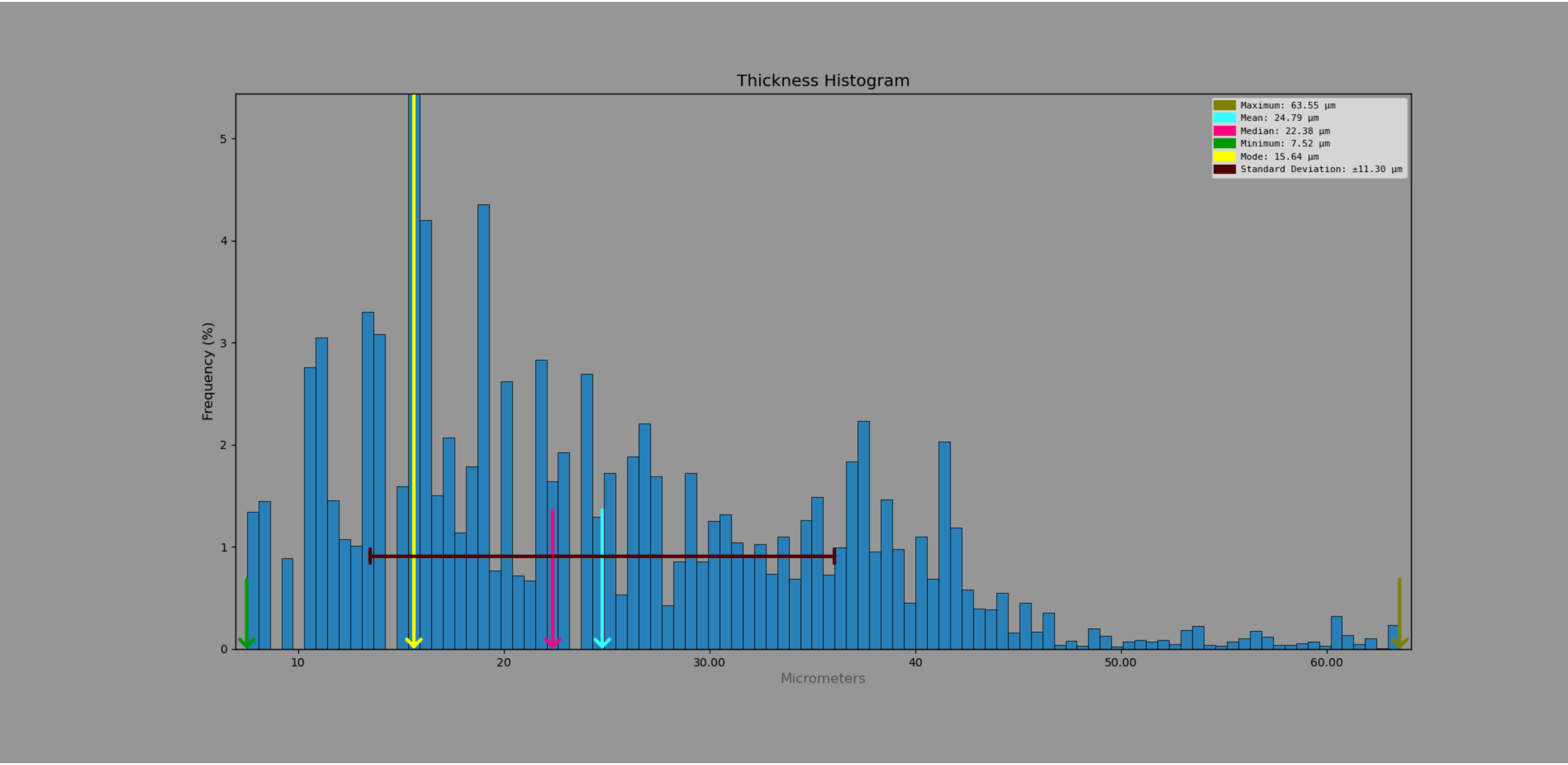

Balboana tibialis

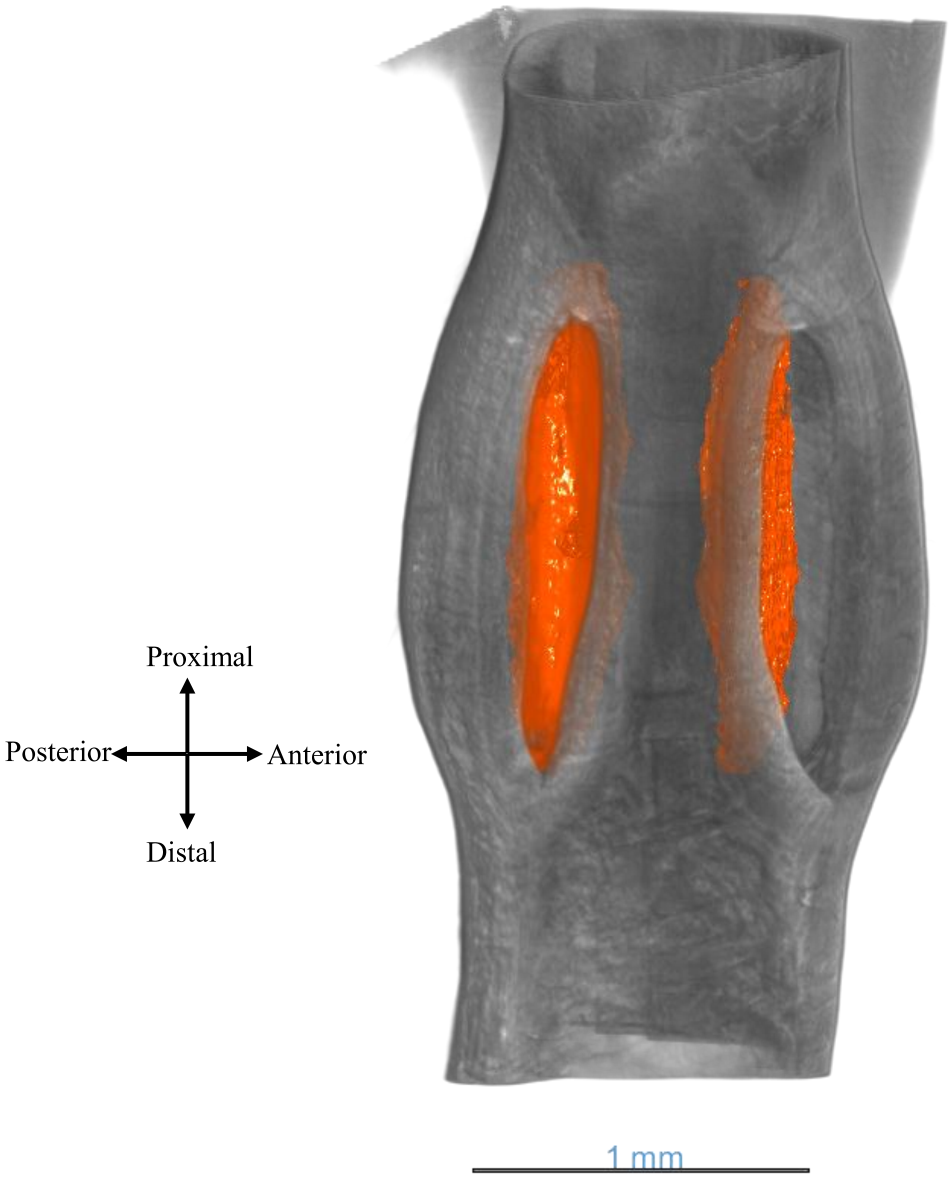

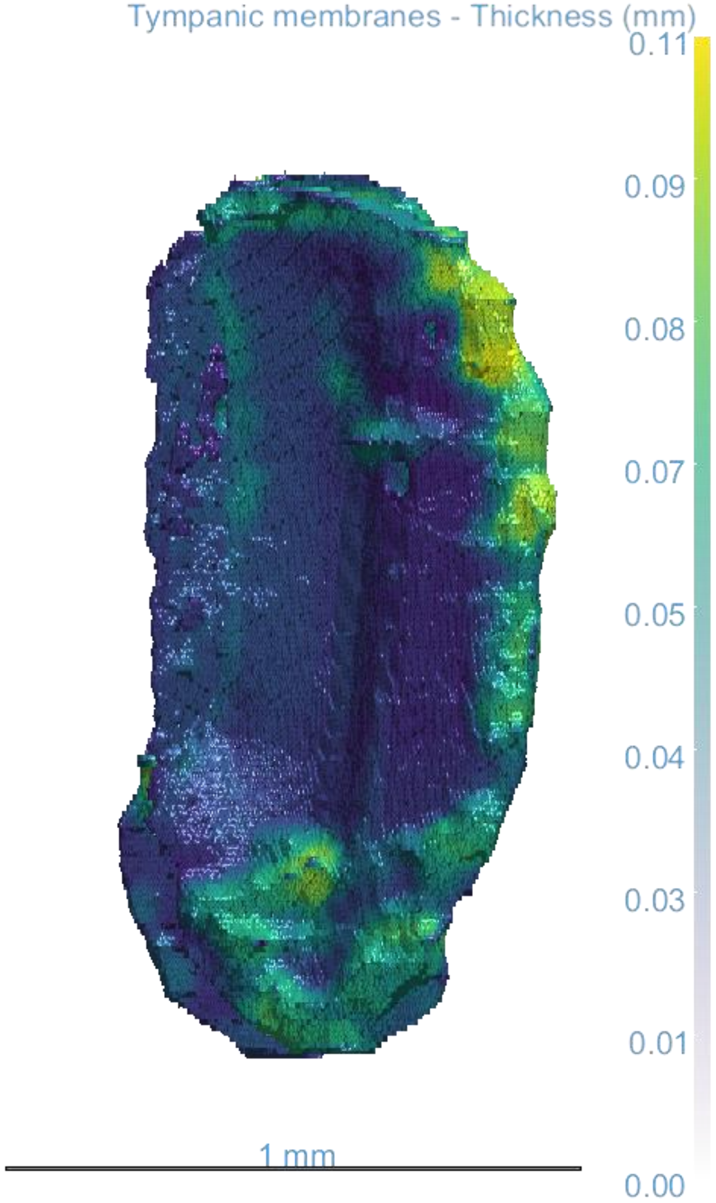

Balboana tibialis

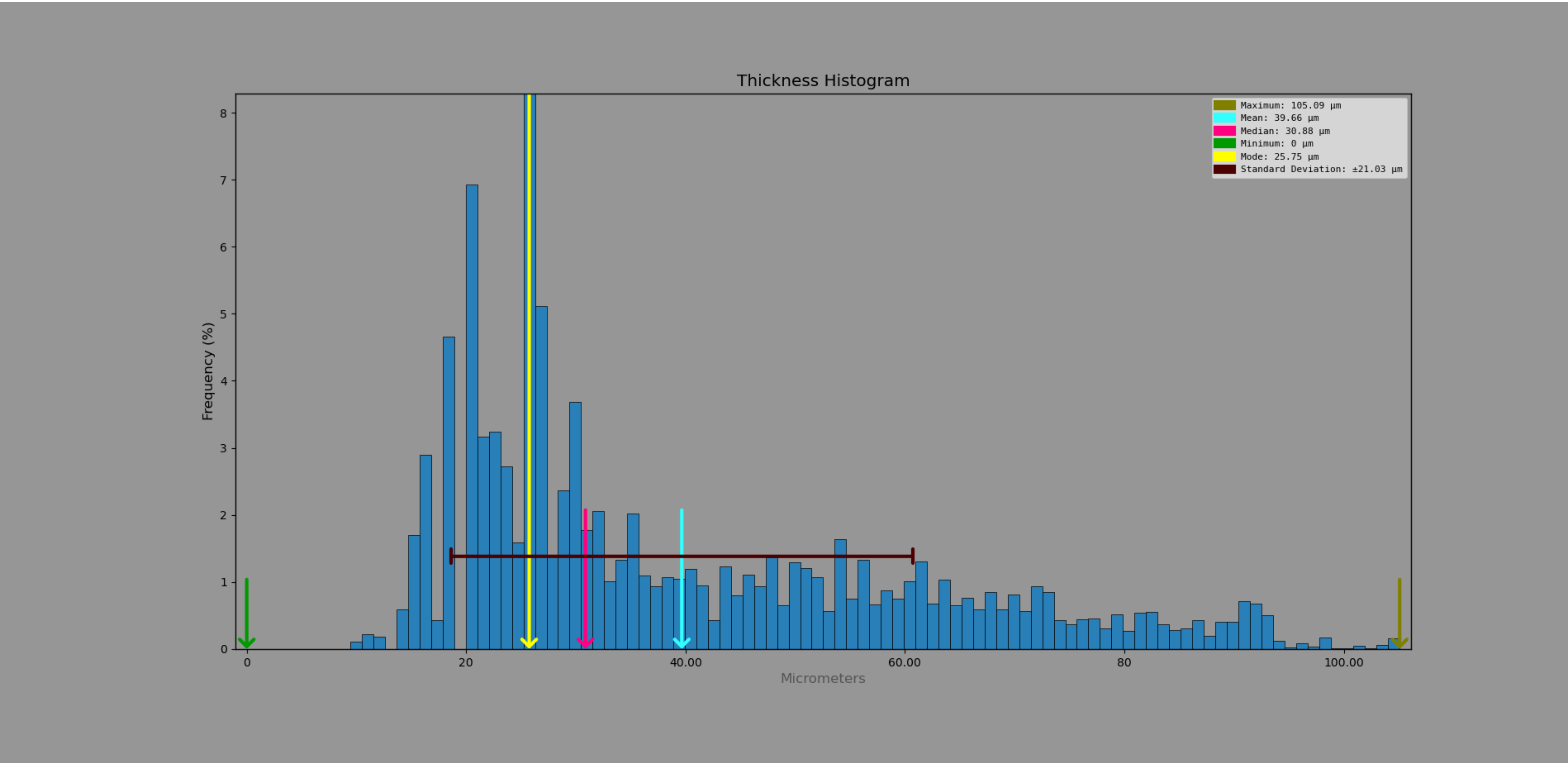

Chibchella nigrospecula

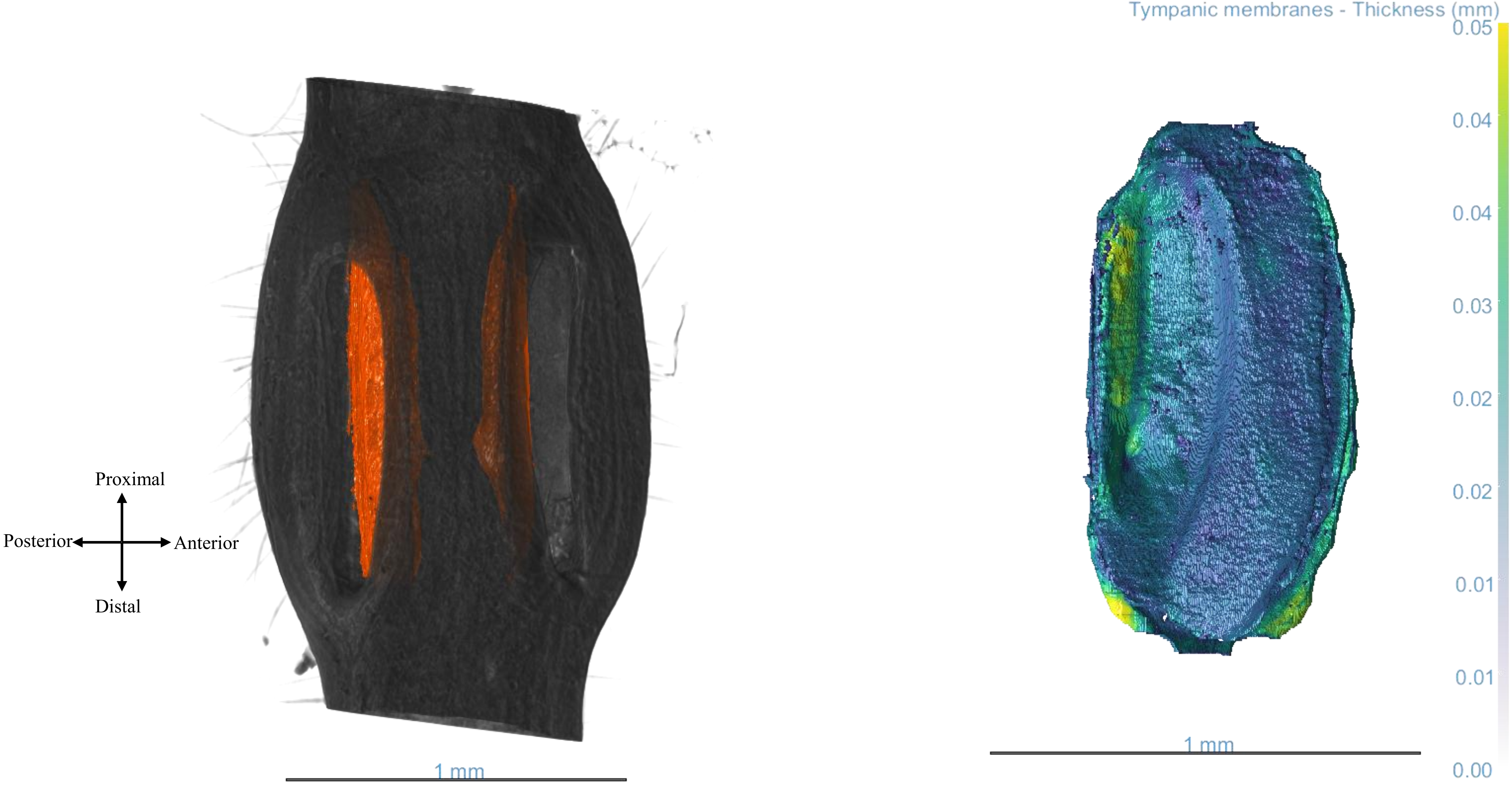

Chibchella nigrospecula

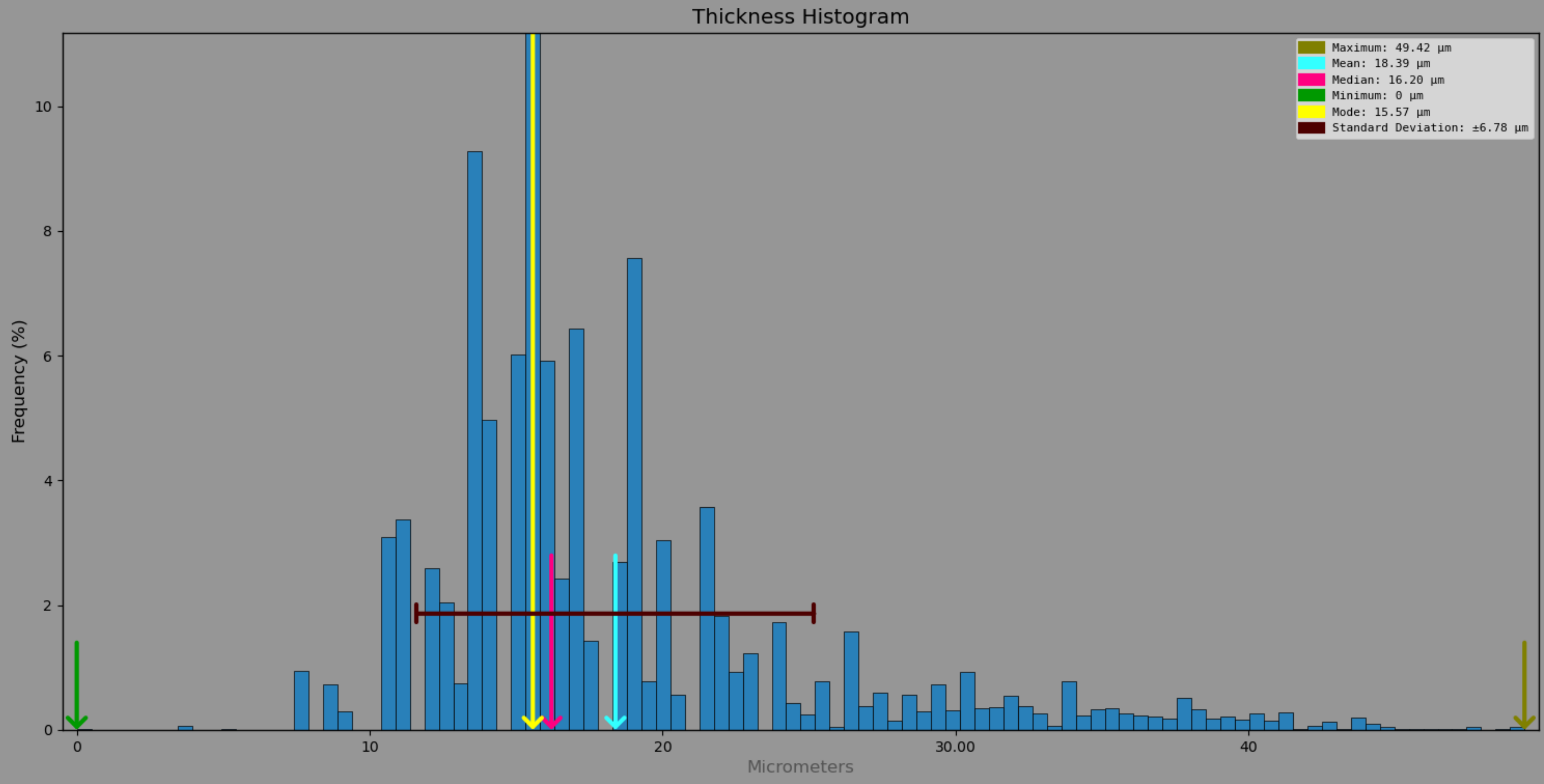

Copiphora gorgonensis

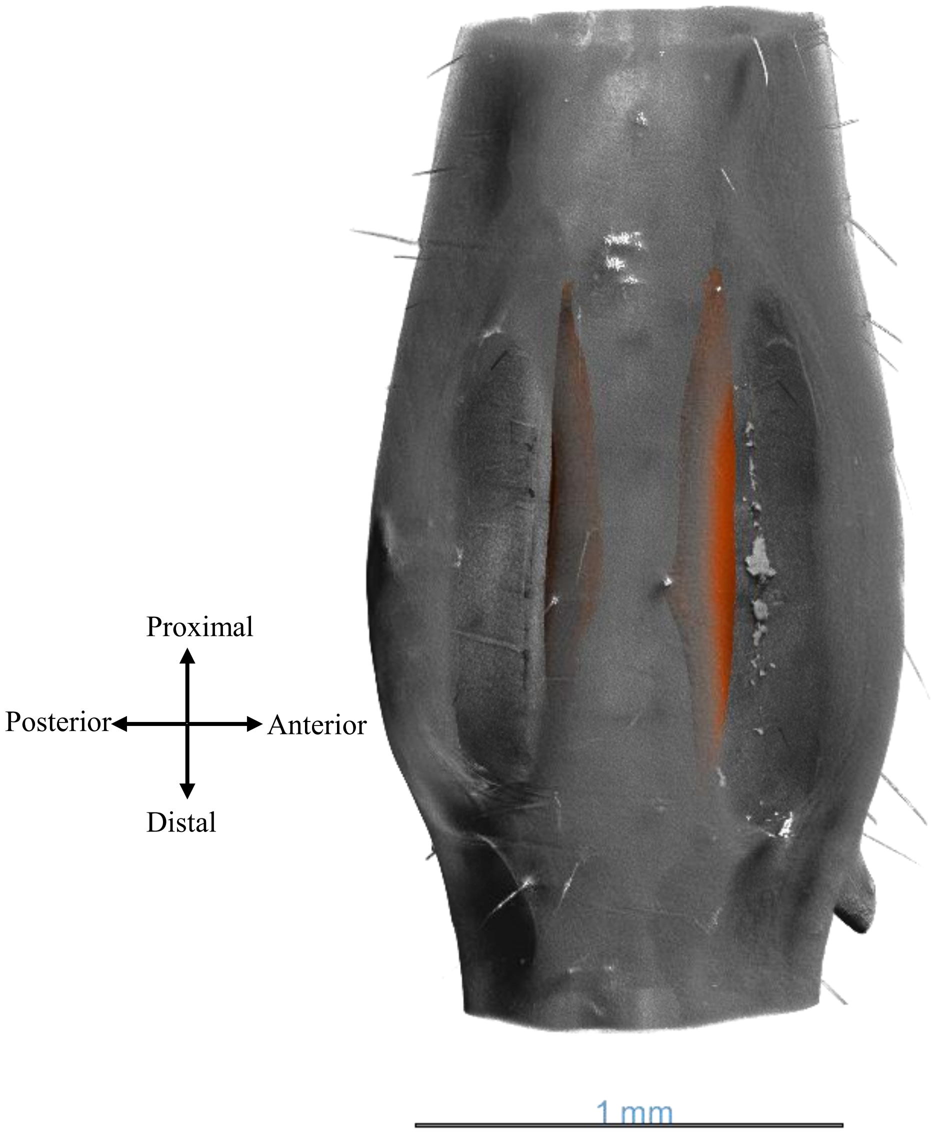

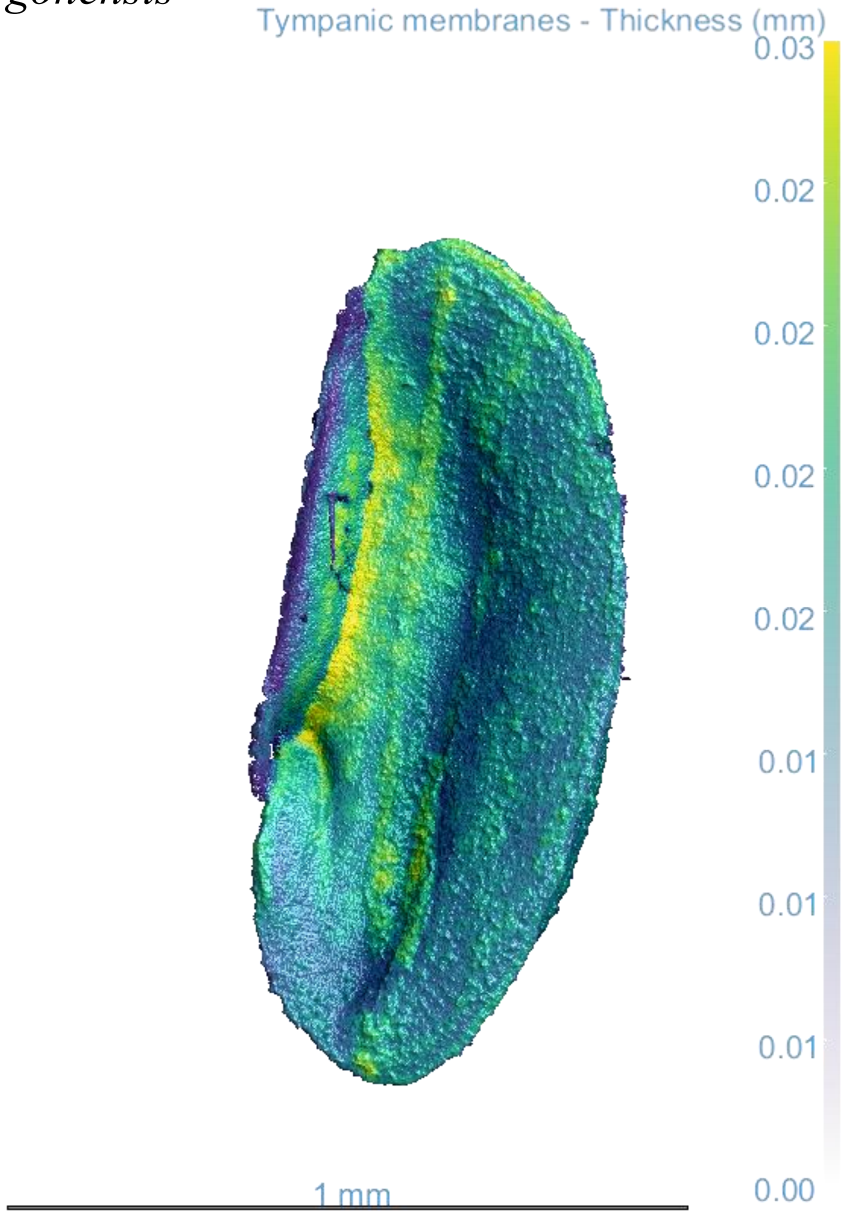

Copiphora gorgonensis

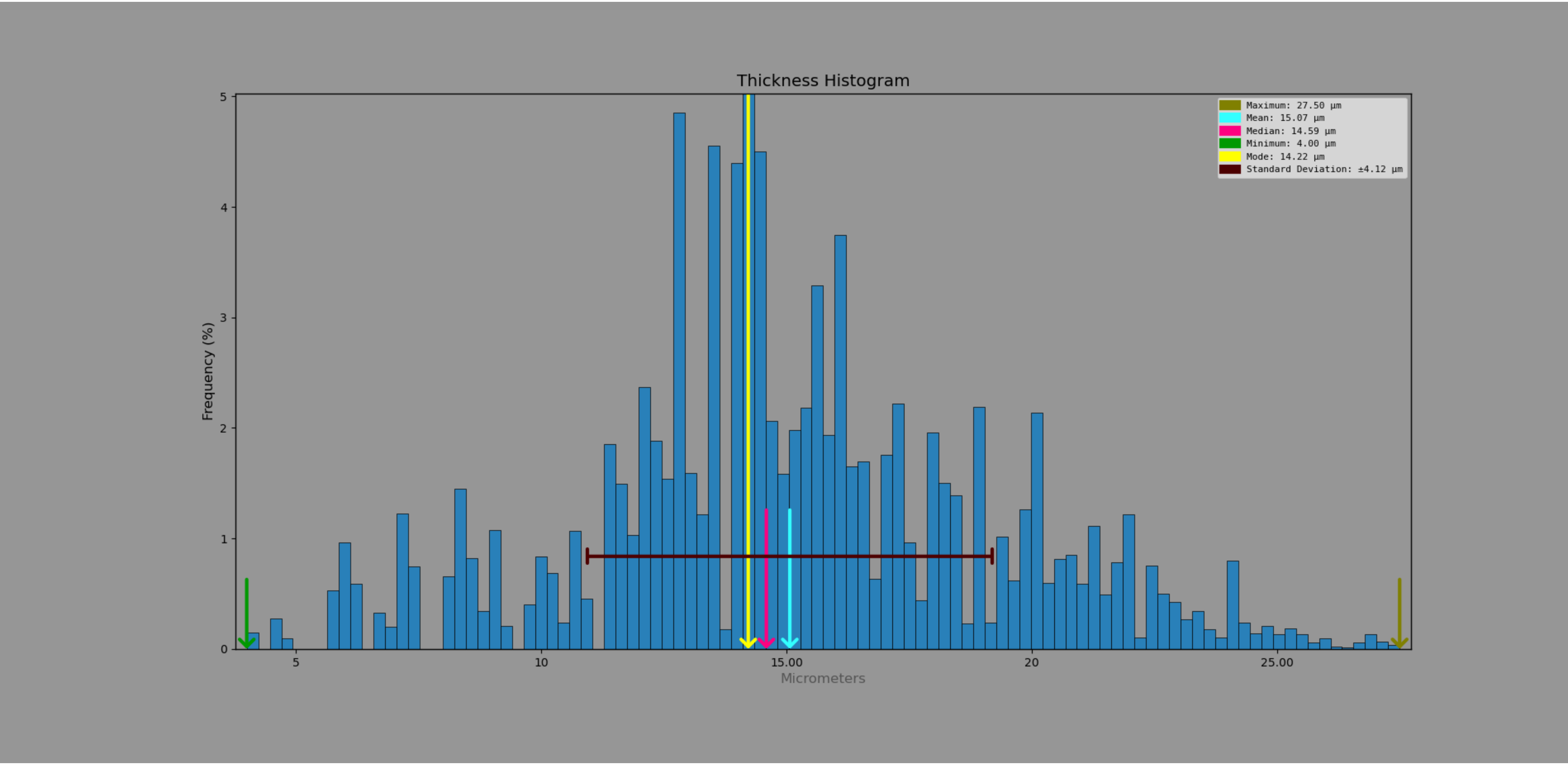

Elimaea signata

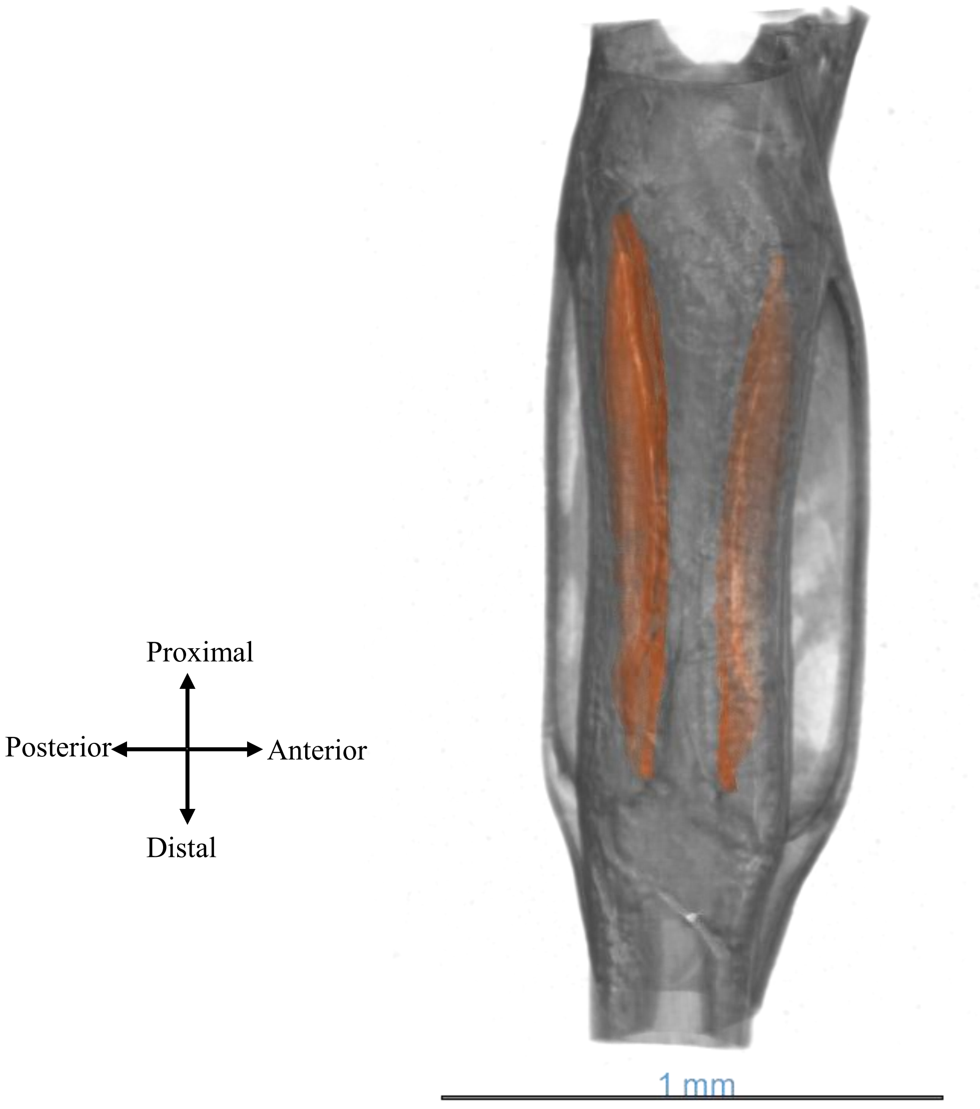

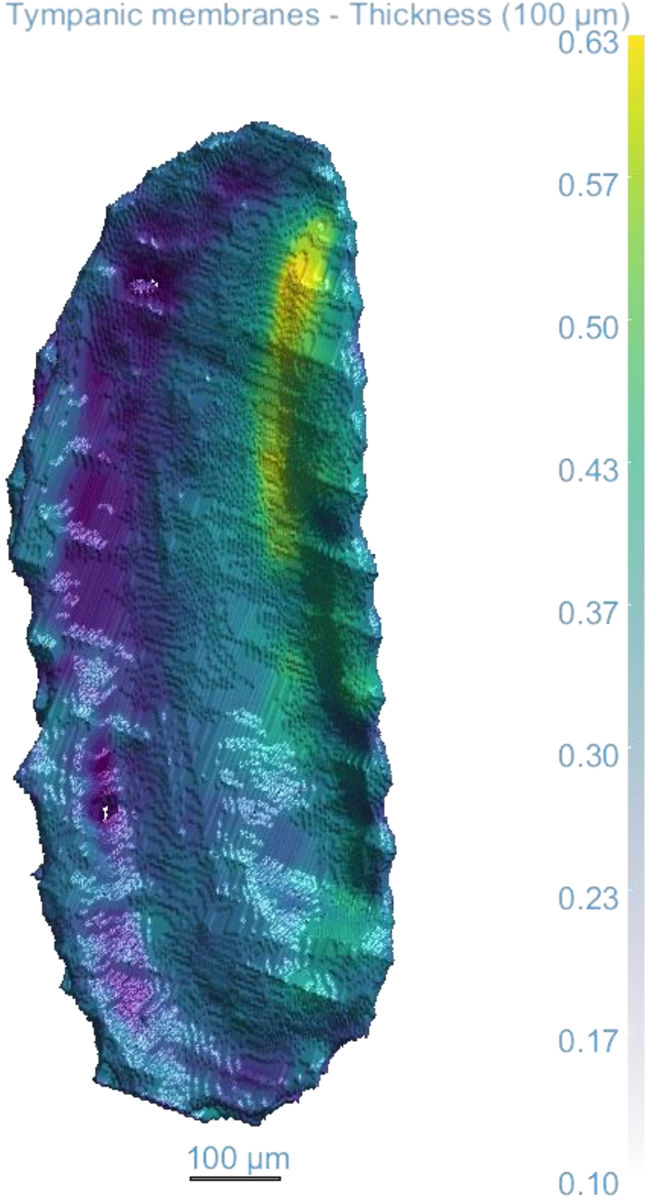

Elimaea signata

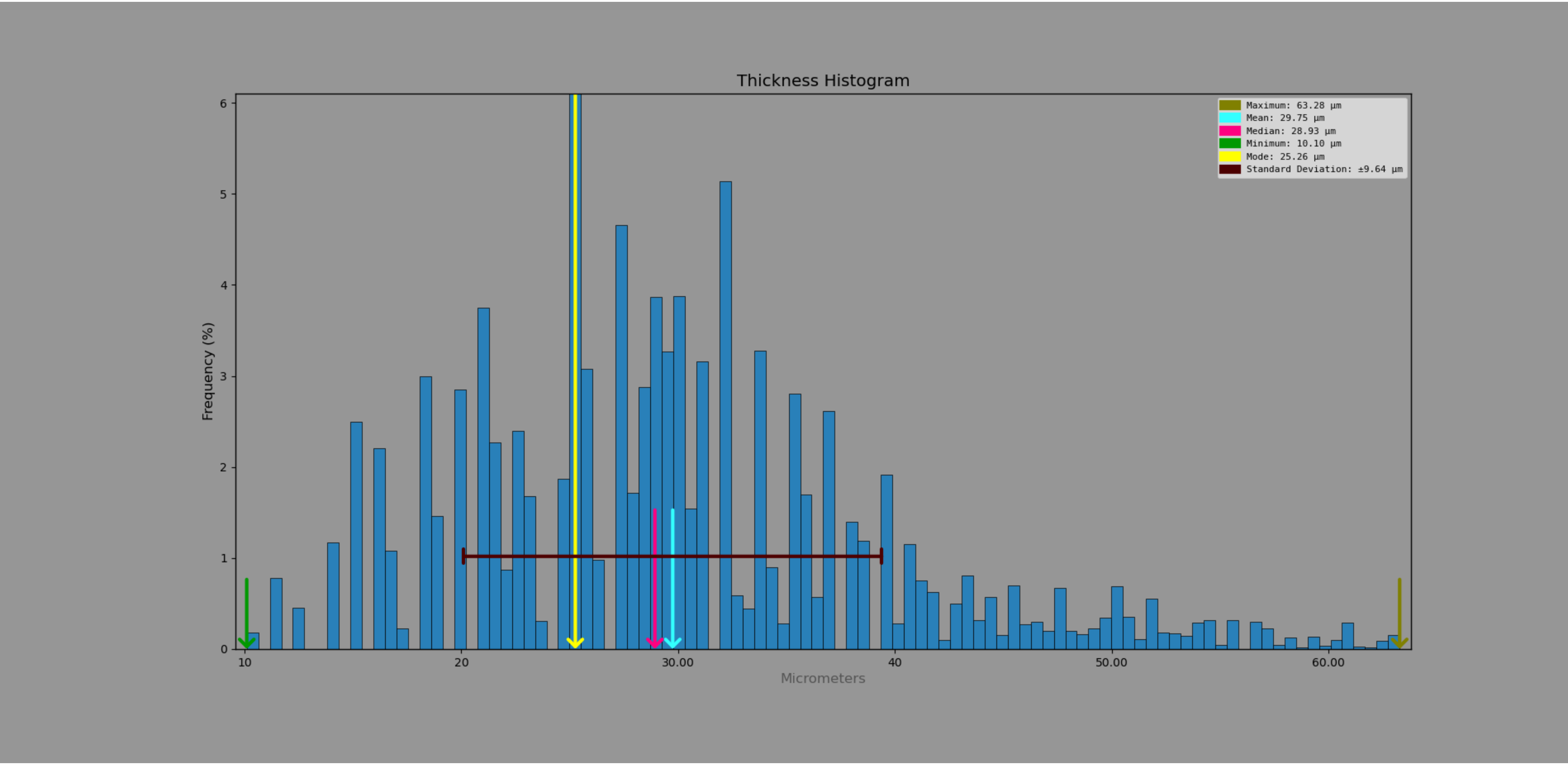

Haenschiella sp.

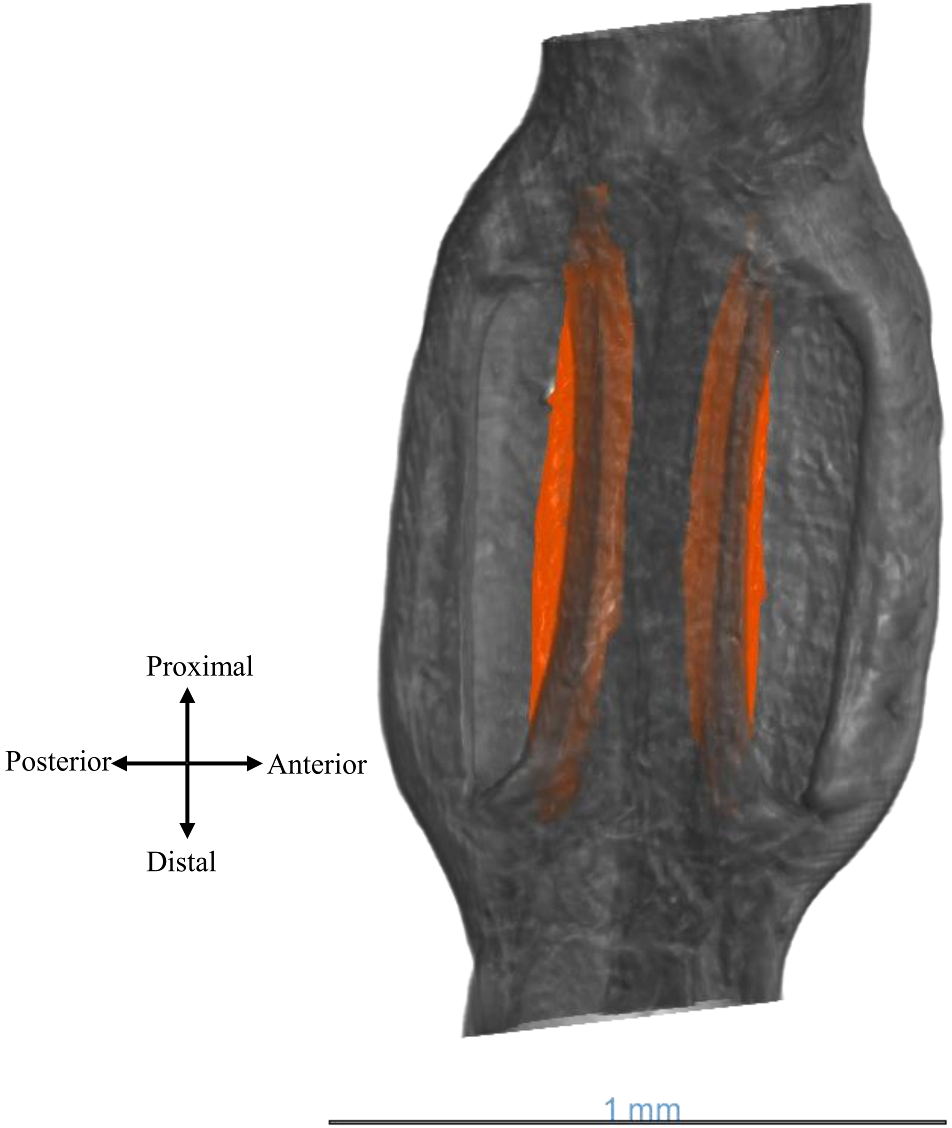

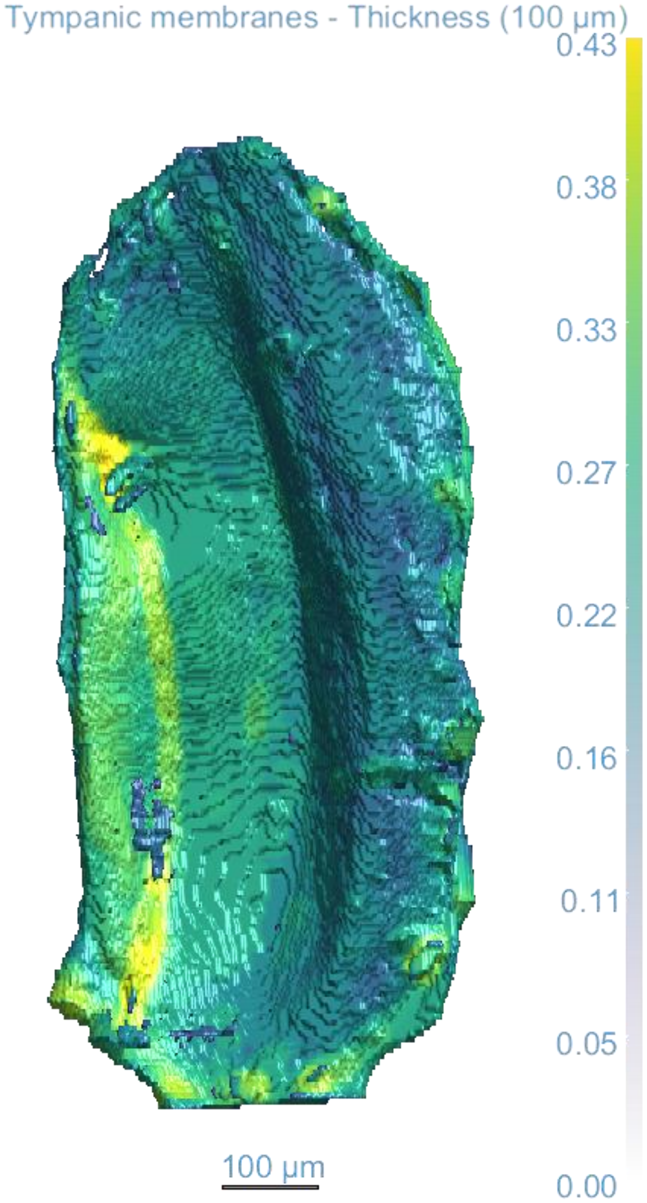

Haenschiella sp.

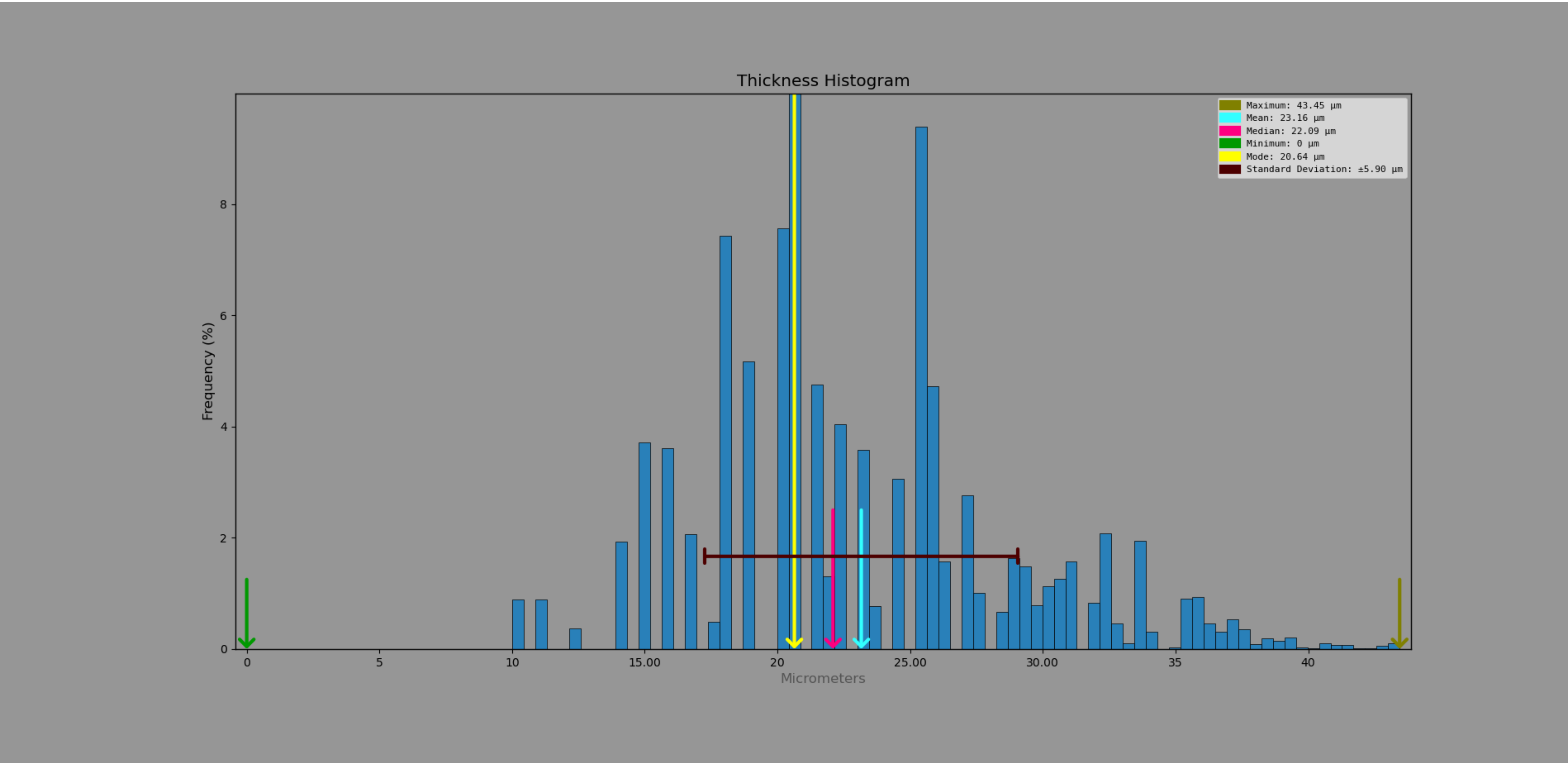

Leptoderes ornatipennis

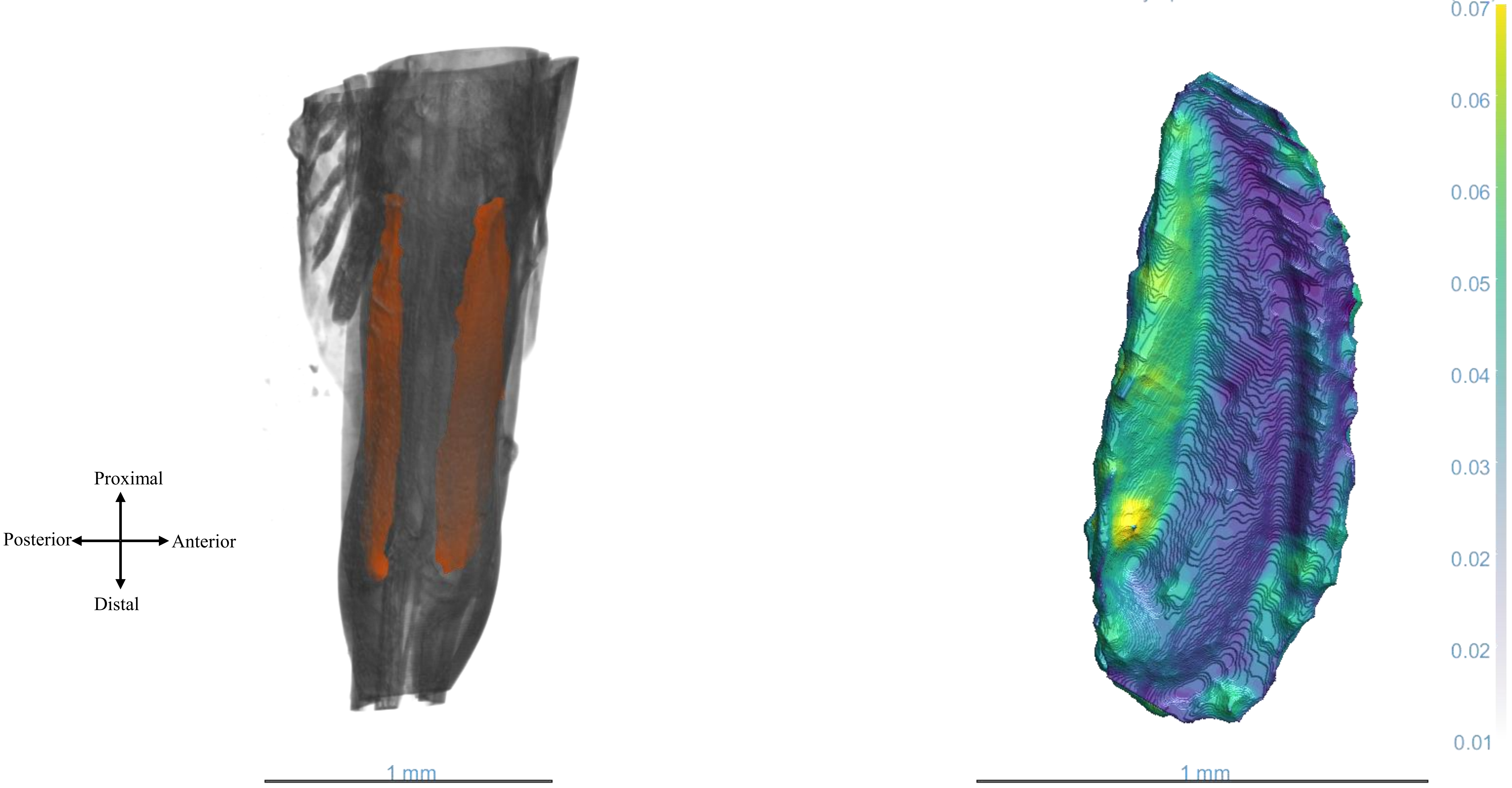

Leptoderes ornatipennis

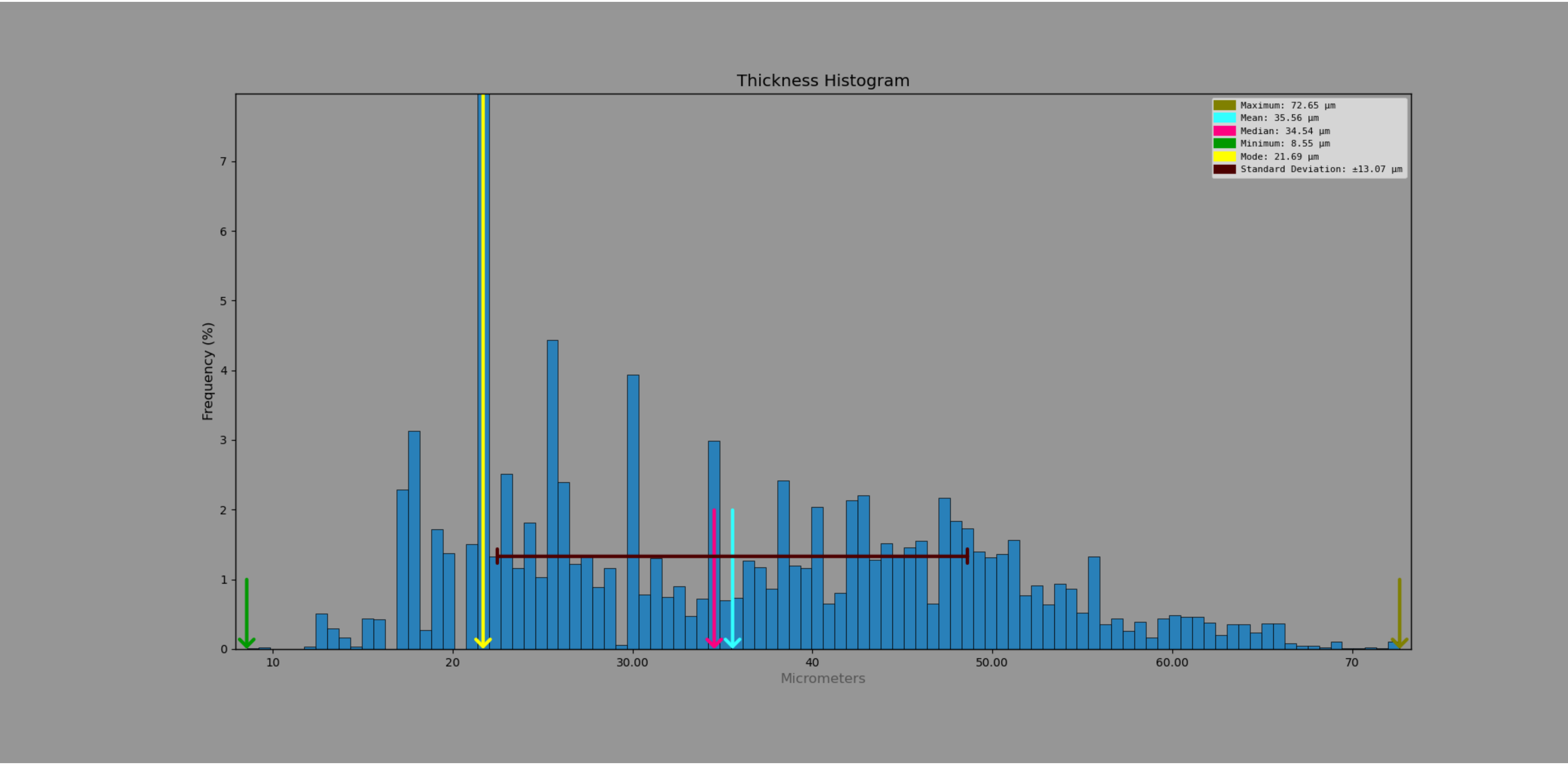

Mecopoda elongata

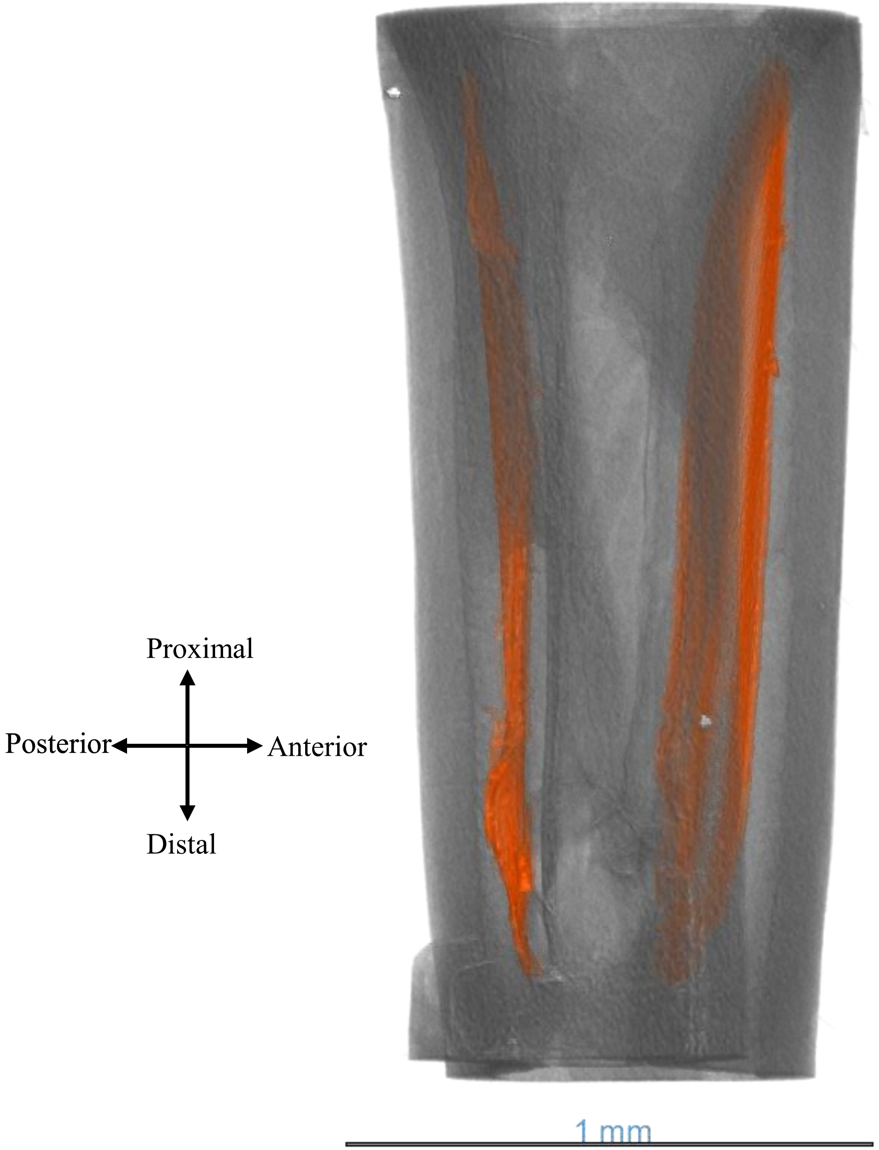

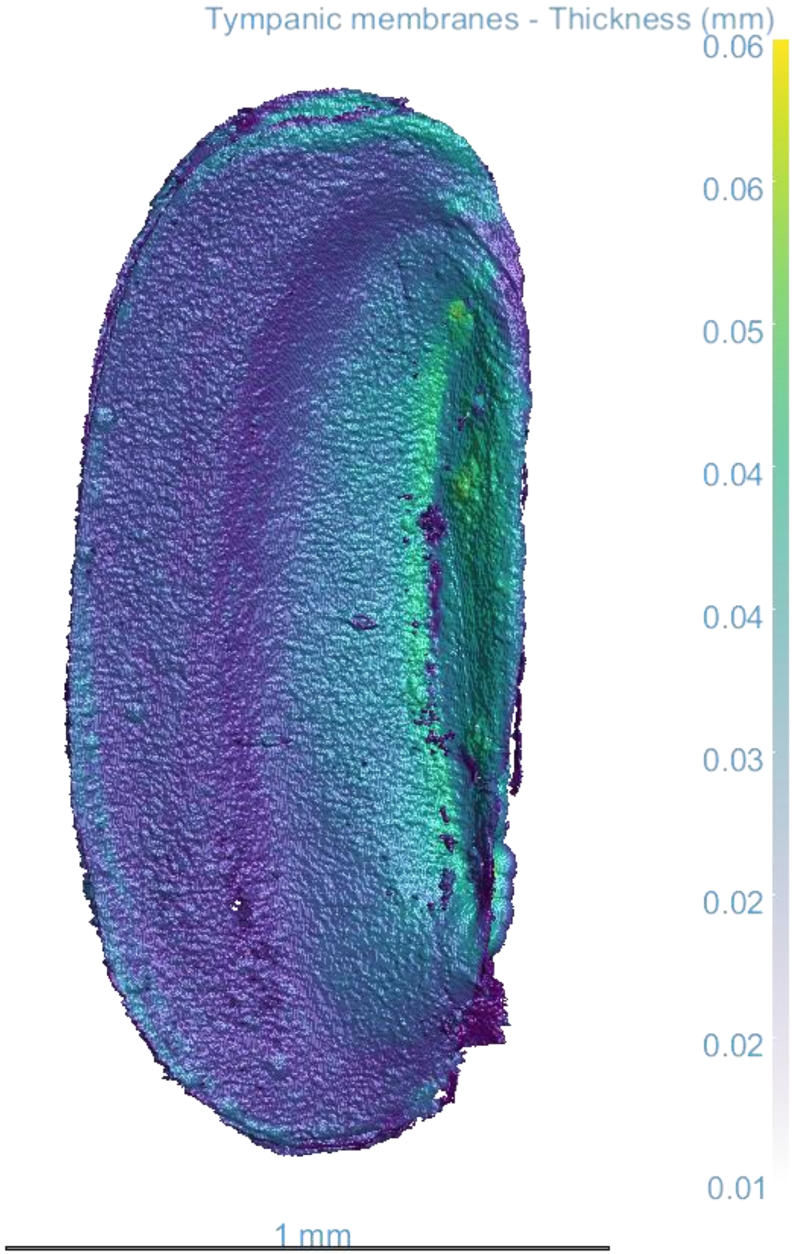

Mecopoda elongata

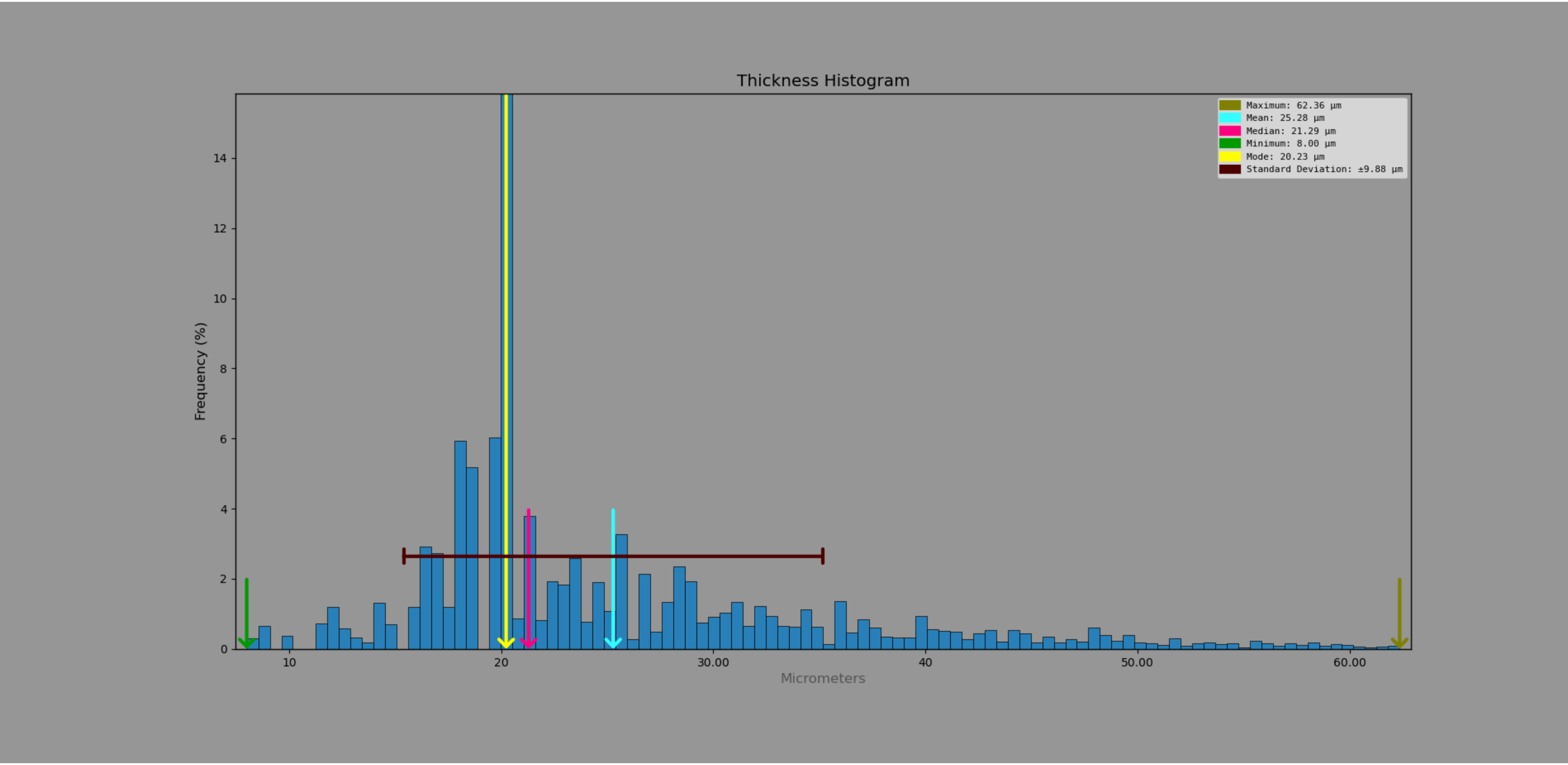

Moncheca elegans

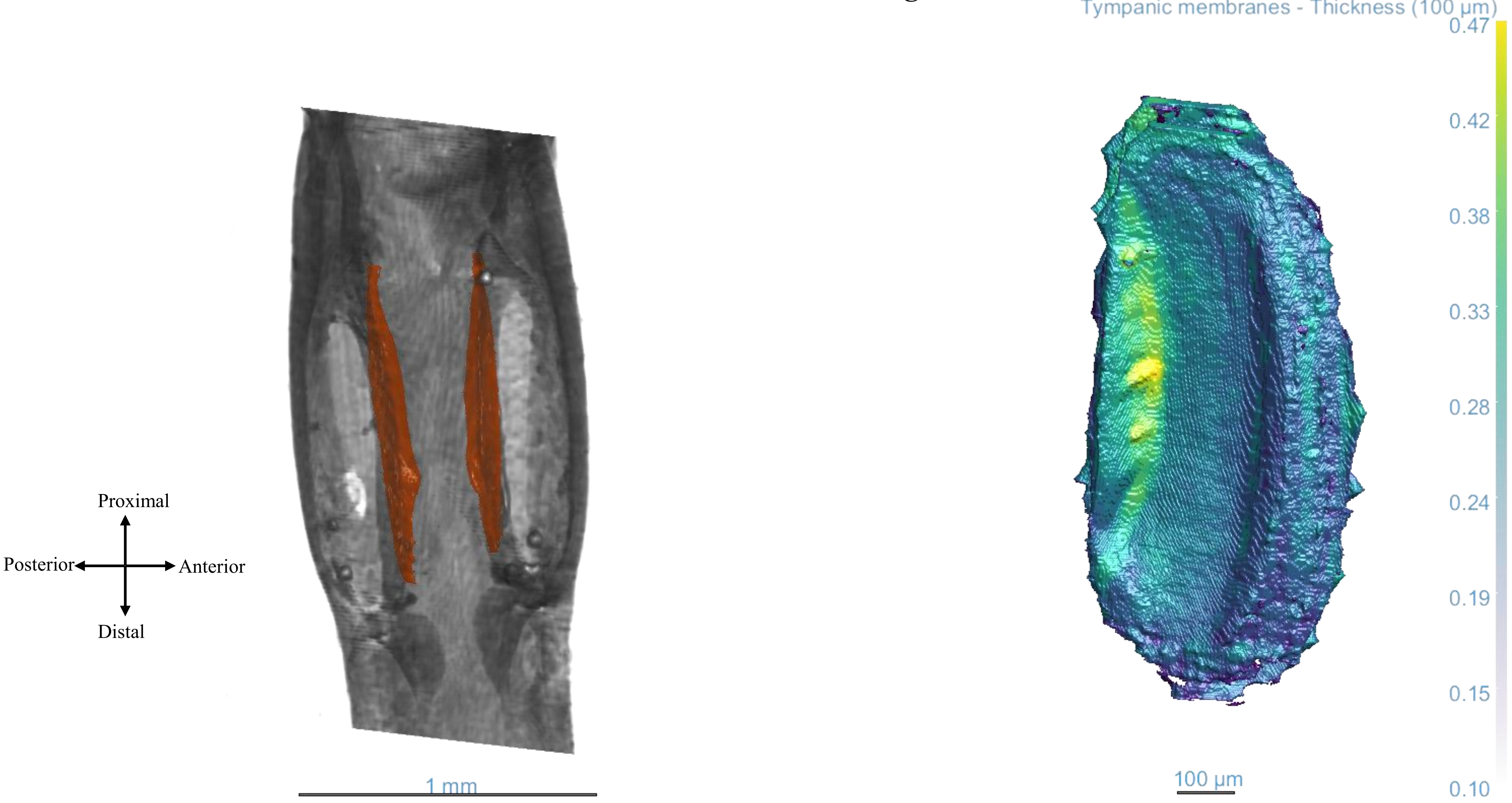

Moncheca elegans

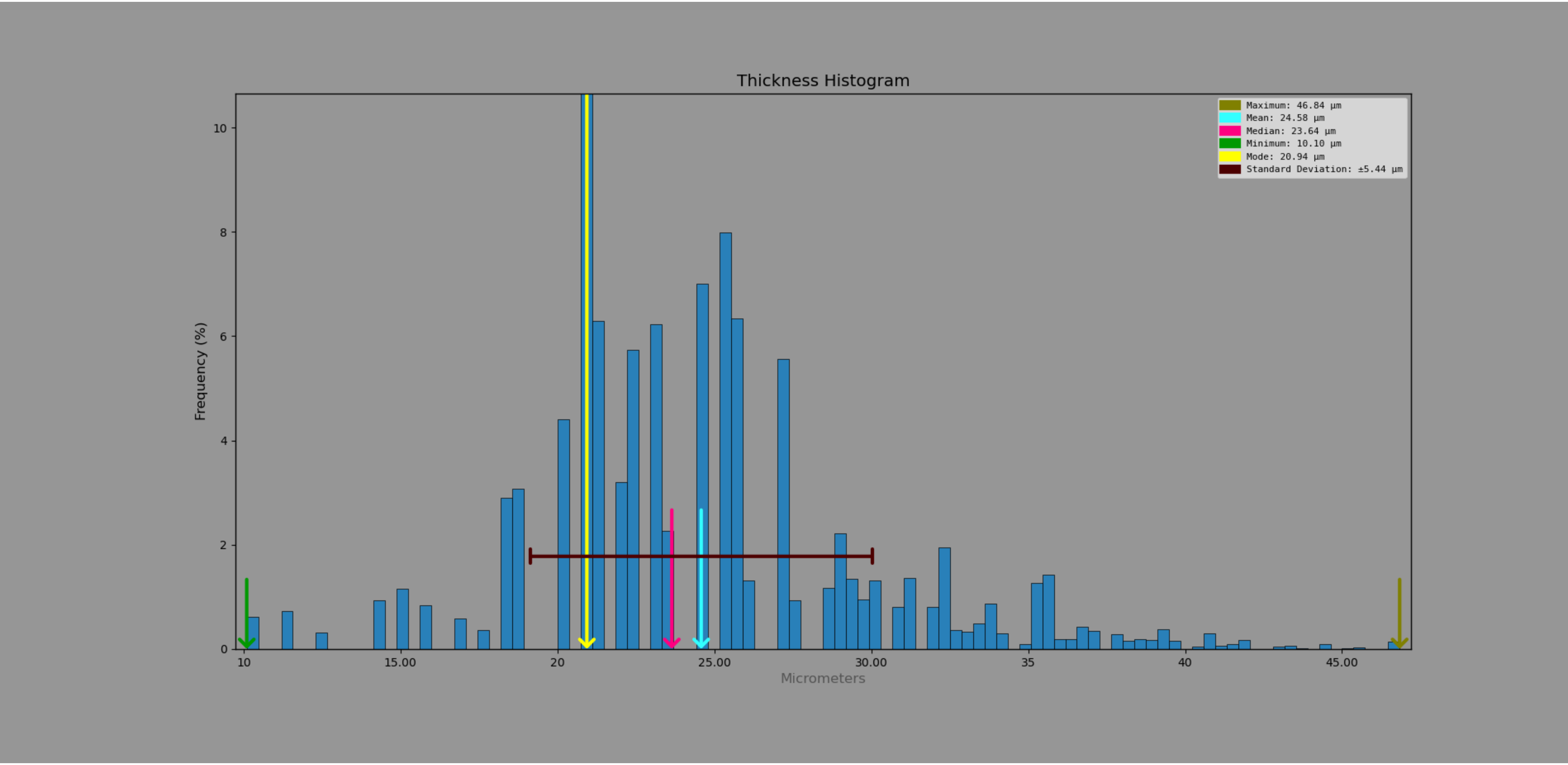

Phaulula galeata

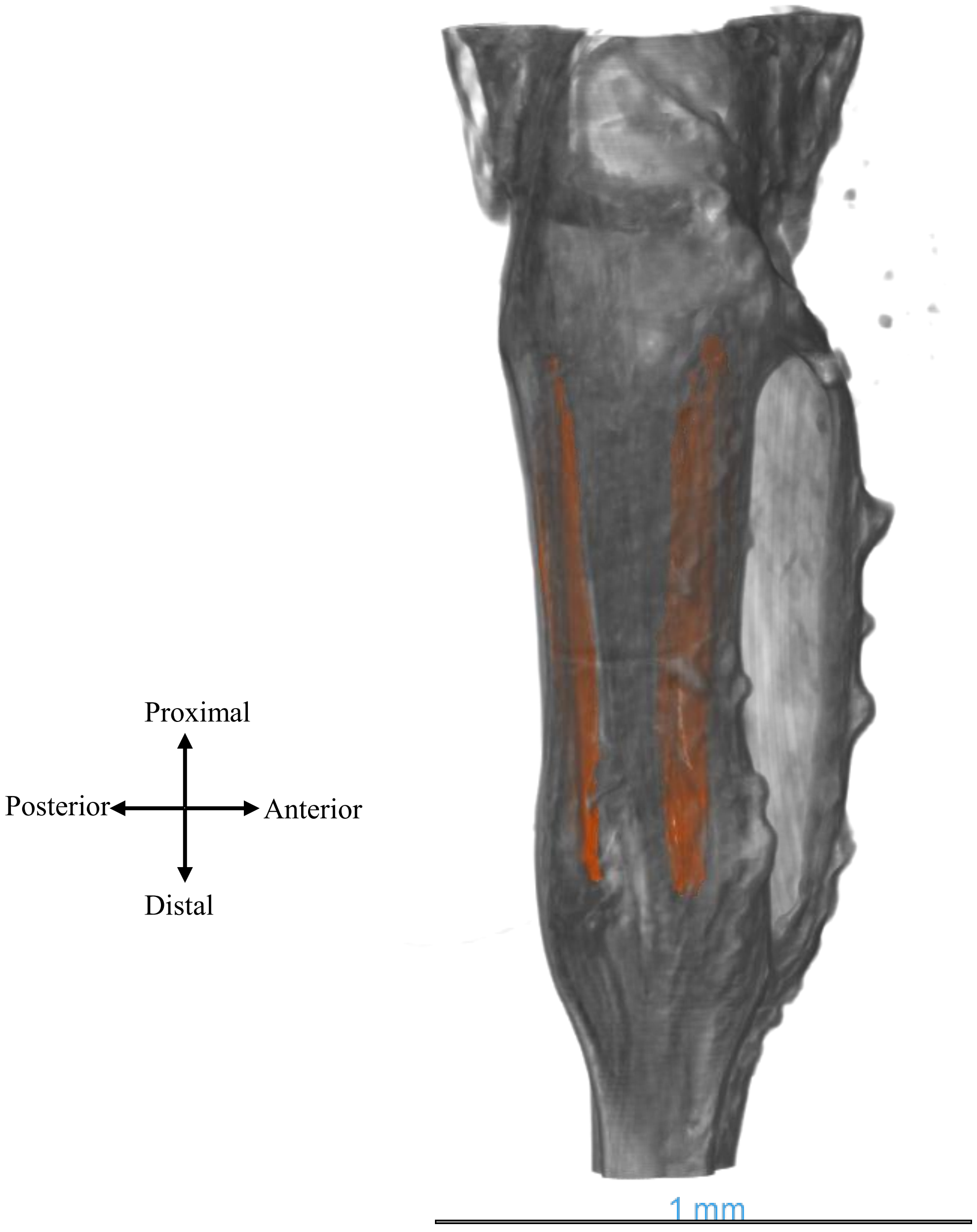

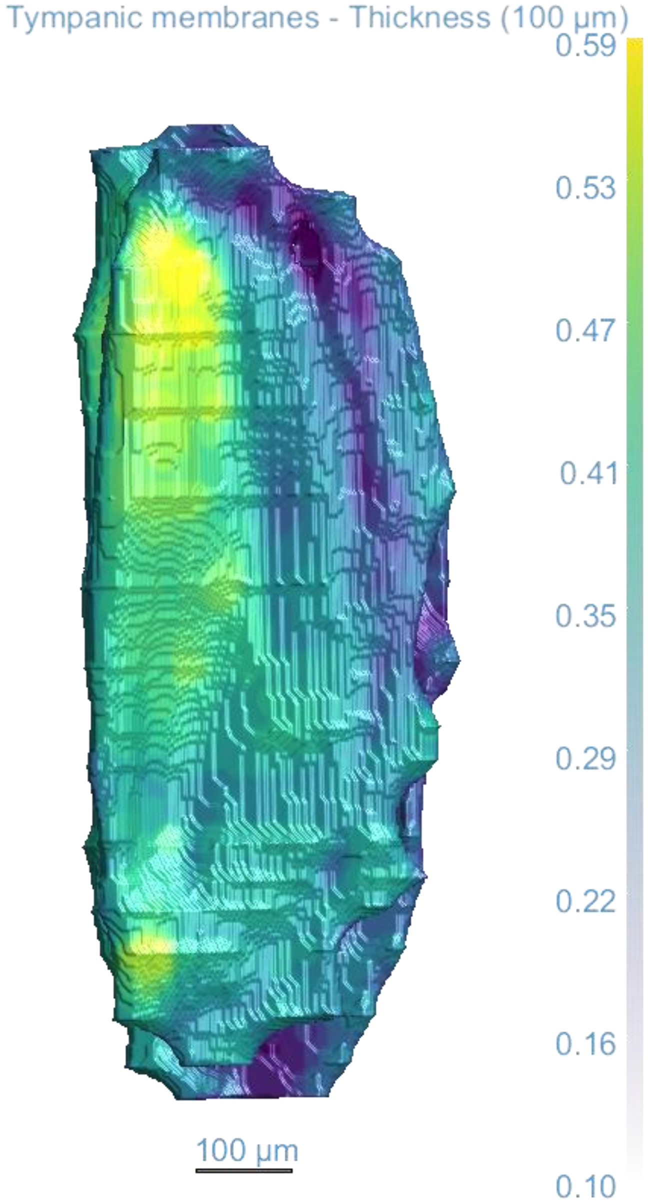

Phaulula galeata

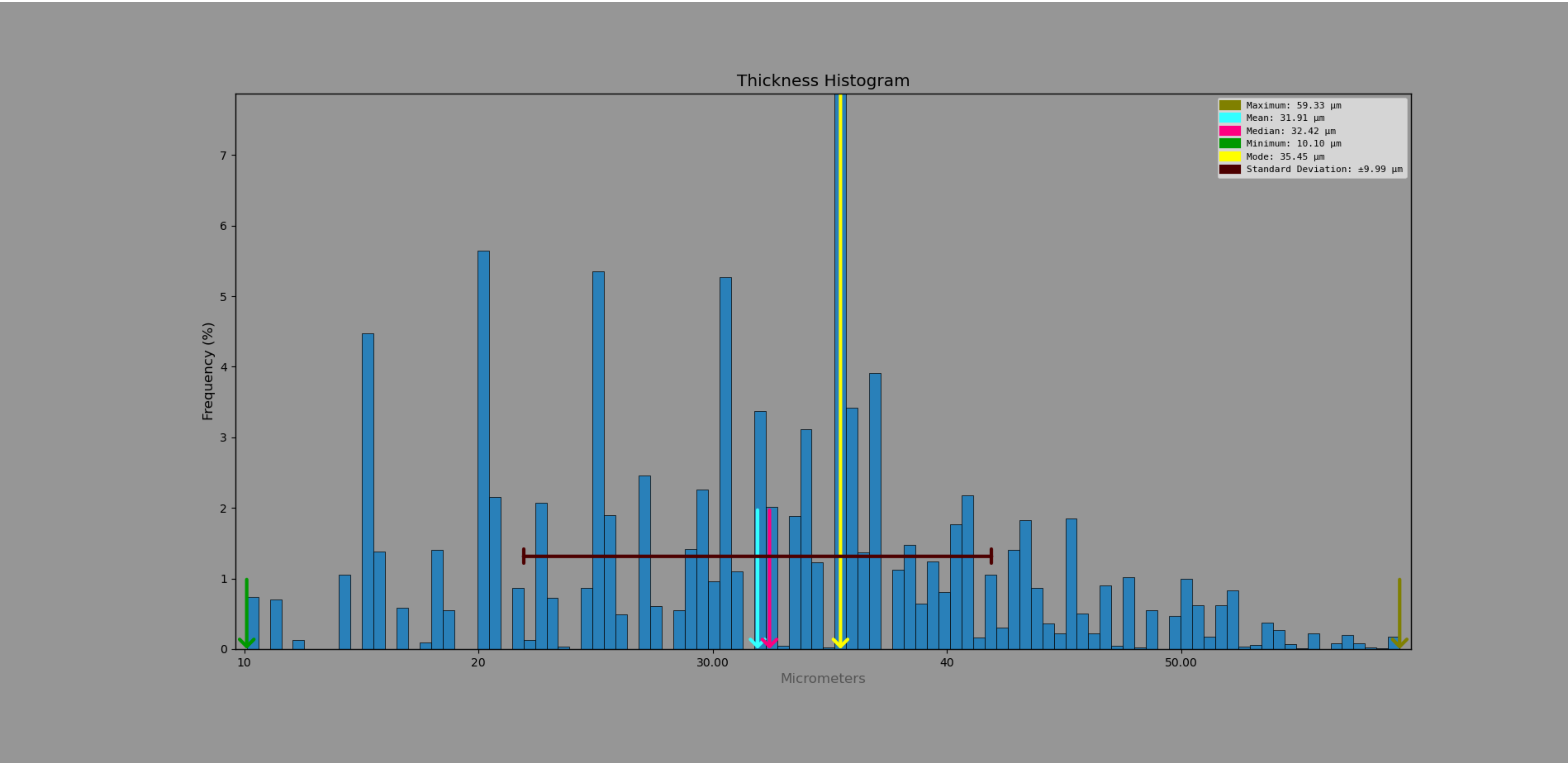

Phaulula galeata – exposed tympana

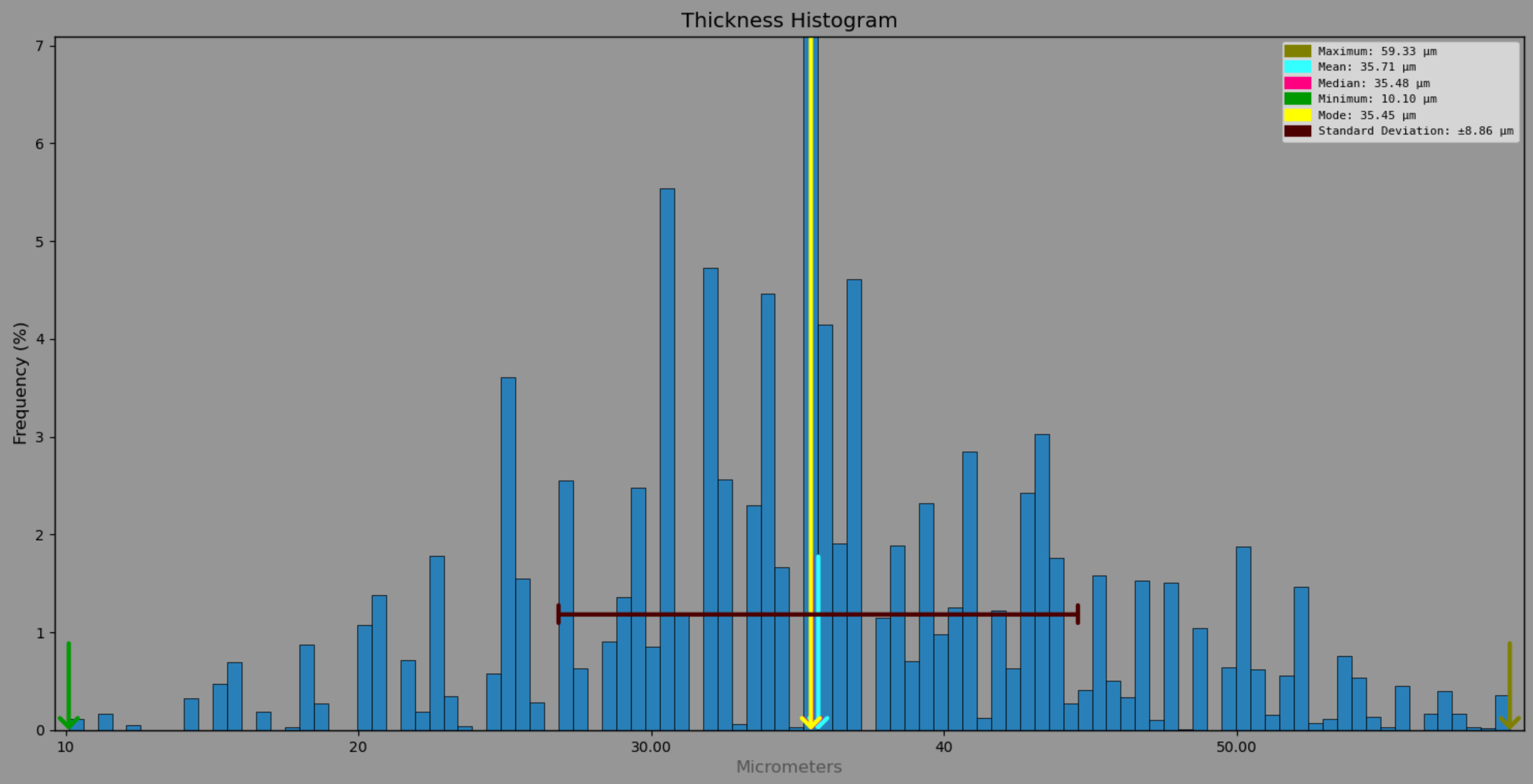

Phaulula galeata – pinna covered tympana

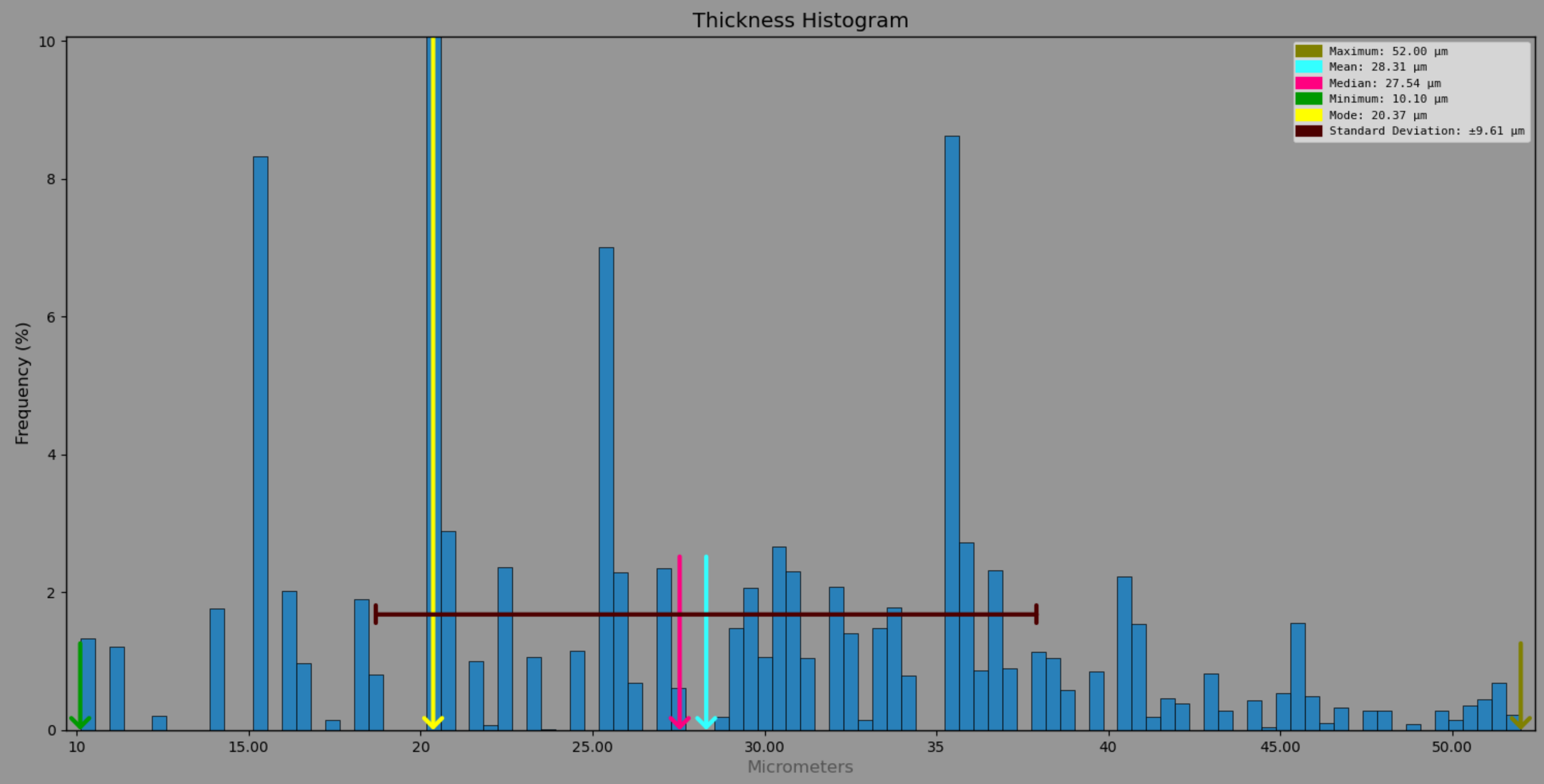

Phlugis poecilla

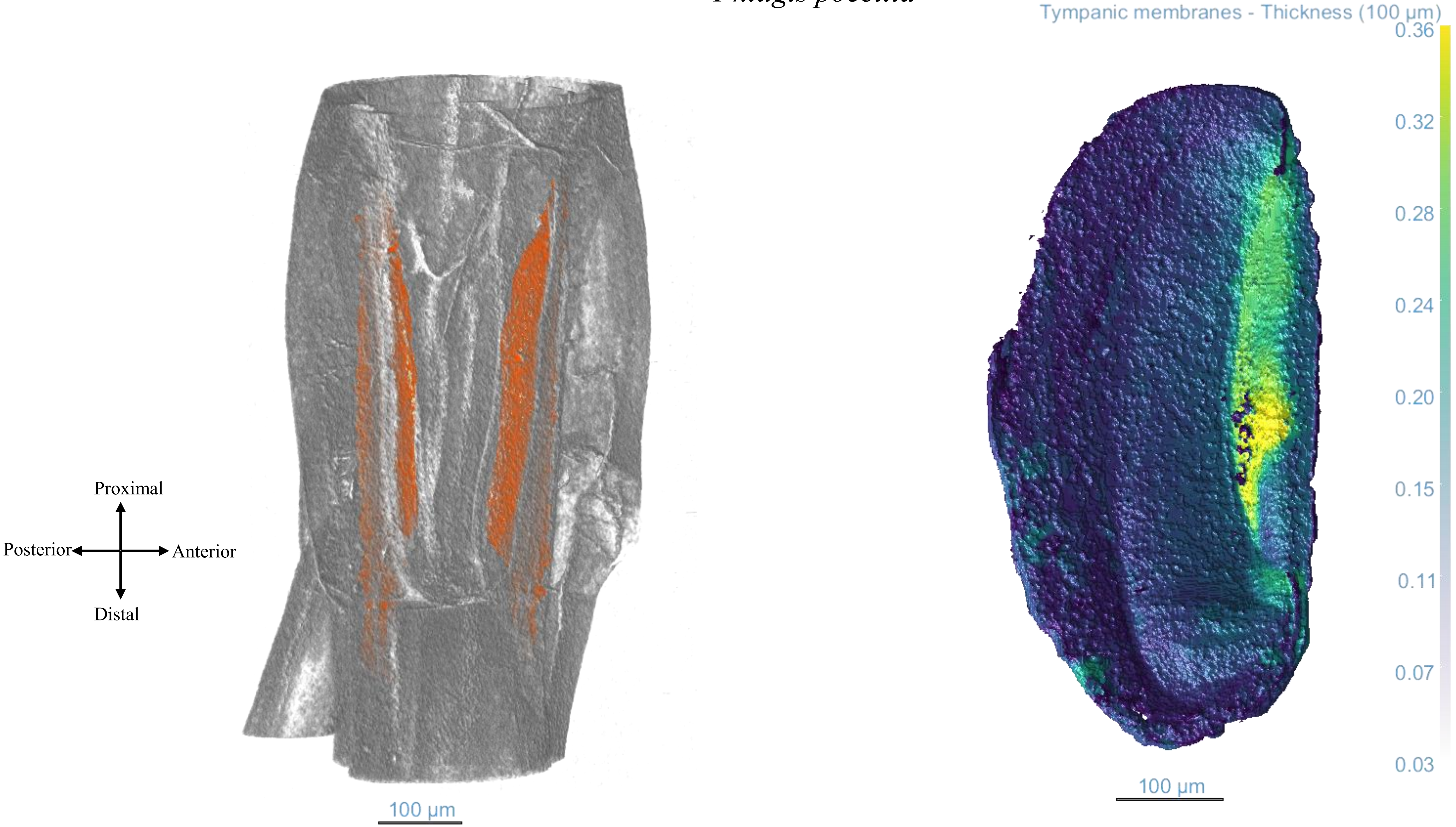

Phlugis poecilla

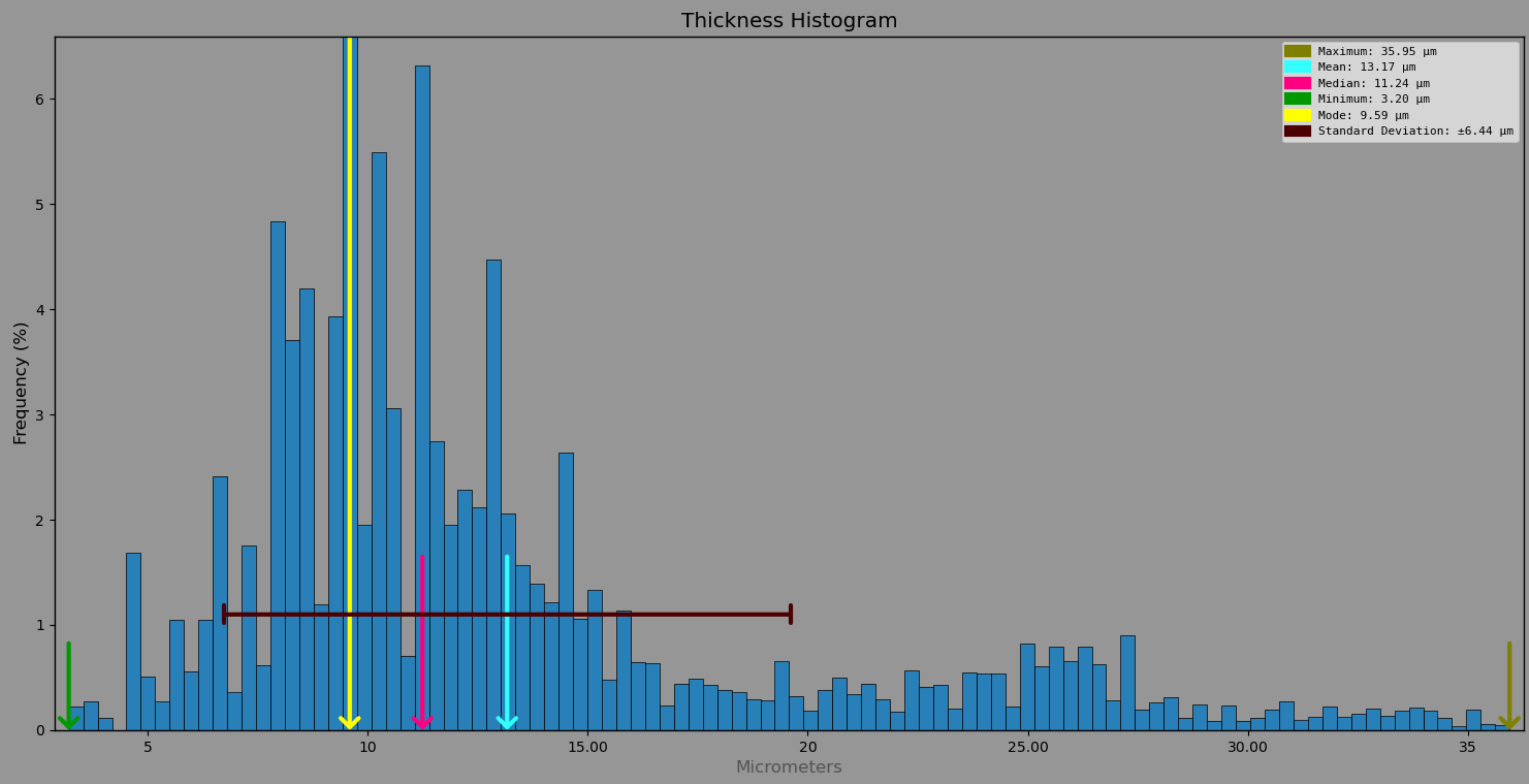

Phygela marginata

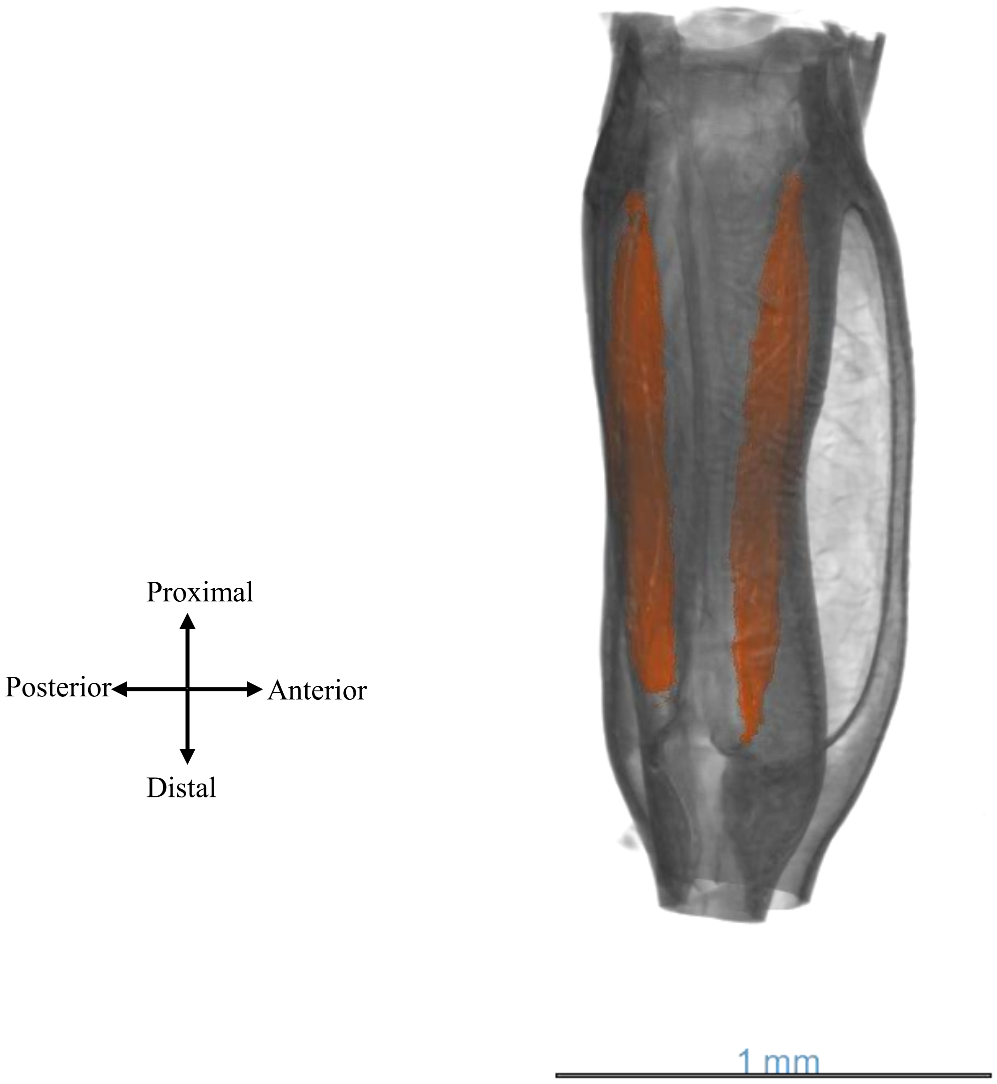

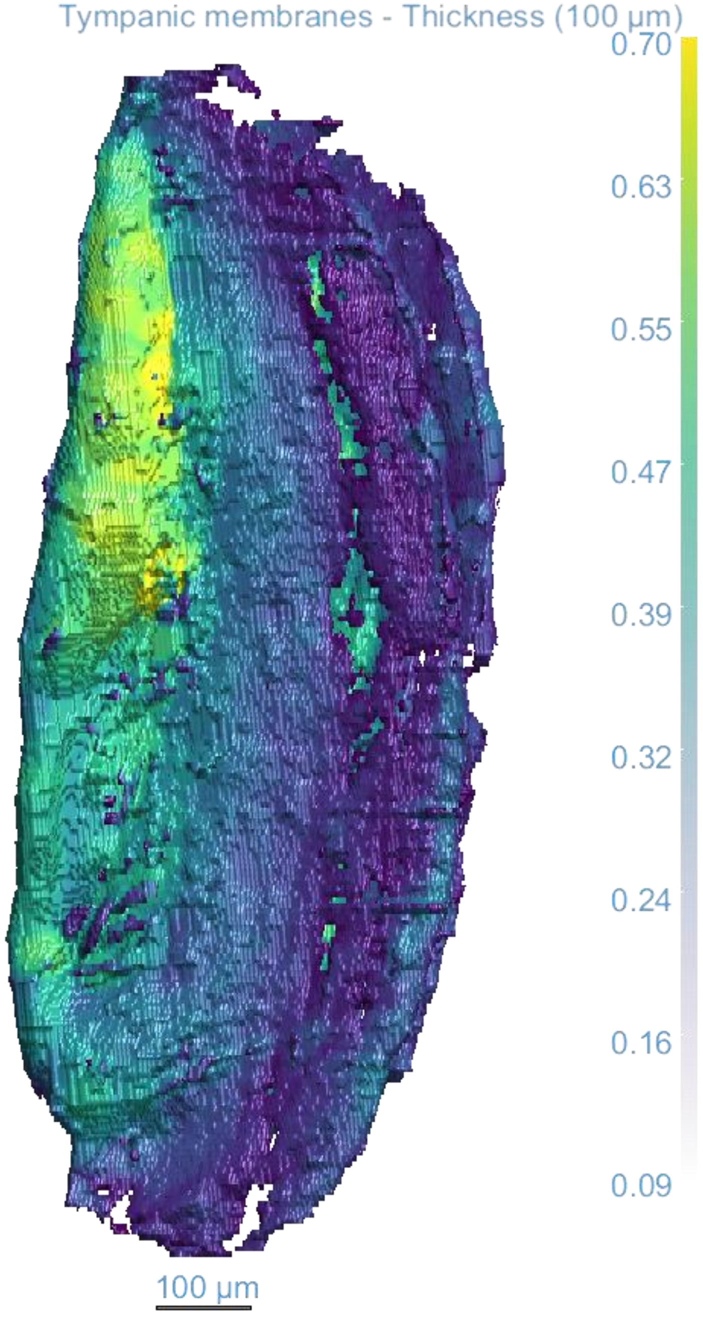

Phygela marginata

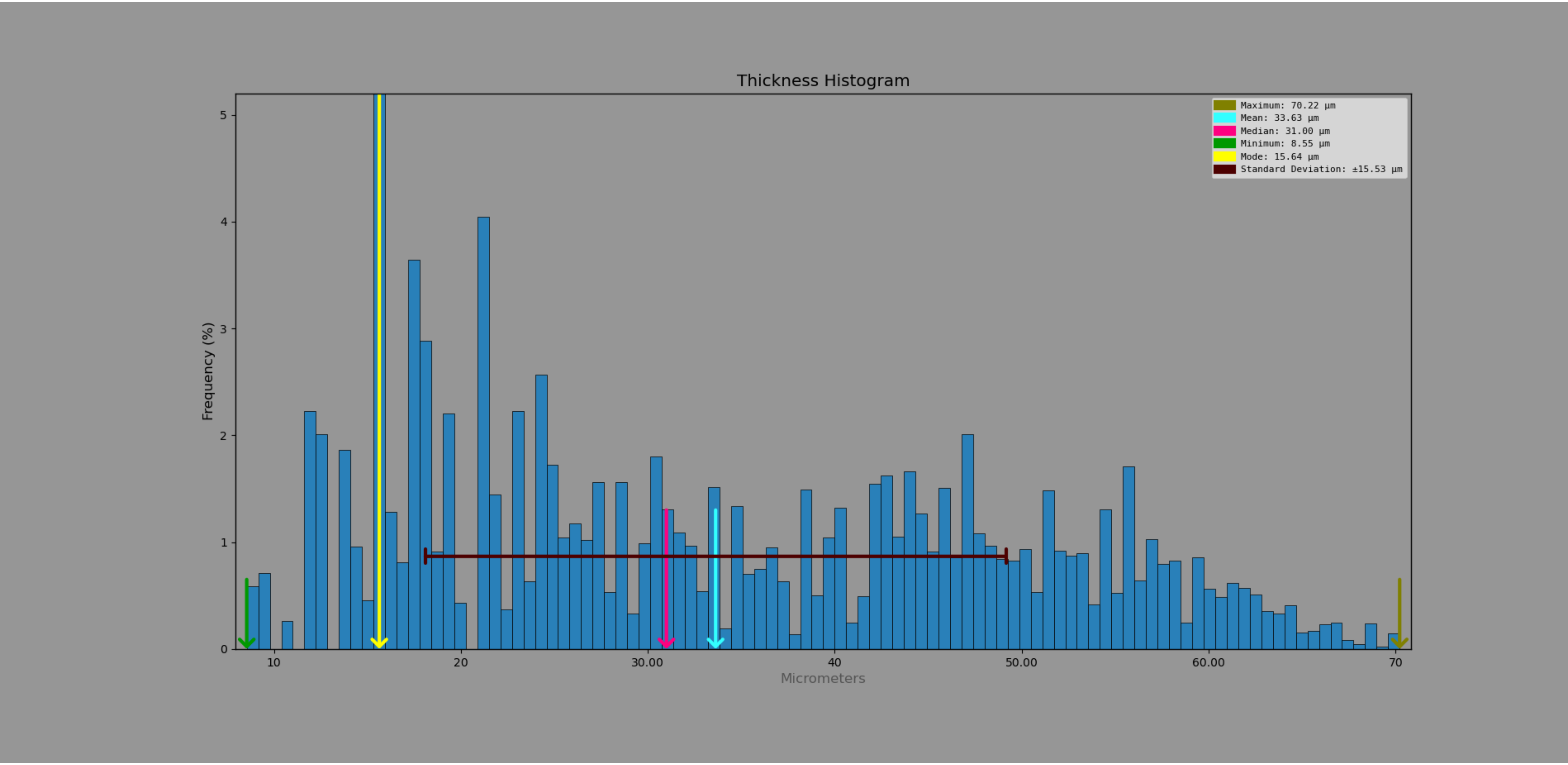

Phygela marginata - exposed tympana

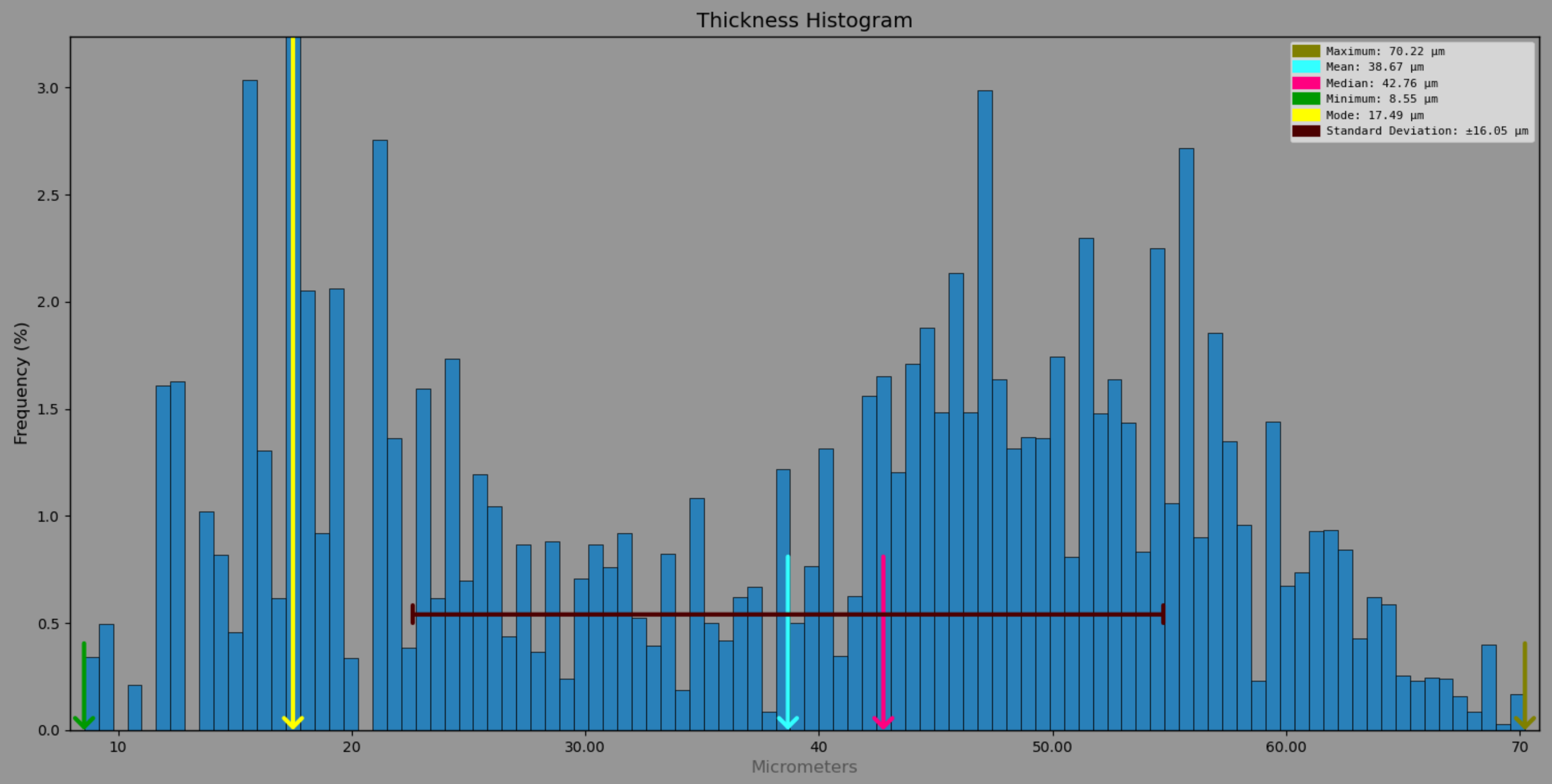

Phygela marginata – pinna covered tympana

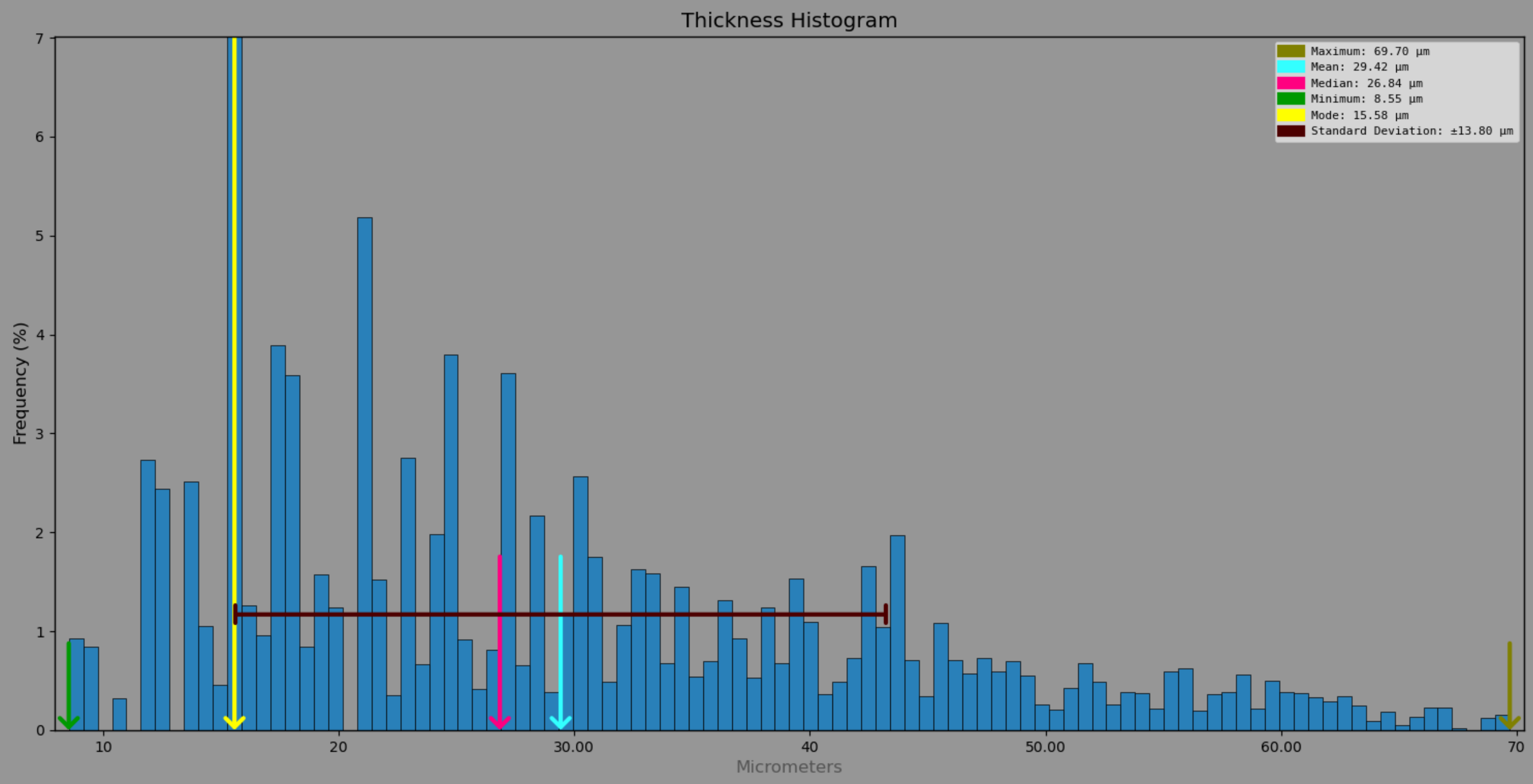

Phyllomimus detersus

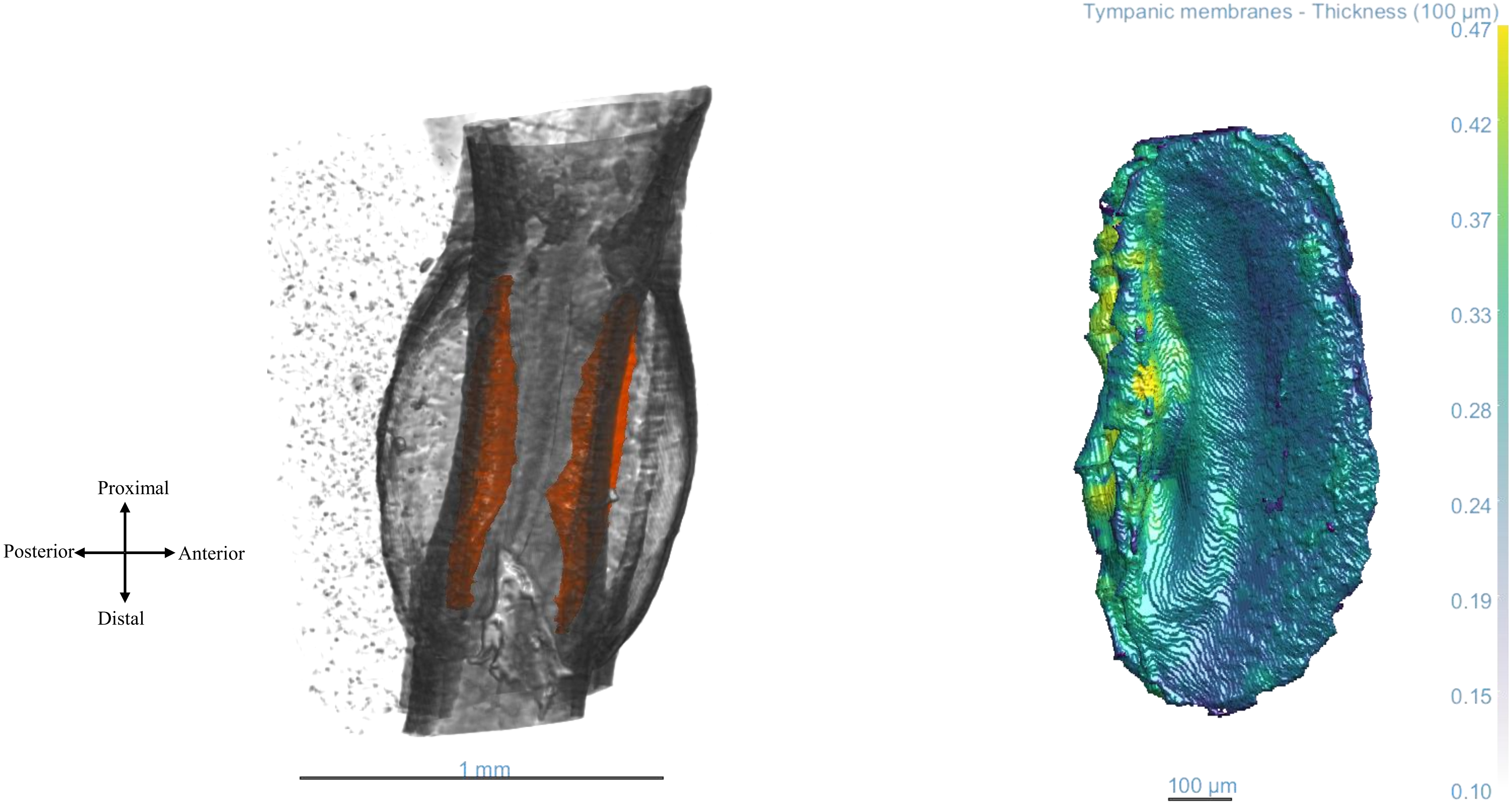

Phyllomimus detersus

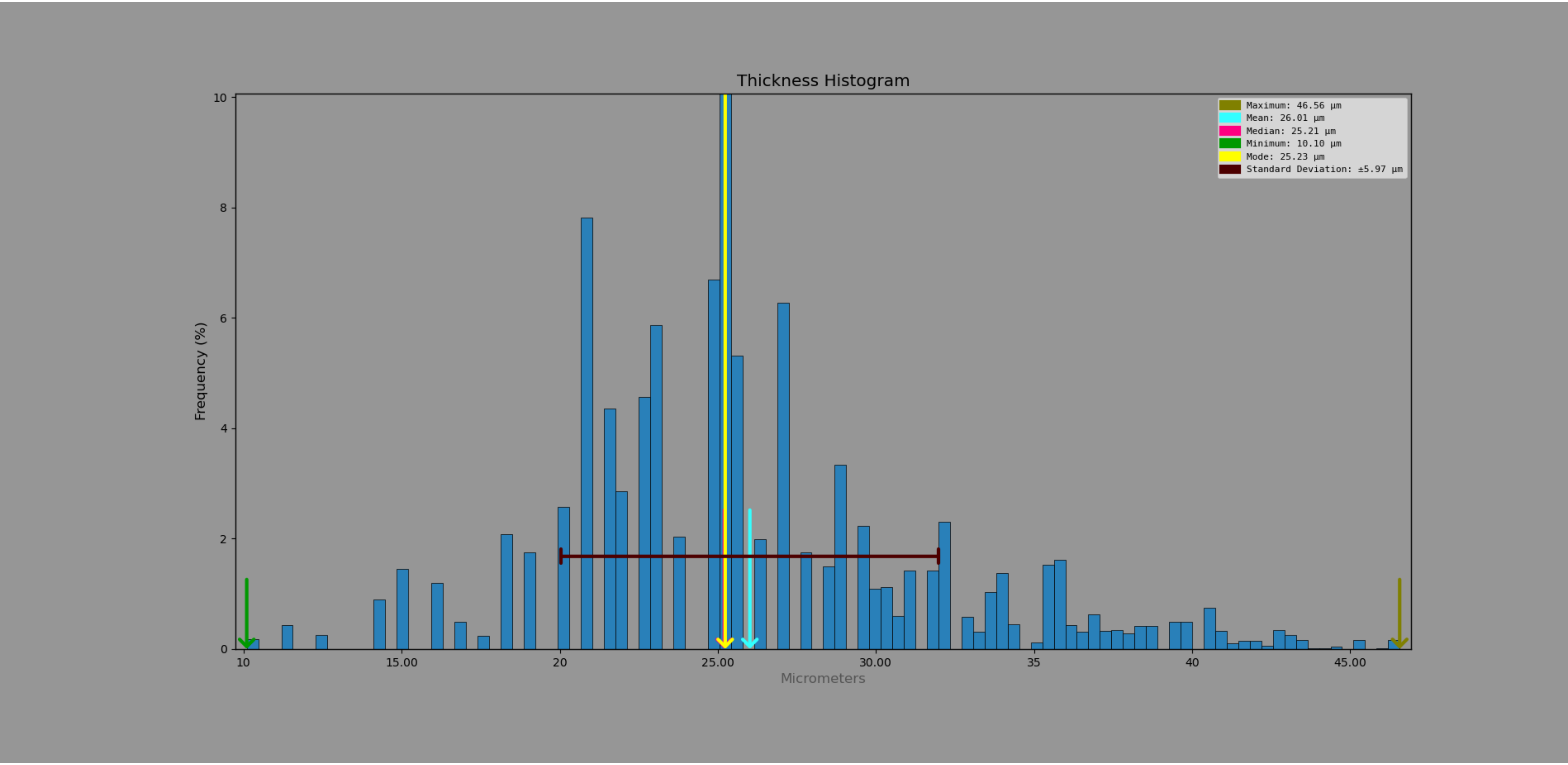

Ragoniella pulchella

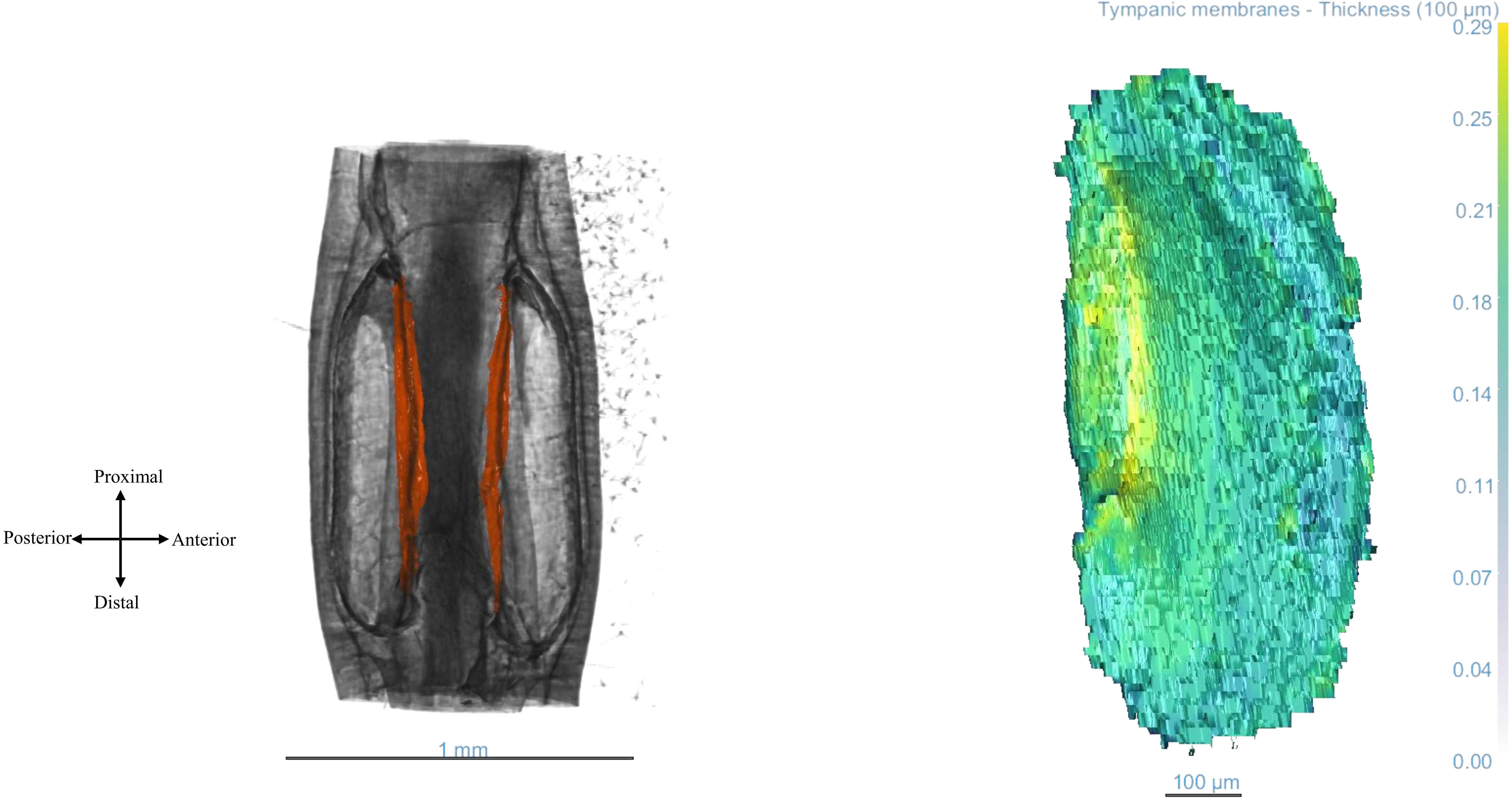

Ragoniella pulchella

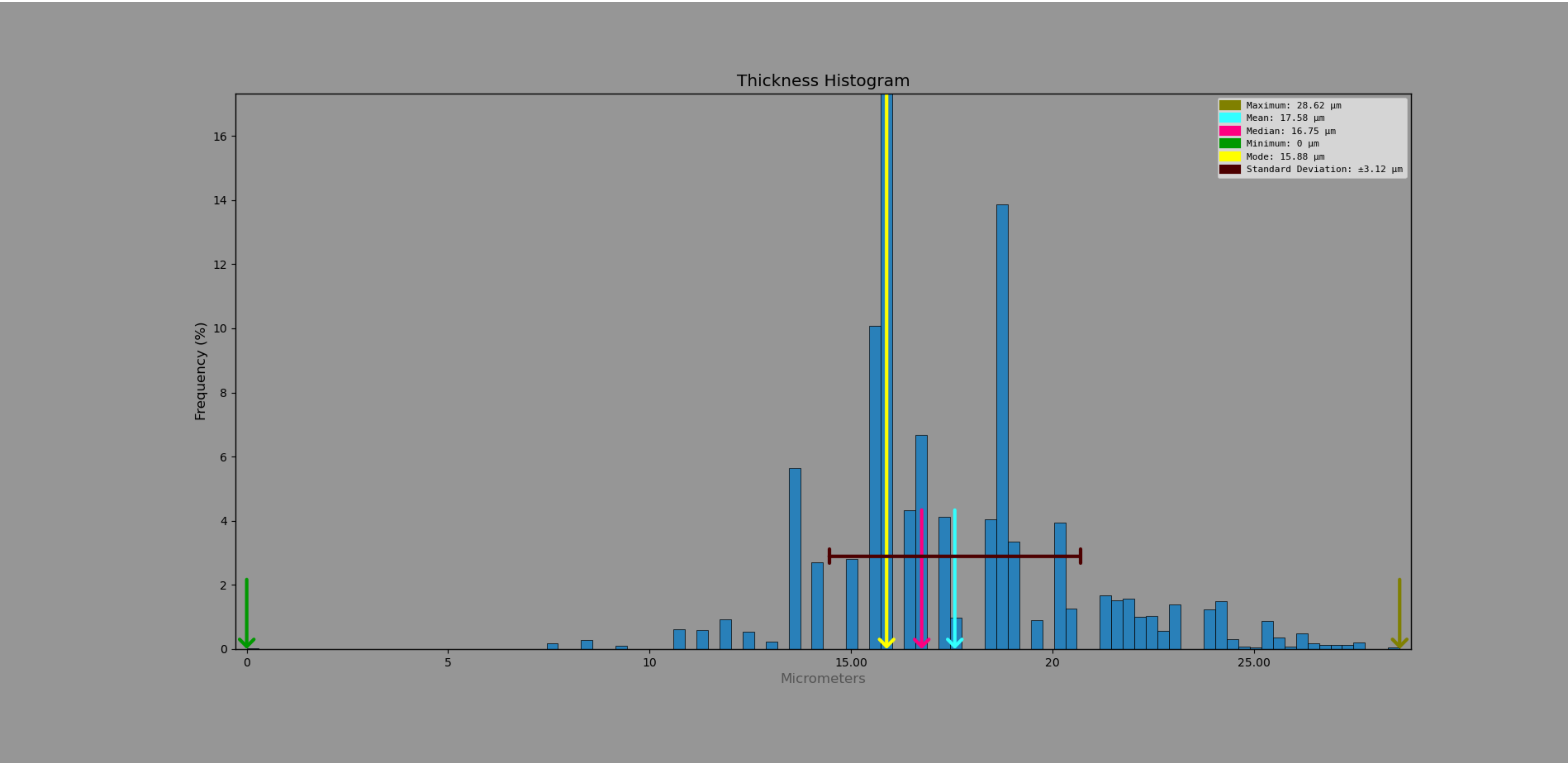

Satizabalus jorgevargasi

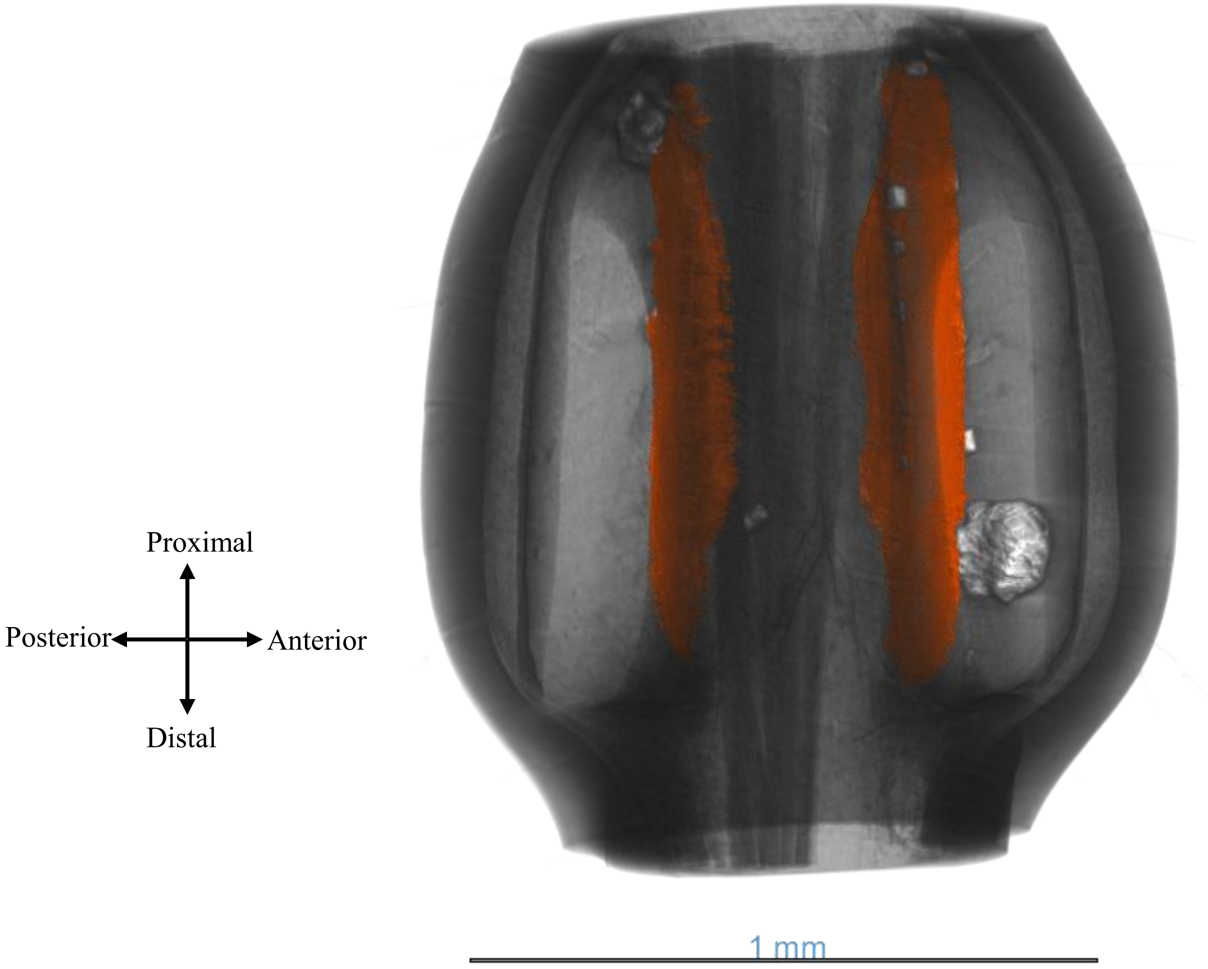

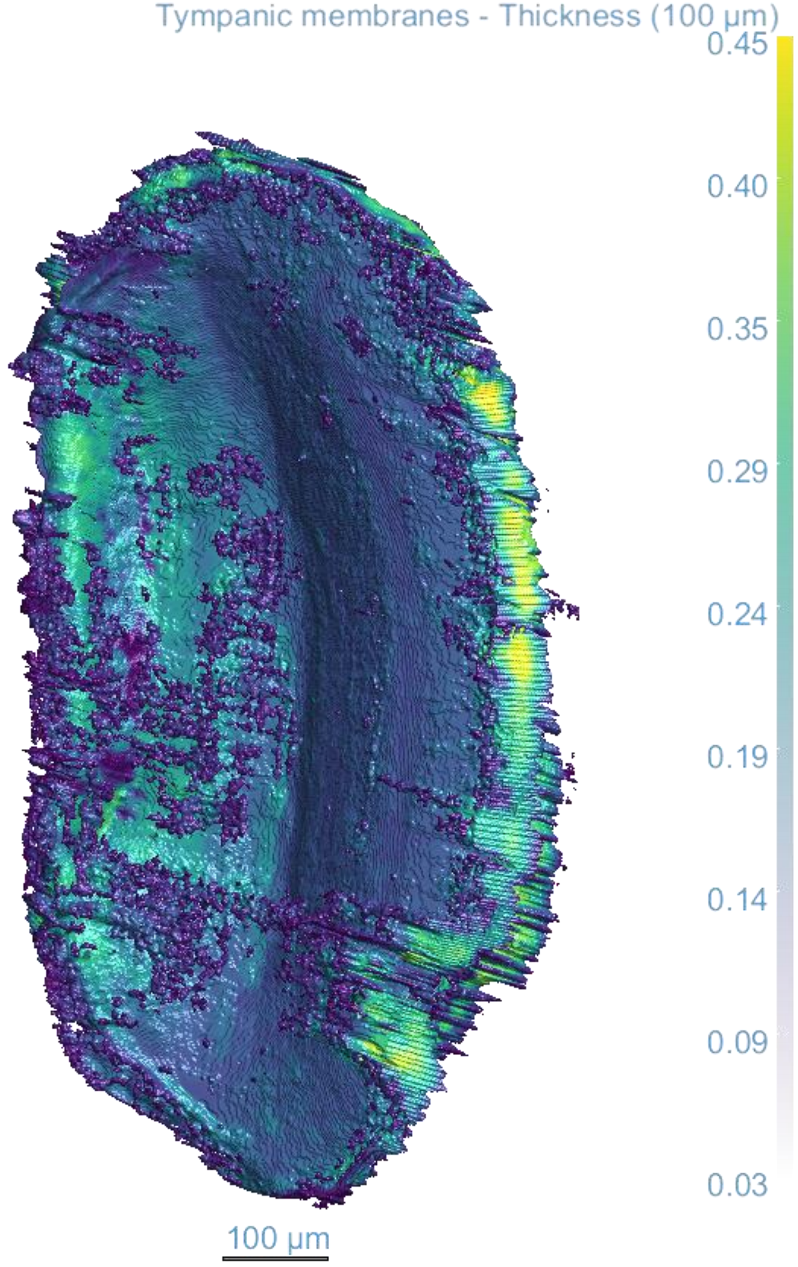

Satizabalus jorgevargasi

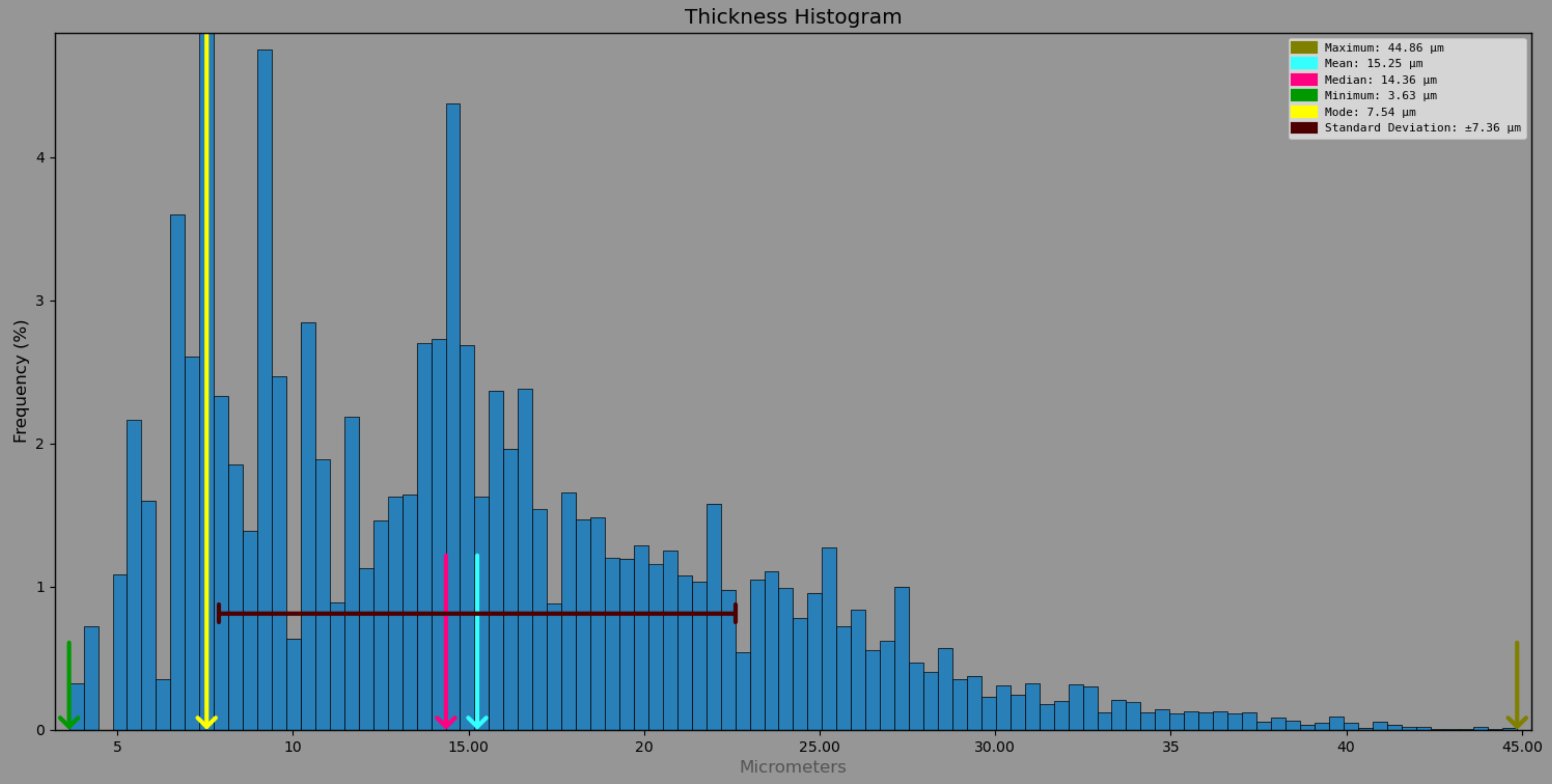

Stictophaula sp.

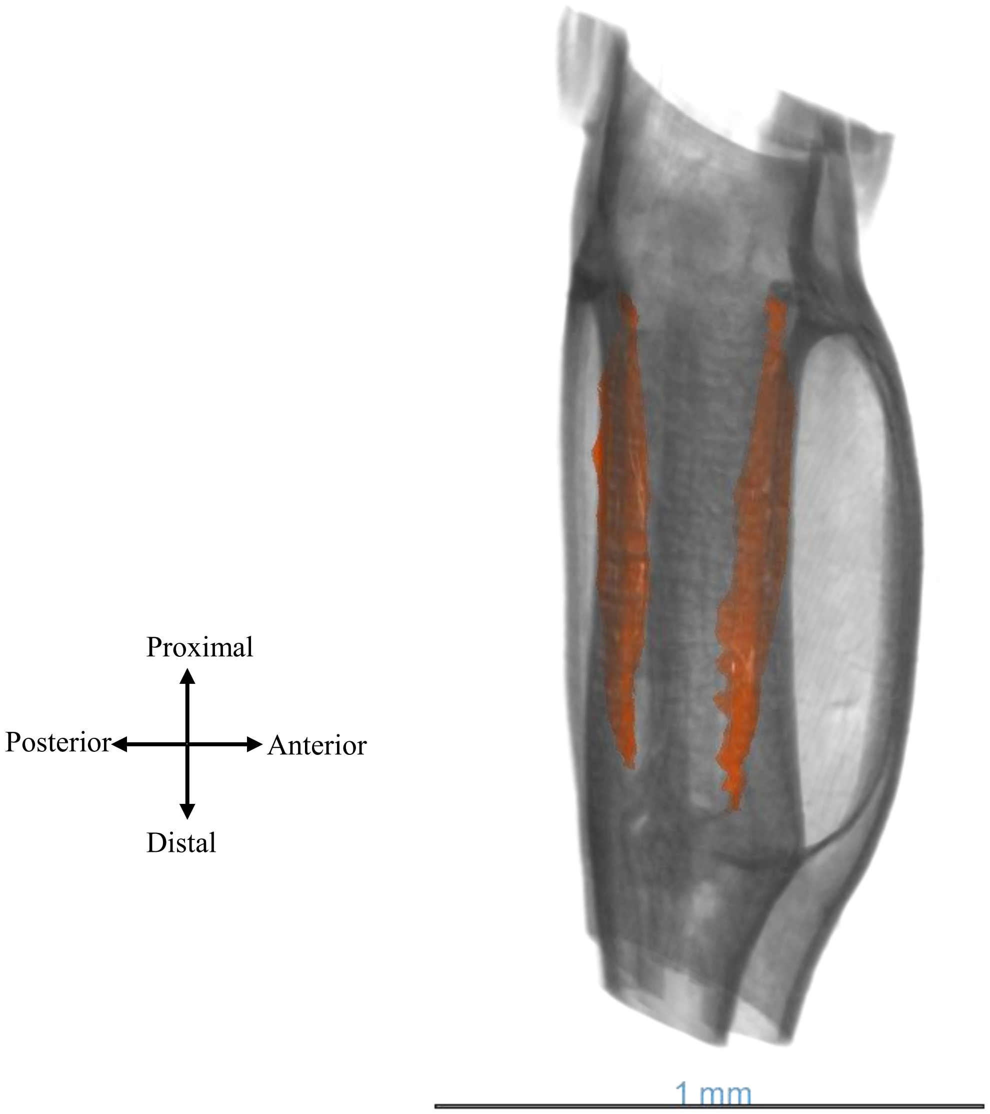

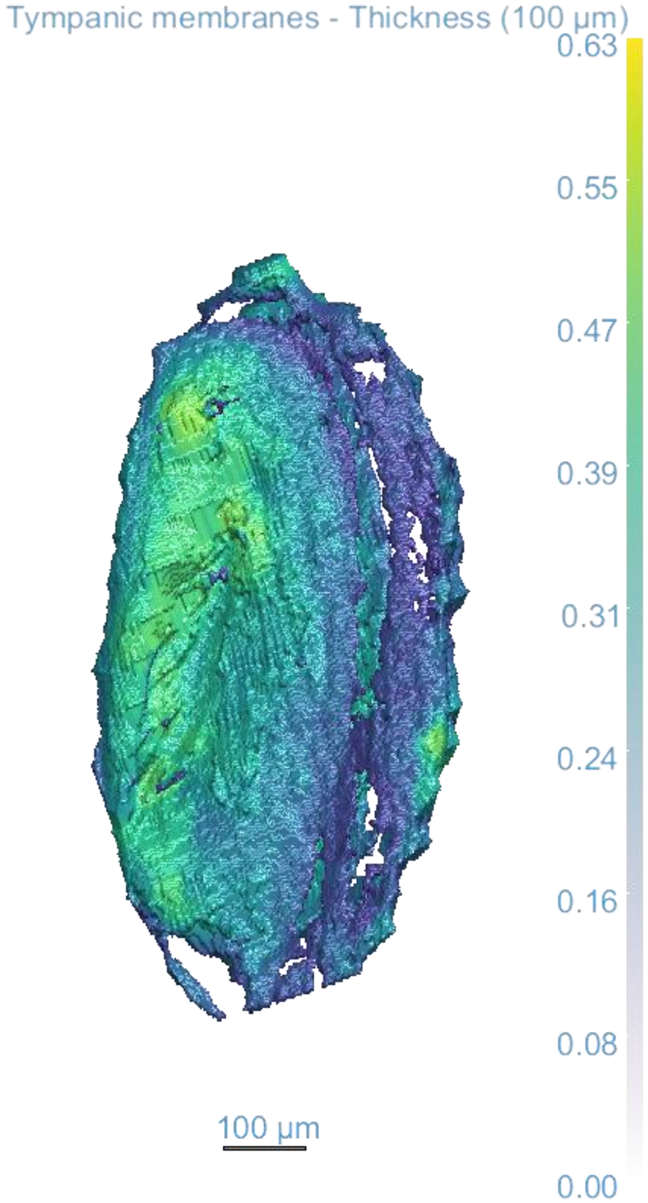

Stictophaula sp.

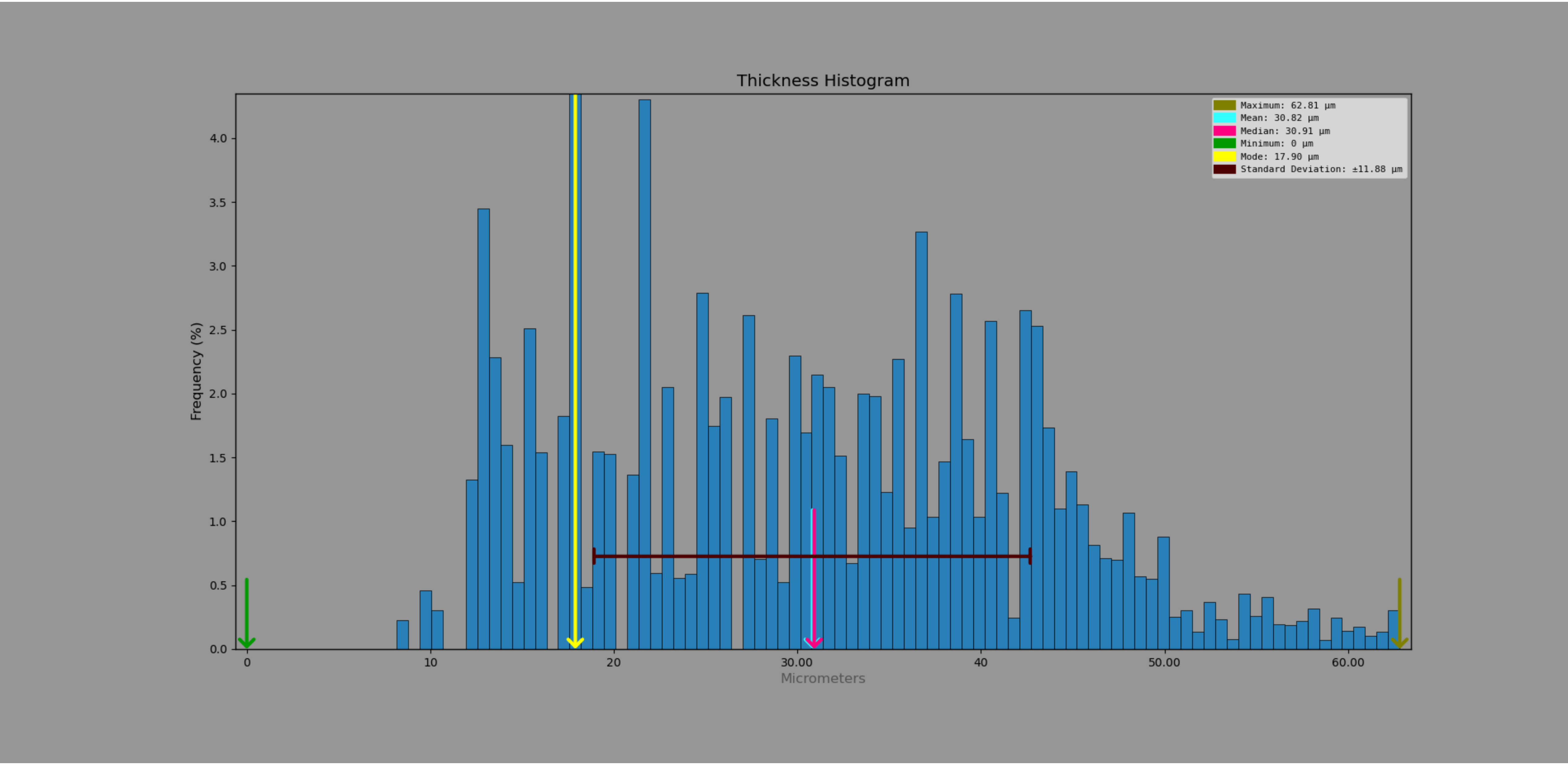

Stictophaula sp. - exposed tympana

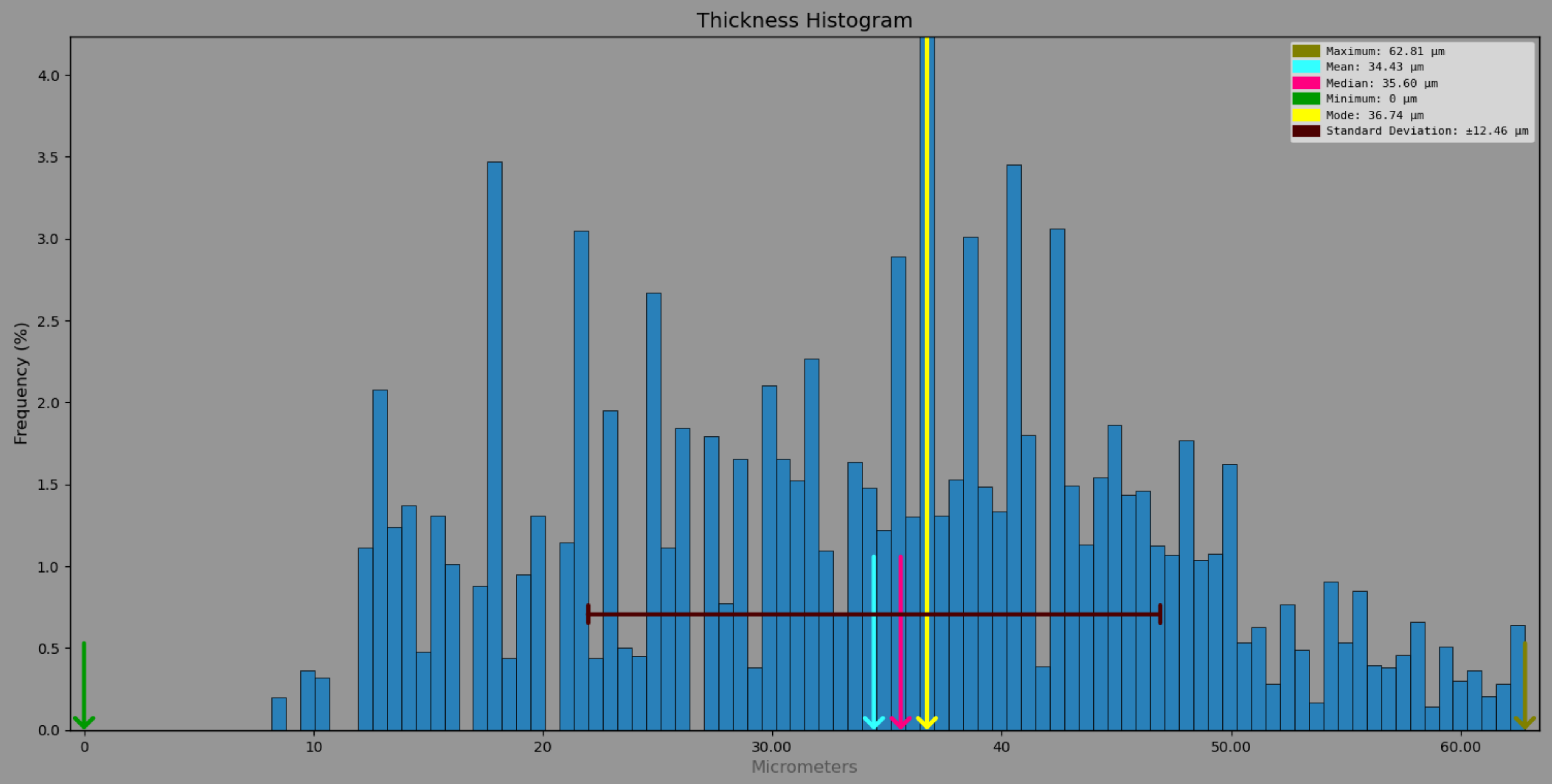

Stictophaula sp. – pinna covered tympana

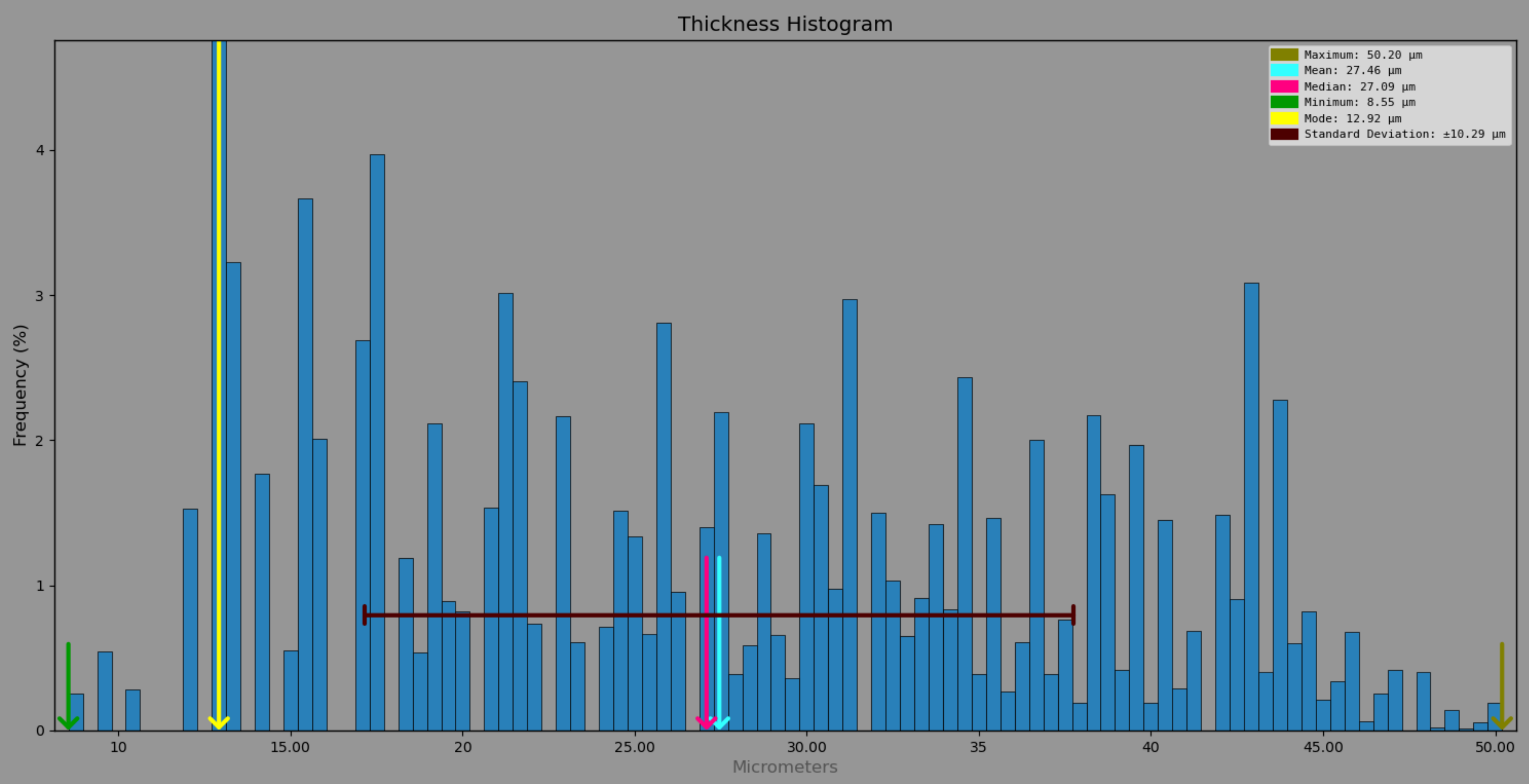

Stilpnochlora sp.

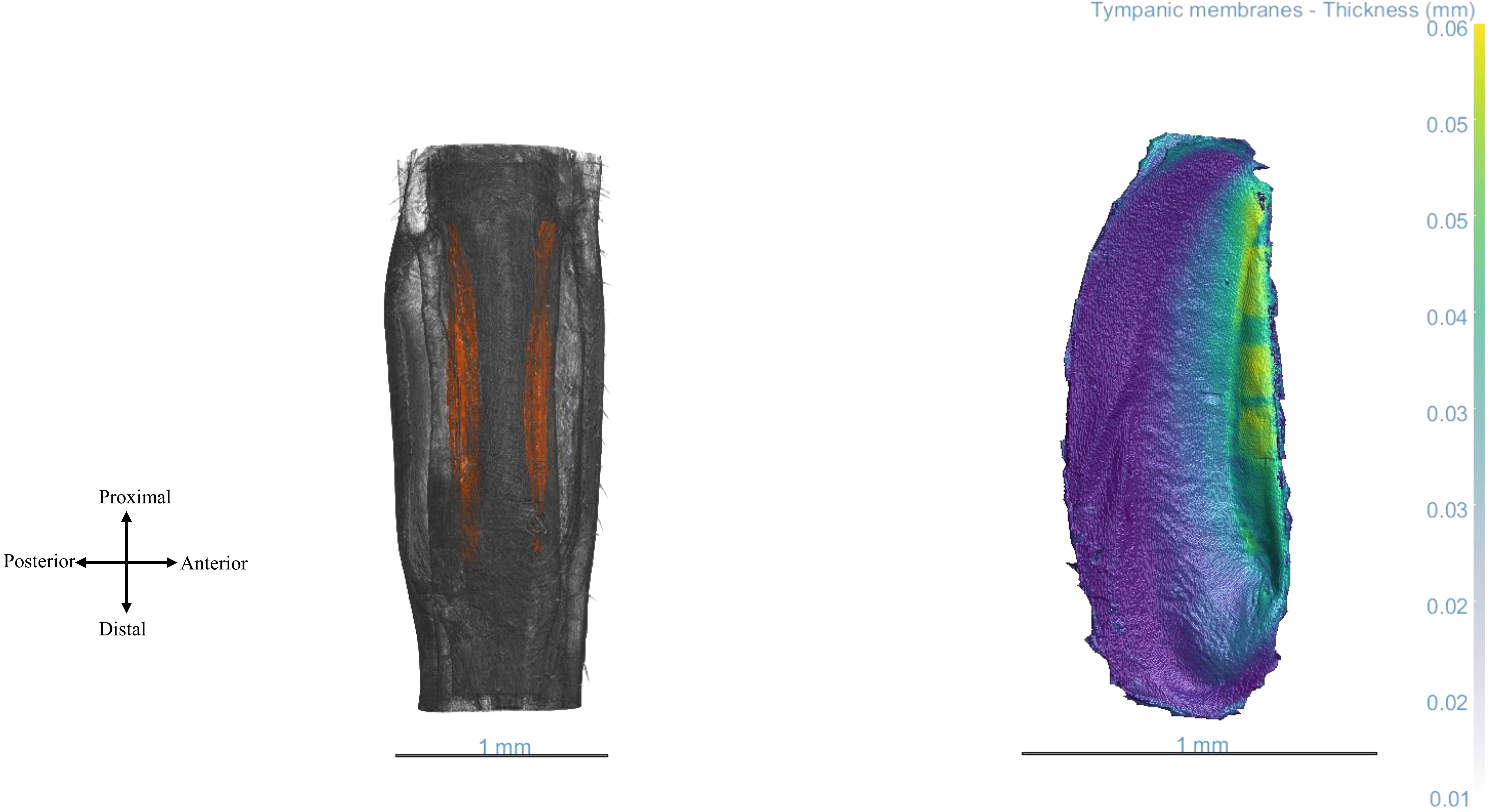

Stilpnochlora sp.

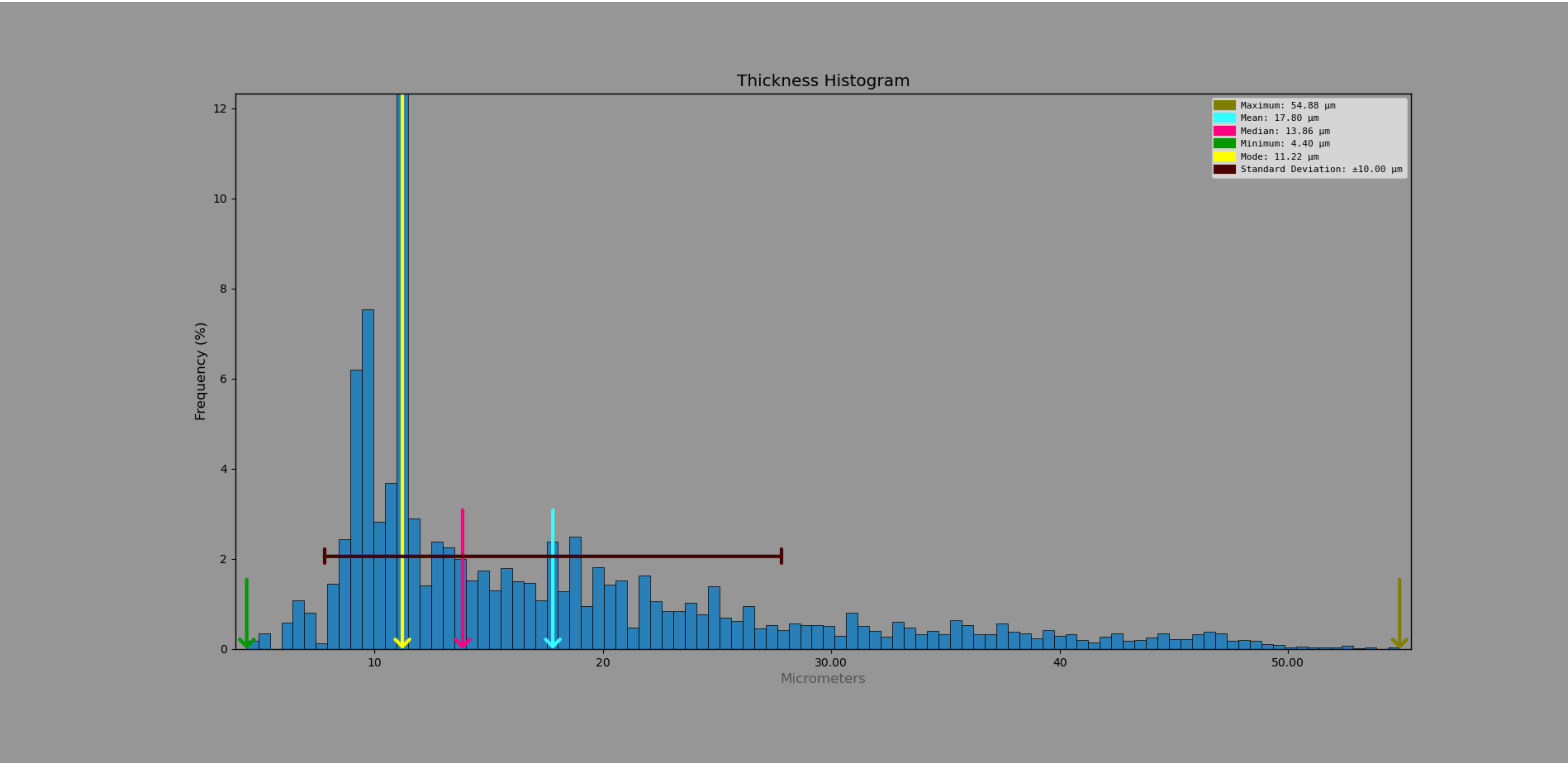

Supplement: Supplementary 1 — Supplementary Materials 1 and 2 Tables S1 to S3 Figs. S1 to S10 [file csbj.0035.f1.zip › Supplementary Material 2.pdf]
